# Supplementary material for: Systematic review of educational interventions to improve the menstrual health of young adolescent girls
Source: BMJ Open. 2022 Jun 8;12(6):e057204. doi: 10.1136/bmjopen-2021-057204 (PMC9185475; doi:10.1136/bmjopen-2021-057204)
Supplement: Supplementary data [file bmjopen-2021-057204supp001.pdf]

Supplementary File: Characteristics and quality of included studies

Citation	Design	Country	Study population	Sampling Method	No of participants	Type of intervention	MMAT Clear Research Questions /2	MMAT Appropriate Data Collection?/2	MMAT Supplementary questions relevant to methodology/5	MMAT Total/9	MMAT RAG rating
(1)	RCT quasi-experimental pre-test, post test	Iran	Mothers and adolescent school girls	Random Cluster-sampling	364	Half hour lecture and pamphlet distribution to improve attitude	2	1	5	8	Green
(2)	Quantitative Descriptive Case Series Quasi-experimental pre-test, post-test	Turkey	Intellectually-disabled girls in special education training centre	convenience	77	Demonstration on doll to improve Pad-replacement skills	2	2	4	8	Green
(3)	Quantitative descriptive one group pre-test, post-test	Iran	All adolescent girls in two welfare	Purposive sampling	30	Group counselling in boarding centres	2	2	3	7	Amber

			boarding centres								
(4)	Quantitative descriptive case report; pre-test, post-test	Indonesia	Mild-intellectually disabled girl	Purposive sampling	1	Stories and video-modelling	2	2	3	7	Amber
(5)	Mixed methods, quantitative survey pre-test, post-test	Ethiopia	Girls enrolled in school in grades 6 and 7	Cluster randomised approach	636	Puberty Book ‘Growth and Changes’	2	2	5	9	Green
(6)	RCT, pre-test, post-test	India	Adolescent high school girls aged 11 – 19 years of Zilla Parishad High School	Systematic random technique (every other girl on register)	250	Chalk and talk, posters, flipcharts,	1	2	2	5	Red
(7)	Quantitative descriptive one group, Pre-test, post-test	Bangladesh	adolescent female students aged 11–16 years, in grade 6–8, and living with their parents.	Random cluster sampling	416	Field manual training	2	2	3	7	Amber
(8)	Qualitative understanding of quasi-RCT	Uganda	school girls in eight study schools in primary classses grades 4 - 7.	Quasi-randomised cluster sampling	27: education (n=8) pads alone (n=8) education and pads (n=6) and	75 minute session of Straight Talk Education programme	2	2	5	9	Green

					control (n=5)						
(9)	RCT pre-test post-test	USA	Children of both sexes whose parents had registered an interest	Random sampling	80 (43 girls)	'A New You, that's Who' video series	2	2	3	7	Amber
(10)	3 arm RCT Control and 2 intervention: peer and small group teaching pre-test/post-test	Iran	Adolescent school girls	Random sampling of three high schools	90	Small group and peer-teaching	2	2	3	7	Amber
(11)	Mixed methods Longitudinal study	Uganda	Girls and boys in secondary school	Purposive selection of schools	369	Multi-component approach to optimising government guidelines for puberty education	2	2	5	9	Green
(12)	Quasi-experimental RCT, and pre-test, post- test	Iran	School girls	Random cluster sampling	152	A model-based educational program	1	2	2	5	Red
(13)	Quasi-experimental RCT	Iran	School girls aged 14 – 16 years and their parents	Random cluster sampling	159	Teaching programme with discussion	2	2	3	7	Amber
(14)	four-armed quasi-experimental RCT Pre and post test	Uganda	Girls in grades 3 - 5	Randomised	1124	Educational arm used Straight Talk	1	2	0	3	Amber

(15)	Mixed Methods Quantitative cross-sectional surveys and qualitative focus group	Nepal	Girls in grades seven to ten in 28 large schools.	Random cluster sampling	860	WASH in Schools (WinS) programme	1	2	4	7	Amber
(16)	Mixed methods process evaluation Longitudinal study	Uganda	Girls and boys in secondary school	Purposive selection of schools	369	Multi-component approach to optimising government guidelines for puberty education	2	2	5	9	Green
(17)	3-arm single-site open cluster randomised controlled pilot study.	Kenya	Primary girls aged 14 -16	Random cluster sampling	644	Puberty education and menstrual cup instruction from nurse	1	2	5	8	Green
(18)	Quantitative non-random post-test only	India	Adolescent girls	Purposive (schools) and random (girls)	2206	Monthly discussion group	2	2	5	9	Green
(19)	Quasi-experimental Control/intervention pre-test post-test	Indonesia	Girls aged 9-12 pre-menarch	Stratified random sampling technique	174	Booklet	2	2	2	6	Amber
(20)	Experimental; Intervention and control; pre-test, post-test	India	Adolescent girls at a government high school	Randomly sampled	50	One training session on menstrual hygiene	1	0	1	2	Red
(21)	Quantitative descriptive Pre-test, post-test	India	Adolescent girls	Multi-level stratified	2564	‘model’ schools received	1	2	5	8	Green

				sampling of schools		additional WASH support and education embedded into curriculum					
(22)	A quasi-experimental RCT two group pretest-posttest design	China	Adolescent girls	Purposive sampling of schools	116	Researcher – led Menstrual Hygiene Class	2	2	4	8	Green
(23)	Mixed methods. Qualitative review and semi-quantitative measure of cup use nested in larger RCT	Kenya	Post-menarche adolescent girls	Random allocation of 10 schools to menstrual cup arm	192	Puberty education and menstrual cup instruction from nurse	1	2	5	8	Green
(24)	RCT three arm – educating girls, educating mothers or control. pre and post test	Iran	Post-menarche adolescent school girls	12 purposively sampled schools	327	30 minute lecture on puberty hygiene plus a booklet	0	1	5	6	Amber

Summary of characteristics of included studies

Framework analysis and the 'best fit' principle were used to score the studies (25–27). All studies were interrogated with two questions 'Are there clear research questions?' and 'Do the collected data allow the research questions to be addressed?' which were considered fundamental to the quality and were scored on a scale of 'Yes' = 2, 'not clear' = 1 and 'No' = 0. Five further supplementary questions were considered that addressed quality issues such as sample size. The sets of questions were different depending upon the study design, and are not directly comparable, so less weight was given to these; Yes = 1 and No = 0. The maximum score when added together was 2 + 2 + 5 = 9. Studies scored 0-5 were categorised as low quality (as it was possible to get these scores without clear research questions or valid methods); those that scored 6 or 7 were scored as moderate quality and those that scored 8 or 9 were scored as high quality (a subjective scale based on personal expertise (27) and community of practice validation (28).

REFERENCES

1. Afsari A, Mirghafourvand M, Valizadeh S, Abbasnezhadeh M, Galshi M, Fatahi S. The effects of educating mothers and girls on the girls' attitudes toward puberty health: A randomized controlled trial. Int J Adolesc Med Health. 2015;2015.

2. Altundağ S, Çalbayram NÇ. Teaching menstrual care skills to intellectually disabled female students. Mol Ecol. 2016;25(13–14):1962–8.

3. Arasteh FE, Shobeiri F, Parsa P, Mohamadi Y. Effect of group counseling on adolescent girls in improving knowledge and practice of menstrual hygiene in welfare boarding centers. J Postgrad Med Inst. 2019;33(1):46–51.

4. Ariyanti TD, Royanto LRM. The Effectiveness of Social Stories and Video Modeling in Improving Self-Care Skills in Female Adolescents with Mild Intellectual Disabilities during Menstrual Periods. 2018;135(Iciap 2017):189–99.

5. Blake S, Boone M, Yenew Kassa A, Sommer M. Teaching Girls About Puberty and Menstrual Hygiene Management in Rural Ethiopia: Findings From a Pilot Evaluation. J Adolesc Res. 2018;33(5):623–46.

6. Rani Chadalawada U, Devi S A, Rani M S. Effect of Health Education on Adolescent Girls Regarding Knowledge About Menstruation. J Evol Med Dent Sci. 2017;6(13):1040–3.

7. Haque SE, Rahman M, Itsuko K, Mutahara M, Sakisaka K. The effect of a school-based educational intervention on menstrual health: An intervention study among adolescent girls in Bangladesh. BMJ Open. 2014;4(7):1–9.

8. Hennegan J, Montgomery P. Do menstrual hygiene management interventions improve education and psychosocial outcomes for women and girls in low and middle income countries? A systematic review. PLoS One. 2016;11(2):1–22.

9. Hurwitz LB, Lovato SB, Lauricella AR, Woodruff TK, Patrick E, Wartella E. "A New You, That's Who": an evaluation of short videos on puberty and human reproduction. Palgrave Commun. 2018;4(1).

10. Jarrahi R, Golmakani N, Mazlom SR. Effect of menstrual hygiene education based on peer and small group teaching methods on hygiene behaviors in female adolescents: A comparative study. Evid Based Care J. 2020;10(1):70–4.

11. Kansiime C, Hytti L, Nalugya R, Nakuya K, Namirembe P, Nakalema S, et al. Menstrual health intervention and school attendance in Uganda (MENISCUS-2): A pilot intervention study. BMJ Open. 2020;10(2):1–11.

12. Kheirollahi F, Rahimi Z, Arsang-Jang S, Sharifirad G, Sarraf P, Gharlipour Z. Puberty health status among adolescent girls: A model- based educational program. Int J Pediatr. 2017;5(7):5369–78.

13. Mokari H, Khaleghparast S, Samani LN. Impact of Puberty Health Education on Anxiety of Adolescents. Int J Med Res Heal Sci. 2016;5(5, S, SI):284–91.

14. Montgomery P, Hennegan J, Dolan C, Wu M, Steinfield L, Scott L. Menstruation and the Cycle of Poverty: A Cluster Quasi-Randomised Control Trial of Sanitary Pad and Puberty Education Provision in Uganda. PLoS One. 2016;11(12).

15. Morrison J, Basnet M, Bhatt A, Khimbanjar S, Chaulagain S, Sah N, et al. Girls' menstrual management in five districts of Nepal: Implications for policy and practice. Stud Soc Justice. 2018;12(2):251–72.

16. Nalugya R, Tanton C, Hytti L, Kansiime C, Nakuya K, Namirembe P, et al. Assessing the effectiveness of a comprehensive menstrual health intervention program in Ugandan schools (MENISCUS): process evaluation of a pilot intervention study. Pilot Feasibility Stud. 2020;6(1):1–15.

17. Phillips-Howard PA, Nyothach E, Ter Kuile FO, Omoto J, Wang D, Zeh C, et al. Menstrual cups and sanitary pads to reduce school attrition, and sexually transmitted and reproductive tract infections: A cluster randomised controlled feasibility study in rural Western Kenya. BMJ Open. 2016;

18. Ramaiya A, Malhotra A, Cronin C, Stevens S, Kostizak K, Sharma A, et al. How does a Social and Behavioral Change Communication Intervention Predict Menstrual Health and Hygiene Management: A Cross-Sectional Study. BMC Public Health. 2019;19(1):1–12.

19. Setyowati S, Rizkia M, Ungsianik T. Improving Female Adolescents' Knowledge, Emotional Response, and Attitude toward Menarche following Implementation of Menarcheal Preparation Reproductive Health Education. Asian/Pacific Isl Nurs J. 2019;4(2):84–91.

20. Sharma R, Sandhya N, Deepika K, Varsha S, Vardha. Menstrual hygiene among adolescent girls. Indian J Community Heal. 2015;27(3):376–80.

21. Sivakami M, van Eijk AM, Thakur H, Kakade N, Patil C, Shinde S, et al. Effect of menstruation on girls and their schooling, and facilitators of menstrual hygiene management in schools: Surveys in government schools in three states in India, 2015. J Glob Health. 2019;9(1).

22. Su JJ, Lindell D. Promoting the menstrual health of adolescent girls in China. Nurs Heal Sci. 2016;18(4):481–7.

23. Van Eijk AM, Sivakami M, Thakkar MB, Bauman A, Laserson KF, Coates S, et al. Menstrual hygiene management among adolescent girls in India: A Systematic review and meta-analysis. BMJ Open. 2016;6(3).

24. Valizadeh S, Assdollahi M, Mirghafourvand M, Afsari A. Educating Mothers and Girls about Knowledge and Practices toward Puberty Hygiene in Tabriz, Iran: A Randomized Controlled Clinical Trial. Iran Red Crescent Med J. 2017;19(2).

25. Carroll C, Booth A, Cooper K. A worked example of "best fit" framework synthesis: A systematic review of views concerning the taking of some potential chemopreventive agents. BMC Med Res Methodol. 2011;11.

26. Carroll C, Booth A, Leaviss J, Rick J. "best fit" framework synthesis: Refining the method. BMC Med Res Methodol. 2013;13(1).

27. Suto I. A critical review of some qualitative research methods used to explore rater cognition. Educ Meas Issues Pract. 2012;31(3):21–30.

28. Bejar II. A validity-based approach to quality control and assurance of automated scoring. Assess Educ Princ Policy Pract. 2011;18(3):319–41.
