# Supplementary material for: Systematic review of educational interventions to improve the menstrual health of young adolescent girls
Source: BMJ Open. 2022 Jun 8;12(6):e057204. doi: 10.1136/bmjopen-2021-057204 (PMC9185475; doi:10.1136/bmjopen-2021-057204)
Supplement: Author's manuscript [file bmjopen-2021-057204.draft_revisions.pdf]

BMJ Open

BMJ Open is committed to open peer review. As part of this commitment we make the peer review history of every article we publish publicly available.

When an article is published we post the peer reviewers' comments and the authors' responses online. We also post the versions of the paper that were used during peer review. These are the versions that the peer review comments apply to.

The versions of the paper that follow are the versions that were submitted during the peer review process. They are not the versions of record or the final published versions. They should not be cited or distributed as the published version of this manuscript.

BMJ Open is an open access journal and the full, final, typeset and author-corrected version of record of the manuscript is available on our site with no access controls, subscription charges or pay-per-view fees (http://bmjopen.bmj.com).

If you have any questions on BMJ Open's open peer review process please email info.bmjopen@bmj.com

BMJ Open

A Systematic Review of Educational Interventions to improve the Menstrual Health of young adolescent girls.

Journal:	*BMJ Open*
Manuscript ID	bmjopen-2021-057204
Article Type:	Original research
Date Submitted by the Author:	08-Sep-2021
Complete List of Authors:	Evans, Rebecca; University of Warwick<br>Harris, Bronwyn; Warwick Medical School, University of Warwick, Coventry, Division of Health Sciences, ; University of the Witwatersrand, Centre for Health Policy, School of Public Health<br>Griffiths, Frances; University of Warwick, Warwick Medical School<br>Onuegbu, Chinwe ; University of Warwick, Health Sciences
Keywords:	PUBLIC HEALTH, Health policy < HEALTH SERVICES ADMINISTRATION & MANAGEMENT, REPRODUCTIVE MEDICINE, PREVENTIVE MEDICINE

SCHOLARONE™
Manuscripts

BMJ

I, the Submitting Author has the right to grant and does grant on behalf of all authors of the Work (as defined in the below author licence), an exclusive licence and/or a non-exclusive licence for contributions from authors who are: i) UK Crown employees; ii) where BMJ has agreed a CC-BY licence shall apply, and/or iii) in accordance with the terms applicable for US Federal Government officers or employees acting as part of their official duties; on a worldwide, perpetual, irrevocable, royalty-free basis to BMJ Publishing Group Ltd ("BMJ") its licensees and where the relevant Journal is co-owned by BMJ to the co-owners of the Journal, to publish the Work in this journal and any other BMJ products and to exploit all rights, as set out in our licence.

The Submitting Author accepts and understands that any supply made under these terms is made by BMJ to the Submitting Author unless you are acting as an employee on behalf of your employer or a postgraduate student of an affiliated institution which is paying any applicable article publishing charge ("APC") for Open Access articles. Where the Submitting Author wishes to make the Work available on an Open Access basis (and intends to pay the relevant APC), the terms of reuse of such Open Access shall be governed by a Creative Commons licence – details of these licences and which Creative Commons licence will apply to this Work are set out in our licence referred to above.

Other than as permitted in any relevant BMJ Author's Self Archiving Policies, I confirm this Work has not been accepted for publication elsewhere, is not being considered for publication elsewhere and does not duplicate material already published. I confirm all authors consent to publication of this Work and authorise the granting of this licence.

For peer review only

[Type here]

TITLE PAGE

Title:

A systematic Review of Educational Interventions to improve the Menstrual Health of young adolescent girls.

Authors:
Ms Rebecca Lane-Evans, Warwick Medical School, Warwick Medical School, University of Warwick, Coventry, CV4 7AL United Kingdom. Rebecca.R.Evans@warwick.ac.uk

Ms Chinwe Onuegbu, Warwick Medical School, Warwick Medical School, University of Warwick, Coventry, CV4 7AL United Kingdom. Chinwe.Onuegbu@warwick.ac.uk

Dr Bronwyn Harris, Warwick Medical School, Warwick Medical School, University of Warwick, Coventry, CV4 7AL United Kingdom. B.Harris.2@warwick.ac.uk

Professor Frances Griffiths, Warwick Medical School, Warwick Medical School, University of Warwick, Coventry, CV4 7AL United Kingdom. F.E.Griffiths@warwick.ac.uk

Word Count: 3955

For peer review only

For peer review only - http://bmjopen.bmj.com/site/about/guidelines.xhtml

[Type here]

ABSTRACT

Objectives

To systematically review interventions that include an element of menstrual education delivered to young adolescents and inexperienced menstruators.

Design

This was a systematic review and meta-analysis. Selected articles were quality assessed using the Mixed Methods Assessment Tool (MMAT) quality appraisal checklist. A meta-analysis was conducted on a subset of articles that provided quantitative results, and the effect size of the intervention was calculated using Cohen's d. A logic model was constructed to frame the effect of Menstrual Education Interventions on Menstrual Health.

Setting

Seven wide-ranging electronic databases were searched for English-language entries that evaluated interventions carried out in High and Low-and-Middle Income Countries and were published between January 2014 and May 2020.

Participants

The interventions were aimed primarily at younger adolescents aged 10 -14 years old

Interventions

The interventions were designed to improve the Menstrual Health of the recipients, by addressing one or more elements of menstrual Knowledge, Attitude or Practices (KAP).

Primary and Secondary Outcomes

The most common type of output was a difference in knowledge or skill score ascertained from a pre- and post-test. Some studies measured additional outcomes, such as attitude or confidence.

Results

Twenty-four eligible studies were identified. Only one was from a High-income country. Twenty were aimed specifically at schoolgirls. Nineteen addressed Menstrual Knowledge, and five addressed Menstrual Skills such as pad replacement or cup insertion. All studies reported improvements in menstrual KAP. The meta-analysis indicates that larger effect sizes were attained with the more interactive interventions that encouraged discussion of the topic.

Conclusions

Education interventions are effective in increasing the menstrual knowledge of young adolescent girls and skills training improves competency to manage menstruation more hygienically and comfortably. Where measured, confidence improved and indeed, all indicators were positive. It could be that the level of menstrual awareness in these studied populations is so low at base line that any intervention makes a difference. This would suggest that there is a pressing need for menstrual education.

[Type here]

For peer review only

[Type here]

STRENGTHS AND LIMITATIONS

- This review is of the international literature including high and low-and-middle-income countries
- It utilises mixed methods approaches and synthesises both quantitative and qualitative studies
- A logic model has been constructed to frame the effect of Menstrual Education Interventions on Menstrual Health
- There may be publication bias in the reporting of positive outcomes only
- A full meta-analysis was not possible due to heterogeneity in methods

For peer review only

INTRODUCTION

Globally, young adolescents are ill-prepared for menarche and menstruation (1–4). In many cultures, menstruation is a taboo subject (5) and many girls are ignorant of it until they start bleeding (6). Negative experiences of menarche and early menstruation can cause poor Menstrual Health (7,8).

'Menstrual Health' is an emerging area of Health Research. It is a broad term which encompasses the hygienic management of menstruation and the psychological components of well-being such as confidence, dignity and self-esteem (9,10). It is an expansion of the concept of Menstrual Hygiene Management (MHM), mostly used in Low- and Middle- Income Country (LMIC) contexts to describe the challenges of hygienically managing menstruation with a lack of resources, especially pads, water and soap (11),(12,13). The use of dirty rags to absorb menstrual blood has been proposed as a cause of Reproductive Tract Infections (RTIs) and cervical cancer (14–16), as infectious agents may be introduced into the reproductive tract from such materials. Even clean materials that are not changed regularly may smell (3) and some materials can cause chafing and irritation. Without sufficient water to wash the blood from their genitals or their hands (17) girls can become uncomfortable and anxious (18). Several studies in LMIC have shown that menstruation is associated with a reduction in participation in activities and an increase in school absenteeism (15,19,20). In High Income Countries (HIC), 'Period Poverty' has only recently been recognised as an issue for certain groups, such as homeless women (21) but a whitepaper by PHS Group UK (a Hygiene Services provider in the UK) suggests period poverty is more wide-reaching, particularly amongst school girls, and is a factor contributing to menstrual anxiety and school absenteeism (22). Significantly less research has been conducted in HICs around menstruation and participation, although it is widely recognised that girls drop out of sports activities around the age of puberty (23,24).

Discussing menstruation is almost universally a taboo, making it difficult for girls to learn about it and know what is normal or when to seek help. A study by Gultie *et al* 2014 into Menstrual Knowledge of adolescents in Ethiopia found that 33% of participants never talked about it with anyone (25). In Jordan, a phenomenological analysis of experiences of menarche reported that girls believed talking about menstruation was 'rude' (26). A study into puberty communication in the Czech Republic and China found that both men and women were complicit in perpetuating

[Type here]

menstrual stigma, with mothers instructing their daughters to keep their menstrual status 'secret' (27). In India, Rani *et al* 2016 found that although 61.3% of adolescent girls suffered from debilitating dysmenorrhea, they felt they were expected to 'tolerate' it as a natural process and only 1.6% had ever consulted a physician (14).

Menstrual knowledge usually comes from mothers (8) but a number of studies have shown that the knowledge of mothers themselves may be incomplete (28,29) and they may actually perpetuate cultural myths and misinformation (30,31). In HICs such as Australia and the UK, menstruation may be taught at school as part of sex education, if it is mentioned at all, but many girls do not get the practical information that they need (32,33). In both HIC and LMIC non-government agencies have stepped in to try to plug the gap by providing Menstrual Education Interventions.

In this review our objective was to describe and evaluate the impact of menstrual education interventions intended to equip young adolescent girls with the knowledge and skills to promote Menstrual Health.

Previous reviews have focussed on the more narrow 'Menstrual Hygiene Management' in LMIC (34,35). The term Menstrual Health is now preferred to hygiene, partly to avoid the suggestion that menstruation *per se* is unclean, but mostly to emphasize the holistic nature of the menstrual experience. This literature review only includes publications since 2014 in order to reflect that change.

METHODS

This is a systematic review of published literature of interventions that include an element of menstrual education delivered to young adolescents and inexperienced menstruators. We report according to the Preferred Reporting Items for Systematic Reviews and Meta-Analyses (PRISMA) guidelines

Patient and Public Involvement

No patient involvement

Publication Date and Language

This review draws upon papers published from January 2014 until May 2020 to bring the field up to date. Only reviews published in the English language have been included.

Participants

For this review we were interested in interventions targeted at young adolescents aged 10 -14 years old.

Inclusion and Exclusion Criteria

Studies of interventions which had been evaluated were sought.

All interventions which had a component of menstrual information transfer were included if the intervention sought to :

For peer review only - http://bmjopen.bmj.com/site/about/guidelines.xhtml

[Type here]

- Increase knowledge of menstruation to reduce anxiety and shame, and normalise the experience
  Increase skills and competencies to manage menstruation comfortably and hygienically
- Increase awareness of strategies for self-care of menstrual symptoms

Interventions that were straightforward ‘Menstrual Education’ in which lessons about puberty, anatomy, and hygiene were delivered by teachers or other educators were eligible. Interventions focussed on skills training, such as correct menstrual cup insertions, and delivered by nurses or other key workers were also eligible. Programmes that facilitated peer or self-guided learning through the provision of resources or spaces (both physical and remote) for learning to occur were also eligible for inclusion.

Studies were excluded if the improvements were in hardware such as toilets or pads without any accompanying education or training, or if they provided hygiene education without reference to menstruation specifically. Studies were excluded if they were about abnormal menstruation, menstrual problems as a co-morbidity, or if the research was investigating endocrinology or non-human models. Studies that described existing Knowledge, Attitudes and Practice without any intervention were also excluded.

Studies that included adolescents up to the age of 19 were not excluded if the aim were to instruct inexperienced menstruators. Some studies included older girls because they were intellectually disabled and were part of the intervention based on intellectual age rather than chronological age. Some studies included older girls because they were members of classes assigned by grade rather than age. But studies about interventions aimed at adult women were excluded.

Study design and quality were not part of the criteria in order to capture as broad an interpretation of menstrual education as possible.

Protocol for Identification of Academic Literature

Screening

As the field of Menstrual Health Education is highly interdisciplinary, we searched key medical and social science databases: ASSIA Applied Social Science Index and Abstracts; CINAHL Cumulative Index to Nursing and Allied Health Literature; EMBASE Excerpta Medica database; MEDLINE Medical Literature Analysis and Retrieval System Online; Sociological Abstracts; Web of Science; IBBS International Bibliography of the Social Sciences; TRoPHI Trials Register of Promoting Health Interventions.

The search parameters combined the target population, menstruation, education and programme.

Table 1. Search Terms Used

**Search Terms Used**
Search term 1<br>‘adolescen* OR girl* OR teenage* OR youth* OR young OR pre-adolescen* OR school-girl OR Out-of-School-Youth OR OOSY OR female OR woman<br>AND Search term 2<br>Menstrua* OR menarche* OR mense* OR catamenia OR menarche* OR menstrual health OR menstrual hygiene OR menstrual management OR sanitation OR menstrual etiquette

[Type here]

For peer review only - http://bmjopen.bmj.com/site/about/guidelines.xhtml

[Type here]

AND Search term 3<br>know* OR understand*OR manage* OR learn* OR apprehen* OR comprehensi* OR educat* OR aware* OR familiar* OR proficien*<br>AND Search term 4<br>AND arrangement* OR evaluat* OR initiative* OR intervention*OR model* OR package* OR pilot* OR program* OR project* OR provision* OR regime* OR scheme* OR strateg* OR trial* OR approach* OR polic*		
**Database Returns**		
**Database**	**Search Terms and filters**	**Returns**
ASSIA (proquest)	1 (ab), 2 (ti), 3 (ab), 4 (ab)<br>01/01/14 to 01/09/19	15
CINAHL	1 (ab), 2 (ti), 3 (ab), 4 (ab)<br>01/01/14 to 01/09/19	126
EMBASE<br>Including MEDLINE	1 (ab), 2 (ti), 3 (ab), 4 (ab)<br>2014 -2019<br>English, Female	732
Sociological abstracts (proquest)	1 (ab), 2 (ti), 3 (ab), 4 (ab)<br>01/01/14 to 01/09/19	14
Web of Science	TS=(1), (3), (4) AND Ti=(2)<br>Last 5 years	323
IBBS (proquest)	1 (ab), 2 (ti), 3 (ab), 4 (ab)<br>01/01/14 to 01/09/19	26
TRoPHI (Eppi centre, google)	No wild cards<br>Title<br>Menstruation and Knowledge<br>2014-2019	4
**TOTAL SAVED to EXCEL**		**1240**

Two reviewers (RE and CO) screened abstracts and full texts of all citations obtained for eligibility independently. Data extraction of eligible material and the quality assessment was conducted by RE using a data extraction framework agreed upon by FG, BH and RE.

Outputs and Data Extraction

The outputs of Menstrual Education are changes to Menstrual Knowledge, Attitudes and Practices (KAP). In quantitative studies, Pre- and Post-Intervention KAP scores were collected, as well as data to support the validity of the scores, such as sample frame and number of participants. Other descriptive measures used in qualitative studies and contextual factors that might have a bearing on the outputs were also recorded where given.

Quality Assessment

We used the Mixed Methods Appraisal Tool (MMAT) for quality assessment because it is suitable for a variety of study designs. The MMAT components focus on the clarity of the research questions and the appropriateness of the data collection methods. Our intention was to consider the MMAT assessment in the interpretation of study findings.

Analysis and Synthesis

Quantitative analysis

[Type here]

For peer review only

The results of quantitative studies were tabulated for comparison. Not all data could be converted but for studies that reported quantitative results with a pre- and post- test score of Menstrual Knowledge, we found the effect size by calculating the Standard Mean Difference using Cohen's d. Cohen's d is calculated by the formula: the difference between the two means divided by the pooled standard deviation. The test statistic could be between 0 and infinity. Cohen suggested that a small effect size is a value of 0.2, a medium one is 0.5 and a large one is 0.8. Some social science disciplines report much larger sizes and the statistical guidance was revised to suggest that a medium effect is the average of those in the relevant literature (36,37). However, Cohen's d has not previously been calculated for this discipline and therefore the magnitude of the effect size can only be considered relative to others in this review.

Qualitative analysis

A Qualitative Comparative Analysis (QCA) (38) was made of all studies by scrutinising the data extracted for common themes and reporting them in a narrative summary.

Synthesis: Building A Logic Model

Logic models enable the key findings of the analysis to be synthesized into a theory of change. We contructed a logic model to frame the effect of Menstrual Education Interventions on Menstrual Health.

Guided by logic models developed for school-based interventions (39), we worked backwards from the higher order aim of good Menstrual Health to propose a chain of causal events.

The aim of good Menstrual Health is the distal outcome to the intervention. It is characterized by possessing menstrual positivity. Girls can manage their menstrual cycle confidently and feel empowered to make choices about their own bodies and lives. They engage with health services and they can fully participate in school.

The distal outcome requires an improvement in the preceding intermediate outcome. The intermediate outcome is Quality of Life (not restricted by menstruation): mobility, participation in normal activities (eating/drinking with the family) and school attendance.

Below that is the proximal outcome; hygienic and comfortable menstruation management, which requires access to menstrual products, WASH facilities, painkillers, and the use of self-care practices to manage the symptoms.

Menstrual KAP underpins these outcomes. In a theory of change, girls may require knowledge of the menstrual cycle in order to prepare products for menstruation and to practice self-care. They may need confidence to ask for products and services. They may need skills to use the products correctly to avoid the risk of discomfort, leaks or of contracting reproductive tract infections.

Inputs and Outputs to the Logic Model

Menstrual Education and training are the inputs . The output is improved menstrual knowledge, attitudes and practices (KAP). Outputs are linked to outcomes.

LOGMOD

Figure 1. Proximal, intermediate, distal and macro-distal outcomes and indicators of a menstrual health intervention. Menstrual Education and Training Inputs act on Girls' Menstrual Knowledge, Attitude and Practices, and Outputs are measured by increasing KAP score. Positive feedback

For peer review only - http://bmjopen.bmj.com/site/about/guidelines.xhtml

[Type here]

operates as girls gain knowledge and skills they become more confident to seek further knowledge and skills.

Indicators

The indicators of proximal outcomes (that girls can manage their menstruation hygienically and comfortably) are access to Menstrual products, WASH facilities and painkillers, and the use of self-care practices to manage their symptoms. Girls need to have access to sufficient suitable clean products, private facilities to change, wash and dispose of used products, painkillers to manage dysmenorrhea and the means to practice self-care, such as yoga or taking a rest.

The indicators of intermediate outcomes (quality of life) are unrestricted mobility, participation in normal activities and school attendance. Not only do girls need the means to manage their menstruation hygienically, they need to have self-confidence in their ability to manage outside of the confines of the house, and assurance to partake in activities from which they may be traditionally excluded.

The indicators of distal outcomes (menstrual health) are school attainment, product choice, menstrual tracking and engagement with Health services. School attainment is an indicator of unimpeded participation, not just attendance at school. This means that girls are able to concentrate on their schooling and not on their worries surrounding menstruation. Girls that have agency are able to control their menstruation and not the other way around. They can choose a suitable menstrual product to meet their individual needs. They track their menstrual cycle to be well-prepared so that they are not caught out and have to go home, and they engage as necessary with reproductive health services, without shame or stigma.

The indicators of macro-level distal outcomes, which is an increase in the menstrual literacy of the society, are a lifting of menstrual restrictions and gender-equal educational opportunities. These may include improved reproductive health indicators such as reduced RTIs and reduced pregnancy rates.

RESULTS

A total of 1240 papers were recovered using the search terms. 900 remained after the removal of duplicates and 48 were saved to Excel for full text screening.

PRIFLO

Figure 2. PRISMA Flow-chart

Study Characteristics

Twenty-four papers met the inclusion criteria: They were grouped into the following categories of study design according to the MMAT Quality Appraisal tool: 12 Randomised Controlled Trials (RCTs); 5 quantitative descriptive studies; 1 quantitative non-random study; 5 mixed-methods studies and 1 qualitative study. *See Supplementary Files for further detail on study design.*

There were four pairs of papers, each reporting on different aspects of the same trial (19,40); (41,42); (10,20) and (31,43).

For peer review only - http://bmjopen.bmj.com/site/about/guidelines.xhtml

[Type here]

The study dates ranged from 2012 to 2017, with dates of publication ranging from 2014 to 2019. Studies were undertaken in Iran (4), Turkey, Indonesia (2), Ethiopia, India (4), Bangladesh, Uganda (2), USA, Nepal, Kenya and China.

Of the studies, two included boys (10,20,44); one included mothers (29,31) and two focussed on intellectually-disabled adolescent girls (45,46). The number of study participants varied from 1 to 2564. The total number of participants in the 20 different intervention studies was 10362.

The amount of time after the completion of the intervention to the assessment varied from the same day to up to 5 years, with 13 of the studies in the range of 1 – 9 months, and the mode being 1 month. A common theme was allowing one menstrual cycle to pass before re-testing.

Delivery

Eight of the interventions used health professionals as instructors (6,9,10,31,40,45,47–49). Five were researcher-led (31,43,46,50,51); five were led by teachers (20,52–55); two used peer-educators (9,48) and the others did not make it clear. A wide range of intensity and duration models were used, ranging from three 5-minute videos in the space of one afternoon (44) to 1 hr per day for four months (47). Only two embedded menstrual education into the school curriculum (54,55).

Quality Assessment

The methodological quality of study designs was mixed: 11 were rated as high quality and 13 as moderate to low. Those considered to be of the highest quality were randomized controlled trials which included comparison groups (six studies). The research questions were clear and the data collection methods appropriate. Of the other studies, several methodological limitations were noted; commonly, neither the delivery team nor the participants were blinded (9); adequate randomization of the participants was lacking (10) and /or relevant confounds were not identified or controlled(4). The quality of data analysis also varied considerably, with the weakest having small sample sizes and no measure of statistical significance (2).

Interventions

There was a range of intervention types. Eight of the interventions employed traditional education in the form of didactic teaching, sometimes supplemented with posters, flip-charts, and question-and-answer sessions (19,40,47,50,51,54–56). One intervention employed a more formal lecture presentation and gave out some supporting literature (29,31). Two interventions used stories and video presentations (44,46) and two interventions distributed puberty books (4,52) without further teacher input. Two interventions facilitated learning through peer-education (9,48) and five different interventions focussed on Menstrual Hygiene Management training: some demonstrated pad usage with a menstrual kit (10,20,46,53) or using a doll (45) and one instructed participants on the use of menstrual cups (42,57)

Table 2 Characteristics of interventions

Author	Lecture or presenta-tion	Teaching with Q and A or discussion	Stories or Videos	Puberty Books or pamplet	Peer-education	Demonstra-tion or Simulation

[Type here]

For peer review only

[Type here]

		and visual aids				
(Afsari *et al.*, 2015)	√			√		
(Altundağ and Çalbayram, 2016)						√
(Arasteh *et al.*, 2019)		√			√	
(Ariyanti and Royanto, 2018)			√			√
(Blake *et al.*, 2018)				√		
(Rani Chadalawada, Devi S and Rani M, 2017)	√					√
(Haque *et al.*, 2014a)	√			√		√
(Hennegan and Montgomery, 2016b)		√	√			
(Hurwitz *et al.*, 2018)			√			
(Jarrahi, Golmakani and Mazlom, 2020)		√			√	
(Kansiime *et al.*, 2020)		√	√			√
(Kheirollahi *et al.*, 2017)		√				
(Mokari, Khaleghparast and Samani, 2016)		√				
(Montgomery *et al.*, 2016)		√	√			
(Morrison *et al.*, 2018)	√					
(Nalugya *et al.*, 2020)		√	√			√

[Type here]

For peer review only - http://bmjopen.bmj.com/site/about/guidelines.xhtml

(Penelope A. Phillips-Howard *et al.*, 2016)	√					√
(Ramaiya *et al.*, 2019)		√	√	√	√	
(Setyowati, Rizkia and Ungsianik, 2019)				√		
(Sharma *et al.*, 2015)	√					
(Sivakami *et al.*, 2019)	√	√				
(Su and Lindell, 2016)		√	√	√	√	√
(Valizadeh *et al.*, 2017)	√			√		
(Van Eijk *et al.*, 2016)	√					√

For peer review only

Aims of the Studies

The common aim of the studies was to evaluate the impact of a Menstrual Education Intervention on Menstrual Health for adolescent girls. A number of studies were more broadly about puberty education (31,43,44,50,52,54) Several studies measured Menstrual Knowledge, Attitudes and Practices (KAP) (4,6,59,31,44,47,48,51,52,56,58). Nearly all of the studies used a pre-test, post-test model but one study used a post-test only model (44) A small number specifically focussed on Menstrual Hygiene Management and evaluated training on pad replacement or cup insertion (10,20,35,40,42,45,46,48,57)

Analysis

The quantitative and qualitative results are reported under four main themes: Menstrual Knowledge, Menstrual Attitudes, Menstrual Practices and Multi-component interventions.

Quantitative results

Menstrual Knowledge. A meta-analysis was conducted on 11 studies which measured an improvement in menstrual knowledge following an intervention. All studies reported that their results showed a significant improvement in menstrual knowledge. Where studies reported the mean and standard deviation of a menstrual knowledge questionaire, we calculated the effect size (Cohen’s d).

[Type here]

The effect size of those that distributed pamphlets and books was lowest at 0.33 (31), followed by those that showed videos 1.40 (44) and then lectures with question and answer sessions 2.13 (51) and 4.81 (56). Small group or peer teaching was very high 5.337 and 10.044 respectively (48)

For peer review only

[Type here]

[Type here]

Table 3 Effect size of interventions designed to improve menstrual knowledge

Study type and measure	First author and date of publication	Intervention	Number of questions / N	Reliability/ Cronbach's alpha (except where stated otherwise)	Sample size / n	Treatment	Mean / arbitary units (except where stated otherwise	Standard Deviation / Arbitary units (except where stated otherwise)	Number of individual'sachieving a	Statistical test / p value	Effect size / Cohen's d (except where stated otherwise)	impact of intervention
**control/intervention pre-test, post test**	Blake et al 2018	Distribution of puberty education books in the local language	9	0.77	318	control	(Mean Difference = 0.18)	(Pooled SD = 1.4)		Wald Chi-squared, <0.001	**0.792**	medium
					318	intervention	(MD = 1.06)	(Pooled SD = 1.52)				
	Jarrahi et al 2020	Small group and peer teaching	34	0.78	30	control pre	45.1	8.4		Kruskal-Wallis test, <0.001	**5.337 between control and small group, 10.044 between control and peer**	very high
					30	control post	52.2	1				
					30	intervention 1 pre (small group)	48.3	6.1				
					30	intervention 1 post	84.5	8.5				
					30	intervention 2 pre (peer)	44.1	1.7				
					30	intervention 2 post	93.3	5.7				

[Type here]

For peer review only - http://bmjopen.bmj.com/site/about/guidelines.xhtml

[Type here]

	Setyowati et al 2019	Booklet	14	0.886	87	control pre			no data	Chi-squared, <0.001	-32.20%	high
					87	control post			17.2			
					87	intervention pre			58.6			
					87	intervention post			90.8			
	Sharma et al 2015	Teaching programme led by School Nurses, focussing on hygiene practices	15	test, retest r = 0.93	25	control pre	8.02	no data		t-test, <0.001	**4.48**	very high
					25	control post	8.06					
					25	intervention pre	8.04					
					25	intervention post	12.6					
	Su et al 2016	Lecture, question and answer	13	KR20= 0.64	56	control pre	5.5	2.54		t-test, <0.001	**2.13**	high
					56	control post	5.71	2.3				
					60	intervention pre	5.73	2.56				
					60	intervention post	10.22	1.92				
	Valizadeh et al 2017	Lecture, booklet and	15	0.72	120	control pre	8.5	2.5		general linear	**0.33 between**	low to medium

For peer review only

[Type here]

		pamphlets			120	control post	9.1	2.4		model, <0.002	**groups, 0.8 within groups**	
					124	intervention pre	8.2	2				
					124	intervention post	9.8	1.8				
	Kheirollahi et al 2017	Lectures, questions and answers focussing on normal and abnormal signs of puberty	23 (100 point scale)	0.8	76	control pre	52.58	6.58		t-test, <0.001	**4.81**	very high
					76	control post	52.77	6.87				
					76	intervention pre	55.83	6.77				
					76	intervention post	86.36	7.11				
**control/intervention post-test only**	Hurwitz et 2017	Health education videos, using animation	27	0.72	40	control	11.27	3.73		t-test, <0.001	**1.403**	high
					40	intervention	15.67	2.4				
**one group pre-test, post test**	Arasteh et al 2019	Group counselling and peer education	15	0.8	30	pre	6.8	3.32		Chi-squared, <0.001	**1.64**	high
					30	post	11.3	2				
	Haque et al 2014	Training with Field manual by medical professionals	10	0.73	416	pre			51	chi-squared, <0.05	-31.40%	high
					416	post			82.4			
	Chadawada et al 2017	Teaching with posters, flip-charts and videos	4	no data	250	pre			72.7	chi-squared, <0.05	-13.90%	medium
					250	post			86.6			

For peer review only

[Type here]

Menstrual Attitudes. Five studies measured Menstrual Attitudes (4,31,43,44,51,53).

Four interventions reported significantly different attitude scores, and three of those provided pamphlets that addressed cultural restrictions(4,43,59). An intervention on dysmenorrhea and self-care included pamphlets with video and peer-sharing and girls who had taken part had a 'significant increase in confidence and decrease in bothersome menstrual attitude' (51). The only intervention which did not find a significant difference in attitude pre- and post-test involved puberty education videos shown to early adolescent boys and girls (44) and maybe did not resonate so much.

Menstrual Practice. An intervention that trained intellectually-disabled adolescents in an 18-item pad replacement skill set found that pre-training and post-training differences were statistically significant (45). A feasibility trial into the use of the menstrual cup by school girls in Kenya (49) found that usage increased as time went on and culminating in 96% usage after 9 months. There was also an increase in hygiene, with the menstrual cup reported as reducing the prevalence of STIs from 19.2% to 12.9% (41).

Multi-component interventions

Interventions that are multi-component may be more successful than those that are single-component. An education intervention in India was part of a bigger project that involved pad provision and improved sanitation in schools. After four years, compared to unimproved schools, school attrition had fallen from 11% to 6% (55). The effect of menstrual education alone cannot be separated out.

Qualitative results

Menstrual Knowledge. All studies reported an increase in Menstrual Knowledge. Interventions that used peer education and group counselling (9,48) were just as effective as those delivered by medical professionals (53).

Those interventions that had a degree of interactivity were more effective than those that only gave out information. Those that encouraged discussions found that they led to an increased willingness to talk about menstruation and a greater awareness of what is normal (9,50).

Whilst schools would seem to be the natural place to deliver menstrual education, only two interventions embedded it into the school curriculum (54,55). In Nepal, some schools had received the WASH in Schools programme (WinS) (60). However, the girls were highly critical of their teachers, especially male teachers. They complained that 'Teachers often got embarrassed, referred students to their textbook, and did not answer questions' (54).

Menstrual Attitudes. Most studies commented on an improved menstrual attitude and one noted a reduction in anxiety (50). More than one study noted an improvement in confidence in performing menstrual health care behaviour, such as requesting pain relief for dysmenorrhea (51). Some studies observed an increased confidence of girls to push back against cultural restrictions, or harmful practices (9,19,50,53,61).

Menstrual Practices. Skills are required to use pads and cups so that they are comfortable and don't leak. Cups in particular may be difficult to position. The cup feasibility trial in Kenya found that on-going training and support may be required to master the technique over a period of 6 months to a

[Type here]

For peer review only - http://bmjopen.bmj.com/site/about/guidelines.xhtml

year (49,57). Education was also found to be an important component of skill acquisition in Uganda, where pad-provision accompanied by education was shown to be more effective than pad-provision alone (19).

Multi-component interventions

The MENISCUS intervention in Uganda attributed its success to the synergy of five combined elements; teacher training on puberty education, a drama skit, pad provision, pain relief provision, and Water, Sanitation and Hygiene (WASH) facility improvements (10).

Synthesis: The Logic Model

The results provide evidence that menstrual education improves the menstrual knowledge, attitudes and practices (KAP) of young adolescent girls. It is suggested that increasing the menstrual KAP of girls increases their confidence to seek further knowledge and skills in a positive feedback loop (see fig 2). Menstrual Education is viewed as underpinning the logic model and is the first step to achieving Menstrual Health.

Some of the menstrual education studies were part of bigger trials and have reported indicators relevant to the proximal, intermediate or distal outcomes of the logic model.

DISCUSSION

All twenty-four included studies reported a positive outcome; that there was an improvement in either Menstrual Knowledge, Attitudes or Practice which led to improvement in hygienic menstruation management, the use of self-care practices, or access to products and facilities. There was limited evidence for change in school attendance, mobility and refusal to accept restrictions. These are intermediate outcomes and it might be expected to take some time to move from the proximal to the intermediate outcomes in the theory of change.

A larger effect was gained with the more interactive interventions. We suggest that this relates to the higher degree of participation, and concurs with current educational philosophy about the importance of active learning (62), based on constructivist theory (63,64). Gardner added to Dewey's work on active learning with 'transformative' teaching, using a range of methods that encourage the learner to find their own entry point and engage with the subject, often utilising space and creativity, and linking with their own experience.(65). Discussing menstruation gives the girls agency to determine what it is that they need to know for themselves and the confidence to demand access to an enabling infrastructure.

Most of the interventions were delivered in the school setting where it is relatively easy to reach the target group, although only two embedded the education into the school curriculum. In theory, schools should have good coverage and objectivity for delivery of this sensitive information at an appropriate time. However, teachers themselves may be ill-equipped to teach about menstruation without proper training (66).

A logic model has been constructed to frame the effect of menstrual education interventions on Menstrual Health. Menstrual Education is seen as underpinning all desirable outcomes and programmes can be optimised by including an education aspect. Multi-component interventions

For peer review only - http://bmjopen.bmj.com/site/about/guidelines.xhtml

For peer review only

[Type here]

may be more successful than single component. From a constructivist perspective which places learning within a social context(64,67) interventions that seek to improve the menstrual literacy of the whole community and reduce menstrual stigma may be more effective in achieving the more distal outcomes of menstrual health and girl empowerment.

Implications for Policy and Practice

This review provides evidence that menstrual education has a positive effect on the menstrual knowledge, attitudes and practices of adolescent girls and it would be prudent to ensure that menstrual education is embedded into school curricula and delivered by trained personnel who can facilitate discussion. However, without an enabling infrastructure, the progress towards menstrual health will be limited. In order to achieve the more distal outcomes of the logic model, programme and policy makers need to address the menstrual literacy of the wider population.

Limitations of this Review

The review was carried out in the English language, which may have missed some publications. Because menstrual health is an emerging topic with evolving terminology, search terms may not have adequately captured all currently used descriptors.

As a mixed methods review, there are a number of systemic limitations derived from comparing heterogeneous data sets. In particular, the studies did not measure the same outputs and the methodological quality of the studies was mixed.

Although all interventions reported positive outcomes, this may be due to publication bias, where only significant results are shared.

REGISTRATION

For this review, a protocol was not prepared or registered.

REFERENCES

1. Chandra-Mouli V, Patel S V. Mapping the knowledge and understanding of menarche, menstrual hygiene and menstrual health among adolescent girls in low- and middle-income countries. Reprod Health. 2017;14.

2. Marván ML, Alcalá-Herrera V. Age at Menarche, Reactions to Menarche and Attitudes towards Menstruation among Mexican Adolescent Girls. J Pediatr Adolesc Gynecol. 2014;27(2):61–6.

3. Mason L, Nyothach E, Alexander K, Odhiambo FO, Eleveld A, Vulule J, et al. “We keep it secret so no one should know” - A qualitative study to explore young schoolgirls attitudes and experiences with menstruation in rural Western Kenya. PLoS One. 2013;8(11).

4. Setyowati S, Rizkia M, Ungsianik T. Improving Female Adolescents’ Knowledge, Emotional Response, and Attitude toward Menarche following Implementation of Menarcheal

[Type here]

For peer review only

[Type here]

Preparation Reproductive Health Education. Asian/Pacific Isl Nurs J. 2019;4(2):84–91.

5. Shah V, Nabwera HM, Sosseh F, Jallow Y, Comma E, Keita O. A rite of passage : a mixed methodology study about knowledge , perceptions and practices of menstrual hygiene management in rural Gambia. BMC Public Health. 2019;19(277):1–15.

6. Sharma R, Sandhya N, Deepika K, Varsha S, Vardha. Menstrual hygiene among adolescent girls. Indian J Community Heal. 2015;27(3):376–80.

7. Marván ML, Alcalá-Herrera V. Age at menarche, reactions to menarche and attitudes towards menstruation among Mexican adolescent girls. J Pediatr Adolesc Gynecol. 2014;27(2):61–6.

8. Coast E, Lattof SR, Strong J. Puberty and menstruation knowledge among young adolescents in low- and middle-income countries: a scoping review. Vol. 64, International Journal of Public Health. Springer International Publishing; 2019. p. 293–304.

9. Ramaiya A, Malhotra A, Cronin C, Stevens S, Kostizak K, Sharma A, et al. How does a Social and Behavioral Change Communication Intervention Predict Menstrual Health and Hygiene Management: A Cross-Sectional Study. BMC Public Health. 2019;19(1):1–12.

10. Nalugya R, Tanton C, Hytti L, Kansiime C, Nakuya K, Namirembe P, et al. Assessing the effectiveness of a comprehensive menstrual health intervention program in Ugandan schools (MENISCUS): process evaluation of a pilot intervention study. Pilot Feasibility Stud. 2020;6(1):1–15.

11. House S, Mahon T, Cavill S. Menstrual Hygiene Matters: a resource for improving menstrual hygiene around the world. Reprod Health Matters. 2013 Jan;21(41):257–9.

12. Hennegan JM. Menstrual Hygiene Management and Human Rights: The Case for an Evidence-Based Approach. Women's Reprod Heal. 2017;

13. Ellis A, Haver J, Villasenor J, Parawan A, Venkatesh M, Freeman MC, et al. WASH challenges to girls' menstrual hygiene management in Metro Manila, Masbate, and South Central Mindanao, Philippines. Waterlines. 2016;

14. Rani A, Sharma MK, Singh A. Practices and perceptions of adolescent girls regarding the impact of dysmenorrhea on their routine life: A comparative study in the urban, rural, and slum areas of Chandigarh. Int J Adolesc Med Health. 2016;28(1):3–9.

15. Sumpter C, Torondel B. A Systematic Review of the Health and Social Effects of Menstrual Hygiene Management. PLoS One. 2013;8(4).

16. Tiwari A, Ekka IJ, Thakur R. Assessment of knowledge and practices regarding menstrual hygiene among adolescent girls of Government higher secondary school, station Murhipar, Rajnandgaon (C.G.). Int J Community Med Public Heal. 2018;5(4):1335–8.

17. Sommer M, Kjellén M, Pensulo C. Girls' and women's unmet needs for menstrual hygiene management (MHM): The interactions between MHM and sanitation systems in low-income countries. Vol. 3, Journal of Water Sanitation and Hygiene for Development. 2013. p. 283–97.

18. Hennegan J. Inserting informed choice into global menstrual product use and provision. Lancet Public Heal. 2019;4(8):e361–2.

19. Hennegan J, Dolan C, Steinfield L, Montgomery P. A qualitative understanding of the effects of reusable sanitary pads and puberty education: implications for future research and practice. Reprod Health. 2017;14.

20. Kansiime C, Hytti L, Nalugya R, Nakuya K, Namirembe P, Nakalema S, et al. Menstrual health

[Type here]

For peer review only - http://bmjopen.bmj.com/site/about/guidelines.xhtml

[Type here]

intervention and school attendance in Uganda (MENISCUS-2): A pilot intervention study. BMJ Open. 2020;10(2):1–11.

21. Sebert Kuhlmann A, Peters Bergquist E, Danjoint D, Wall LL. Unmet Menstrual Hygiene Needs Among Low-Income Women. Obstet Gynecol. 2019;133(2):238–44.

22. PHS. Lifting the lid on period poverty. 2019.

23. Whitehead S, Biddle S. Adolescent girls' perceptions of physical activity: A focus group study. Eur Phys Educ Rev. 2008;14(2):243–62.

24. Biddle SJH, Whitehead SH, O'Donovan TM, Nevill ME. Correlates of participation in physical activity for adolescent girls: A systematic review of recent literature. J Phys Act Heal. 2005;2(4):423–34.

25. Gultie T, Hailu D, Workineh Y. Age of Menarche and Knowledge about Menstrual Hygiene Management among Adolescent School Girls in Amhara Province, Ethiopia: Implication to Health Care Workers & School Teachers. PLoS One. 2014;9(9).

26. Al Omari O, Razeq NMA, Fooladi MM. Experience of Menarche Among Jordanian Adolescent Girls: An Interpretive Phenomenological Analysis. J Pediatr Adolesc Gynecol. 2016;29(3):246–51.

27. Raskova M, Stolinska DP. COMMUNICATION ABOUT PUBERTY AMONG MIDDLE-SCHOOL-AGED CHILDREN. In: Bekirogullari Z, Minas MY, Thambusamy RX, editors. International Conference on Education and Educational Psychology. 2017. p. 194–202.

28. Bennett C, Harden J. An exploration of mothers' attitudes towards their daughters' menarche. Sex Educ Soc Learn. 2014;14(4):457–70.

29. Afsari A. Predictors of knowledge of puberty health among girl students. Biosci Biotechnol Res Commun. 2017;10(2):264–9.

30. Sooki Z, Shariati M, Chaman R, Khosravi A, Effatpanah M, Keramat A. The Role of Mother in Informing Girls About Puberty: A Meta-Analysis Study. Nurs Midwifery Stud. 2016;Inpress(Inpress):1–10.

31. Valizadeh S, Assdollahi M, Mirghafourvand M, Afsari A. Educating Mothers and Girls about Knowledge and Practices toward Puberty Hygiene in Tabriz, Iran: A Randomized Controlled Clinical Trial. Iran Red Crescent Med J. 2017;19(2).

32. Tingle, C & Vora S. Break the Barriers : Girls ' Experiences of. Plan Int UK. 2018;1–68.

33. Collier-Harris CA, Goldman JDG. Is puberty education evident in Australia's first national curriculum? Sex Educ Soc Learn. 2017;17(1):57–72.

34. Sumpter C, Torondel B. A Systematic Review of the Health and Social Effects of Menstrual Hygiene Management. PLoS One. 2013;8(4).

35. Hennegan J, Montgomery P. Do menstrual hygiene management interventions improve education and psychosocial outcomes for women and girls in low and middle income countries? A systematic review. PLoS One. 2016;11(2):1–21.

36. Gignac GE, Szodorai ET. Effect size guidelines for individual differences researchers. Pers Individ Dif. 2016;102(December):74–8.

37. Hemphill JF. Interpreting the Magnitudes of Correlation Coefficients. Am Psychol. 2003;58(1):78–9.

[Type here]

For peer review only - http://bmjopen.bmj.com/site/about/guidelines.xhtml

38. Thomas J, Harden A. Methods for the thematic synthesis of qualitative research in systematic reviews. BMC Med Res Methodol. 2008;8(45):1–10.

39. Harris K, Kneale D, Tj L, Vm M, Grigg J, Thomas J, et al. School-based self-management interventions for asthma in children and adolescents : a mixed methods systematic review (Review). 2019;

40. Montgomery P, Hennegan J, Dolan C, Wu M, Steinfield L, Scott L. Menstruation and the Cycle of Poverty: A Cluster Quasi-Randomised Control Trial of Sanitary Pad and Puberty Education Provision in Uganda. PLoS One. 2016;11(12).

41. Phillips-Howard PA, Nyothach E, Ter Kuile FO, Omoto J, Wang D, Zeh C, et al. Menstrual cups and sanitary pads to reduce school attrition, and sexually transmitted and reproductive tract infections: A cluster randomised controlled feasibility study in rural Western Kenya. BMJ Open. 2016;

42. Van Eijk AM, Sivakami M, Thakkar MB, Bauman A, Laserson KF, Coates S, et al. Menstrual hygiene management among adolescent girls in India: A Systematic review and meta-analysis. BMJ Open. 2016;6(3).

43. Afsari A, Mirghafourvand M, Valizadeh S, Abbasnezhadeh M, Galshi M, Fatahi S. The effects of educating mothers and girls on the girls' attitudes toward puberty health: A randomized controlled trial. Int J Adolesc Med Health. 2015;2015.

44. Hurwitz LB, Lovato SB, Lauricella AR, Woodruff TK, Patrick E, Wartella E. "A New You, That's Who": an evaluation of short videos on puberty and human reproduction. Palgrave Commun. 2018;4(1).

45. Altundağ S, Çalbayram NÇ. Teaching menstrual care skills to intellectually disabled female students. Mol Ecol. 2016;25(13–14):1962–8.

46. Ariyanti TD, Royanto LRM. The Effectiveness of Social Stories and Video Modeling in Improving Self-Care Skills in Female Adolescents with Mild Intellectual Disabilities during Menstrual Periods. 2018;135(Iciap 2017):189–99.

47. Rani Chadalawada U, Devi S A, Rani M S. Effect of Health Education on Adolescent Girls Regarding Knowledge About Menstruation. J Evol Med Dent Sci. 2017;6(13):1040–3.

48. Jarrahi R, Golmakani N, Mazlom SR. Effect of menstrual hygiene education based on peer and small group teaching methods on hygiene behaviors in female adolescents: A comparative study. Evid Based Care J. 2020;10(1):70–4.

49. Van Eijk AM, Laserson KF, Nyothach E, Oruko K, Omoto J, Mason L, et al. Use of menstrual cups among school girls: Longitudinal observations nested in a randomised controlled feasibility study in rural western Kenya. Reprod Health. 2018;15(1):1–11.

50. Mokari H, Khaleghparast S, Samani LN. Impact of Puberty Health Education on Anxiety of Adolescents. Int J Med Res Heal Sci. 2016;5(5, S, SI):284–91.

51. Su JJ, Lindell D. Promoting the menstrual health of adolescent girls in China. Nurs Heal Sci. 2016;18(4):481–7.

52. Blake S, Boone M, Yenew Kassa A, Sommer M. Teaching Girls About Puberty and Menstrual Hygiene Management in Rural Ethiopia: Findings From a Pilot Evaluation. J Adolesc Res. 2018;33(5):623–46.

53. Haque SE, Rahman M, Itsuko K, Mutahara M, Sakisaka K. The effect of a school-based educational intervention on menstrual health: An intervention study among adolescent girls

For peer review only - http://bmjopen.bmj.com/site/about/guidelines.xhtml

[Type here]

in Bangladesh. BMJ Open. 2014;4(7):1–9.

54. Morrison J, Basnet M, Bhatt A, Khimbanjar S, Chaulagain S, Sah N, et al. Girls' menstrual management in five districts of Nepal: Implications for policy and practice. Stud Soc Justice. 2018;12(2):251–72.

55. Sivakami M, van Eijk AM, Thakur H, Kakade N, Patil C, Shinde S, et al. Effect of menstruation on girls and their schooling, and facilitators of menstrual hygiene management in schools: Surveys in government schools in three states in India, 2015. J Glob Health. 2019;9(1).

56. Kheirollahi F, Rahimi Z, Arsang-Jang S, Sharifirad G, Sarraf P, Gharlipour Z. Puberty health status among adolescent girls: A model- based educational program. Int J Pediatr. 2017;5(7):5369–78.

57. Phillips-Howard PA, Nyothach E, Ter Kuile FO, Omoto J, Wang D, Zeh C, et al. Menstrual cups and sanitary pads to reduce school attrition, and sexually transmitted and reproductive tract infections: A cluster randomised controlled feasibility study in rural Western Kenya. BMJ Open. 2016;6(11):1–11.

58. Arasteh FE, Shobeiri F, Parsa P, Mohamadi Y. THE EFFECT OF COUNSELING ON KNOWLEDGE, ATTITUDE AND PRACTICE OF PUBERTY HEALTH AMONG ADOLESCENT GIRLS LIVING IN WELFARE BOARDING CENTERS. Indo Am J Pharm Sci. 2018;5(1):11–6.

59. Haque SE, Rahman M, Itsuko K, Mutahara M, Sakisaka K. The effect of a school-based educational intervention on menstrual health: An intervention study among adolescent girls in Bangladesh. BMJ Open. 2014;4(7):1–9.

60. United Nations Children's Fund (UNICEF) and World Health Organization 2018. Drinking Water, Sanitation and Hygiene in Schools : Global baseline report 2018. Unicef. 2018. 82 p.

61. Bhagwat A, Jijina P. A Psychosocial Lens on an Indigenous Initiative to Address Menstrual Health and Hygiene in Indian Villages. Soc Work Public Health. 2020;35(3):73–89.

62. Freeman S, Eddy SL, McDonough M, Smith MK, Okoroafor N, Jordt H, et al. Active learning increases student performance in science, engineering, and mathematics. Proc Natl Acad Sci U S A. 2014;111(23):8410–5.

63. Dewey J. Experience and Education [1938]. 1938;

64. Vygotsky L. Interaction between learning and development. Readings Dev Child. 1978;23(3):34–41.

65. Achkovska Leshkovska E, Miovska Spaseva S. John Dewey'S educational theory and educational implications of Howard Gardner's multiple intelligences theory. Int J Cogn Res Sci Eng Educ. 2016;4(2):57–66.

66. DfE. Relationships Education, Relationships and Sex Education (RSE) and Health Education. 2019;(November):1–6.

67. Lock A, Strong T, editors. Introduction. In: Social Constructionism: Sources and Stirrings in Theory and Practice. Cambridge: Cambridge University Press; 2010. p. 1–11.

1. Chandra-Mouli V, Patel S V. Mapping the knowledge and understanding of menarche, menstrual hygiene and menstrual health among adolescent girls in low- and middle-income countries. Reprod Health. 2017;14.

2. Marván ML, Alcalá-Herrera V. Age at Menarche, Reactions to Menarche and Attitudes towards Menstruation among Mexican Adolescent Girls. J Pediatr Adolesc Gynecol.

[Type here]

For peer review only

2014;27(2):61–6.

3. Mason L, Nyothach E, Alexander K, Odhiambo FO, Eleveld A, Vulule J, et al. “We keep it secret so no one should know” - A qualitative study to explore young schoolgirls attitudes and experiences with menstruation in rural Western Kenya. PLoS One. 2013;8(11).

4. Setyowati S, Rizkia M, Ungsianik T. Improving Female Adolescents’ Knowledge, Emotional Response, and Attitude toward Menarche following Implementation of Menarcheal Preparation Reproductive Health Education. Asian/Pacific Isl Nurs J. 2019;4(2):84–91.

5. Shah V, Nabwera HM, Sosseh F, Jallow Y, Comma E, Keita O. A rite of passage : a mixed methodology study about knowledge , perceptions and practices of menstrual hygiene management in rural Gambia. BMC Public Health. 2019;19(277):1–15.

6. Sharma R, Sandhya N, Deepika K, Varsha S, Vardha. Menstrual hygiene among adolescent girls. Indian J Community Heal. 2015;27(3):376–80.

7. Marván ML, Alcalá-Herrera V. Age at menarche, reactions to menarche and attitudes towards menstruation among Mexican adolescent girls. J Pediatr Adolesc Gynecol. 2014;27(2):61–6.

8. Coast E, Lattof SR, Strong J. Puberty and menstruation knowledge among young adolescents in low- and middle-income countries: a scoping review. Vol. 64, International Journal of Public Health. Springer International Publishing; 2019. p. 293–304.

9. Ramaiya A, Malhotra A, Cronin C, Stevens S, Kostizak K, Sharma A, et al. How does a Social and Behavioral Change Communication Intervention Predict Menstrual Health and Hygiene Management: A Cross-Sectional Study. BMC Public Health. 2019;19(1):1–12.

10. Nalugya R, Tanton C, Hytti L, Kansiime C, Nakuya K, Namirembe P, et al. Assessing the effectiveness of a comprehensive menstrual health intervention program in Ugandan schools (MENISCUS): process evaluation of a pilot intervention study. Pilot Feasibility Stud. 2020;6(1):1–15.

11. House S, Mahon T, Cavill S. Menstrual Hygiene Matters: a resource for improving menstrual hygiene around the world. Reprod Health Matters. 2013 Jan;21(41):257–9.

12. Hennegan JM. Menstrual Hygiene Management and Human Rights: The Case for an Evidence-Based Approach. Women’s Reprod Heal. 2017;

13. Ellis A, Haver J, Villasenor J, Parawan A, Venkatesh M, Freeman MC, et al. WASH challenges to girls’ menstrual hygiene management in Metro Manila, Masbate, and South Central Mindanao, Philippines. Waterlines. 2016;

14. Rani A, Sharma MK, Singh A. Practices and perceptions of adolescent girls regarding the impact of dysmenorrhea on their routine life: A comparative study in the urban, rural, and slum areas of Chandigarh. Int J Adolesc Med Health. 2016;28(1):3–9.

15. Sumpter C, Torondel B. A Systematic Review of the Health and Social Effects of Menstrual Hygiene Management. PLoS One. 2013;8(4).

16. Tiwari A, Ekka IJ, Thakur R. Assessment of knowledge and practices regarding menstrual hygiene among adolescent girls of Government higher secondary school, station Murhipar, Rajnandgaon (C.G.). Int J Community Med Public Heal. 2018;5(4):1335–8.

17. Sommer M, Kjellén M, Pensulo C. Girls’ and women’s unmet needs for menstrual hygiene management (MHM): The interactions between MHM and sanitation systems in low-income countries. Vol. 3, Journal of Water Sanitation and Hygiene for Development. 2013. p. 283–97.

For peer review only - http://bmjopen.bmj.com/site/about/guidelines.xhtml

[Type here]

18. Hennegan J. Inserting informed choice into global menstrual product use and provision. Lancet Public Heal. 2019;4(8):e361–2.

19. Hennegan J, Dolan C, Steinfield L, Montgomery P. A qualitative understanding of the effects of reusable sanitary pads and puberty education: implications for future research and practice. Reprod Health. 2017;14.

20. Kansiime C, Hytti L, Nalugya R, Nakuya K, Namirembe P, Nakalema S, et al. Menstrual health intervention and school attendance in Uganda (MENISCUS-2): A pilot intervention study. BMJ Open. 2020;10(2):1–11.

21. Sebert Kuhlmann A, Peters Bergquist E, Danjoint D, Wall LL. Unmet Menstrual Hygiene Needs Among Low-Income Women. Obstet Gynecol. 2019;133(2):238–44.

22. PHS. Lifting the lid on period poverty. 2019.

23. Whitehead S, Biddle S. Adolescent girls' perceptions of physical activity: A focus group study. Eur Phys Educ Rev. 2008;14(2):243–62.

24. Biddle SJH, Whitehead SH, O'Donovan TM, Nevill ME. Correlates of participation in physical activity for adolescent girls: A systematic review of recent literature. J Phys Act Heal. 2005;2(4):423–34.

25. Gultie T, Hailu D, Workineh Y. Age of Menarche and Knowledge about Menstrual Hygiene Management among Adolescent School Girls in Amhara Province, Ethiopia: Implication to Health Care Workers & School Teachers. PLoS One. 2014;9(9).

26. Al Omari O, Razeq NMA, Fooladi MM. Experience of Menarche Among Jordanian Adolescent Girls: An Interpretive Phenomenological Analysis. J Pediatr Adolesc Gynecol. 2016;29(3):246–51.

27. Raskova M, Stolinska DP. COMMUNICATION ABOUT PUBERTY AMONG MIDDLE-SCHOOL-AGED CHILDREN. In: Bekirogullari Z, Minas MY, Thambusamy RX, editors. International Conference on Education and Educational Psychology. 2017. p. 194–202.

28. Bennett C, Harden J. An exploration of mothers' attitudes towards their daughters' menarche. Sex Educ Soc Learn. 2014;14(4):457–70.

29. Afsari A. Predictors of knowledge of puberty health among girl students. Biosci Biotechnol Res Commun. 2017;10(2):264–9.

30. Sooki Z, Shariati M, Chaman R, Khosravi A, Effatpanah M, Keramat A. The Role of Mother in Informing Girls About Puberty: A Meta-Analysis Study. Nurs Midwifery Stud. 2016;Inpress(Inpress):1–10.

31. Valizadeh S, Assdollahi M, Mirghafourvand M, Afsari A. Educating Mothers and Girls about Knowledge and Practices toward Puberty Hygiene in Tabriz, Iran: A Randomized Controlled Clinical Trial. Iran Red Crescent Med J. 2017;19(2).

32. Tingle, C & Vora S. Break the Barriers : Girls ' Experiences of. Plan Int UK. 2018;1–68.

33. Collier-Harris CA, Goldman JDG. Is puberty education evident in Australia's first national curriculum? Sex Educ Soc Learn. 2017;17(1):57–72.

34. Sumpter C, Torondel B. A Systematic Review of the Health and Social Effects of Menstrual Hygiene Management. PLoS One. 2013;8(4).

35. Hennegan J, Montgomery P. Do menstrual hygiene management interventions improve

[Type here]

For peer review only - http://bmjopen.bmj.com/site/about/guidelines.xhtml

education and psychosocial outcomes for women and girls in low and middle income countries? A systematic review. PLoS One. 2016;11(2):1–21.

36. Gignac GE, Szodorai ET. Effect size guidelines for individual differences researchers. Pers Individ Dif. 2016;102(December):74–8.

37. Hemphill JF. Interpreting the Magnitudes of Correlation Coefficients. Am Psychol. 2003;58(1):78–9.

38. Thomas J, Harden A. Methods for the thematic synthesis of qualitative research in systematic reviews. BMC Med Res Methodol. 2008;8(45):1–10.

39. Harris K, Kneale D, Tj L, Vm M, Grigg J, Thomas J, et al. School-based self-management interventions for asthma in children and adolescents : a mixed methods systematic review (Review). 2019;

40. Montgomery P, Hennegan J, Dolan C, Wu M, Steinfield L, Scott L. Menstruation and the Cycle of Poverty: A Cluster Quasi-Randomised Control Trial of Sanitary Pad and Puberty Education Provision in Uganda. PLoS One. 2016;11(12).

41. Phillips-Howard PA, Nyothach E, Ter Kuile FO, Omoto J, Wang D, Zeh C, et al. Menstrual cups and sanitary pads to reduce school attrition, and sexually transmitted and reproductive tract infections: A cluster randomised controlled feasibility study in rural Western Kenya. BMJ Open. 2016;

42. Van Eijk AM, Sivakami M, Thakkar MB, Bauman A, Laserson KF, Coates S, et al. Menstrual hygiene management among adolescent girls in India: A Systematic review and meta-analysis. BMJ Open. 2016;6(3).

43. Afsari A, Mirghafourvand M, Valizadeh S, Abbasnezhadeh M, Galshi M, Fatahi S. The effects of educating mothers and girls on the girls' attitudes toward puberty health: A randomized controlled trial. Int J Adolesc Med Health. 2015;2015.

44. Hurwitz LB, Lovato SB, Lauricella AR, Woodruff TK, Patrick E, Wartella E. "A New You, That's Who": an evaluation of short videos on puberty and human reproduction. Palgrave Commun. 2018;4(1).

45. Altundağ S, Çalbayram NÇ. Teaching menstrual care skills to intellectually disabled female students. Mol Ecol. 2016;25(13–14):1962–8.

46. Ariyanti TD, Royanto LRM. The Effectiveness of Social Stories and Video Modeling in Improving Self-Care Skills in Female Adolescents with Mild Intellectual Disabilities during Menstrual Periods. 2018;135(Iciap 2017):189–99.

47. Rani Chadalawada U, Devi S A, Rani M S. Effect of Health Education on Adolescent Girls Regarding Knowledge About Menstruation. J Evol Med Dent Sci. 2017;6(13):1040–3.

48. Jarrahi R, Golmakani N, Mazlom SR. Effect of menstrual hygiene education based on peer and small group teaching methods on hygiene behaviors in female adolescents: A comparative study. Evid Based Care J. 2020;10(1):70–4.

49. Van Eijk AM, Laserson KF, Nyothach E, Oruko K, Omoto J, Mason L, et al. Use of menstrual cups among school girls: Longitudinal observations nested in a randomised controlled feasibility study in rural western Kenya. Reprod Health. 2018;15(1):1–11.

50. Mokari H, Khaleghparast S, Samani LN. Impact of Puberty Health Education on Anxiety of Adolescents. Int J Med Res Heal Sci. 2016;5(5, S, SI):284–91.

For peer review only - http://bmjopen.bmj.com/site/about/guidelines.xhtml

[Type here]

51. Su JJ, Lindell D. Promoting the menstrual health of adolescent girls in China. Nurs Heal Sci. 2016;18(4):481–7.

52. Blake S, Boone M, Yenew Kassa A, Sommer M. Teaching Girls About Puberty and Menstrual Hygiene Management in Rural Ethiopia: Findings From a Pilot Evaluation. J Adolesc Res. 2018;33(5):623–46.

53. Haque SE, Rahman M, Itsuko K, Mutahara M, Sakisaka K. The effect of a school-based educational intervention on menstrual health: An intervention study among adolescent girls in Bangladesh. BMJ Open. 2014;4(7):1–9.

54. Morrison J, Basnet M, Bhatt A, Khimbanjar S, Chaulagain S, Sah N, et al. Girls' menstrual management in five districts of Nepal: Implications for policy and practice. Stud Soc Justice. 2018;12(2):251–72.

55. Sivakami M, van Eijk AM, Thakur H, Kakade N, Patil C, Shinde S, et al. Effect of menstruation on girls and their schooling, and facilitators of menstrual hygiene management in schools: Surveys in government schools in three states in India, 2015. J Glob Health. 2019;9(1).

56. Kheirollahi F, Rahimi Z, Arsang-Jang S, Sharifirad G, Sarraf P, Gharlipour Z. Puberty health status among adolescent girls: A model- based educational program. Int J Pediatr. 2017;5(7):5369–78.

57. Phillips-Howard PA, Nyothach E, Ter Kuile FO, Omoto J, Wang D, Zeh C, et al. Menstrual cups and sanitary pads to reduce school attrition, and sexually transmitted and reproductive tract infections: A cluster randomised controlled feasibility study in rural Western Kenya. BMJ Open. 2016;6(11):1–11.

58. Arasteh FE, Shobeiri F, Parsa P, Mohamadi Y. THE EFFECT OF COUNSELING ON KNOWLEDGE, ATTITUDE AND PRACTICE OF PUBERTY HEALTH AMONG ADOLESCENT GIRLS LIVING IN WELFARE BOARDING CENTERS. Indo Am J Pharm Sci. 2018;5(1):11–6.

59. Haque SE, Rahman M, Itsuko K, Mutahara M, Sakisaka K. The effect of a school-based educational intervention on menstrual health: An intervention study among adolescent girls in Bangladesh. BMJ Open. 2014;4(7):1–9.

60. United Nations Children's Fund (UNICEF) and World Health Organization 2018. Drinking Water, Sanitation and Hygiene in Schools : Global baseline report 2018. Unicef. 2018. 82 p.

61. Bhagwat A, Jijina P. A Psychosocial Lens on an Indigenous Initiative to Address Menstrual Health and Hygiene in Indian Villages. Soc Work Public Health. 2020;35(3):73–89.

62. Freeman S, Eddy SL, McDonough M, Smith MK, Okoroafor N, Jordt H, et al. Active learning increases student performance in science, engineering, and mathematics. Proc Natl Acad Sci U S A. 2014;111(23):8410–5.

63. Dewey J. Experience and Education [1938]. 1938;

64. Vygotsky L. Interaction between learning and development. Readings Dev Child. 1978;23(3):34–41.

65. Achkovska Leshkovska E, Miovska Spaseva S. John Dewey'S educational theory and educational implications of Howard Gardner's multiple intelligences theory. Int J Cogn Res Sci Eng Educ. 2016;4(2):57–66.

66. DfE. Relationships Education, Relationships and Sex Education (RSE) and Health Education. 2019;(November):1–6.

[Type here]

For peer review only - http://bmjopen.bmj.com/site/about/guidelines.xhtml

[Type here]

67. Lock A, Strong T, editors. Introduction. In: Social Constructionism: Sources and Stirrings in Theory and Practice. Cambridge: Cambridge University Press; 2010. p. 1–11.

Tables and Figures

Table 1 Search Terms Used

Table 2 Characteristics of Interventions

Table 3 Effect Size of Interventions

Figure 2 The PRISMA Flow-chart

Supplementary File Characteristics and Quality of Included Studies

ADDITIONAL INFORMATION

Contributors

RE, FG and BH designed the review. RE and OC reviewed titles, abstracts and full-texts for eligibility. Disagreement was resolved by discussion and where necessary FG and BH offered their view. RE, FG and BH agreed on a data extraction framework, which was then carried out by RE. The quality assessment tool was agreed upon by RE, FG and BH. RE used the MMAT to assess the quality and this was verified by FG and BH. RE prepared the manuscript and it was reviewed and edited by FG and BH.

Corresponding author

Rebecca.R.Evans@warwick.ac.uk

Funding Statement

This work was supported by the National Institute for Health Research Unit on Improving Health in Slums. Funder reference: 16/136/87.

Ethical Approval

BSREC 43/19-20

Competing Interests

None declared

Patient Consent

None required

Data Sharing Statement

No original data were generated in this study

Word Count 3955

[Type here]

For peer review only

[Type here]

[Type here]

For peer review only

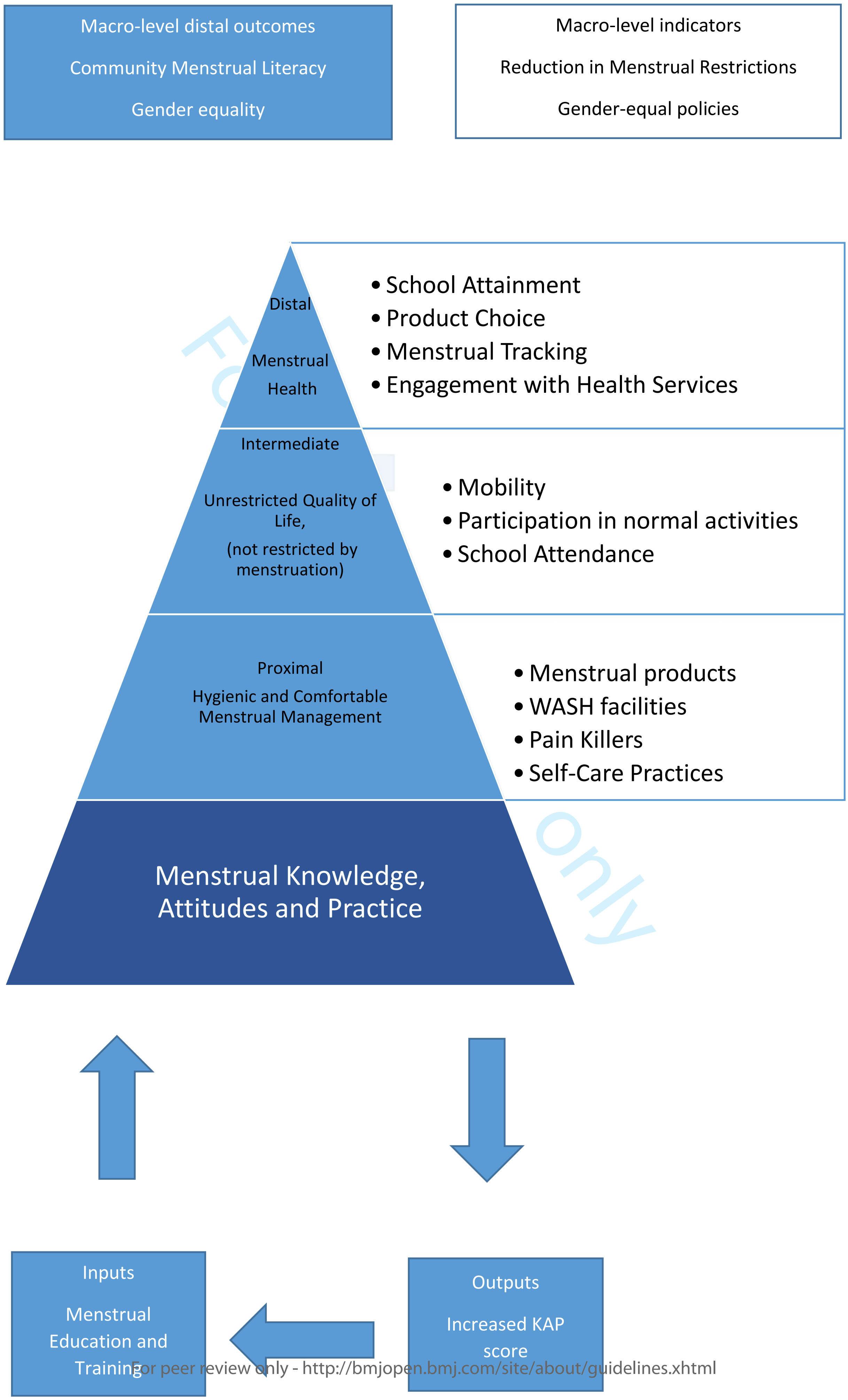
Macro-level distal outcomes
Community Menstrual Literacy
Gender equality
Macro-level indicators
Reduction in Menstrual Restrictions
Gender-equal policies
Distal
Menstrual Health
• School Attainment
• Product Choice
• Menstrual Tracking
• Engagement with Health Services
Intermediate
Unrestricted Quality of Life,
(not restricted by menstruation)
• Mobility
• Participation in normal activities
• School Attendance
Proximal
Hygienic and Comfortable Menstrual Management
• Menstrual products
• WASH facilities
• Pain Killers
• Self-Care Practices
Menstrual Knowledge, Attitudes and Practice
Inputs
Menstrual Education and Training
Outputs
Increased KAP score

For peer review only

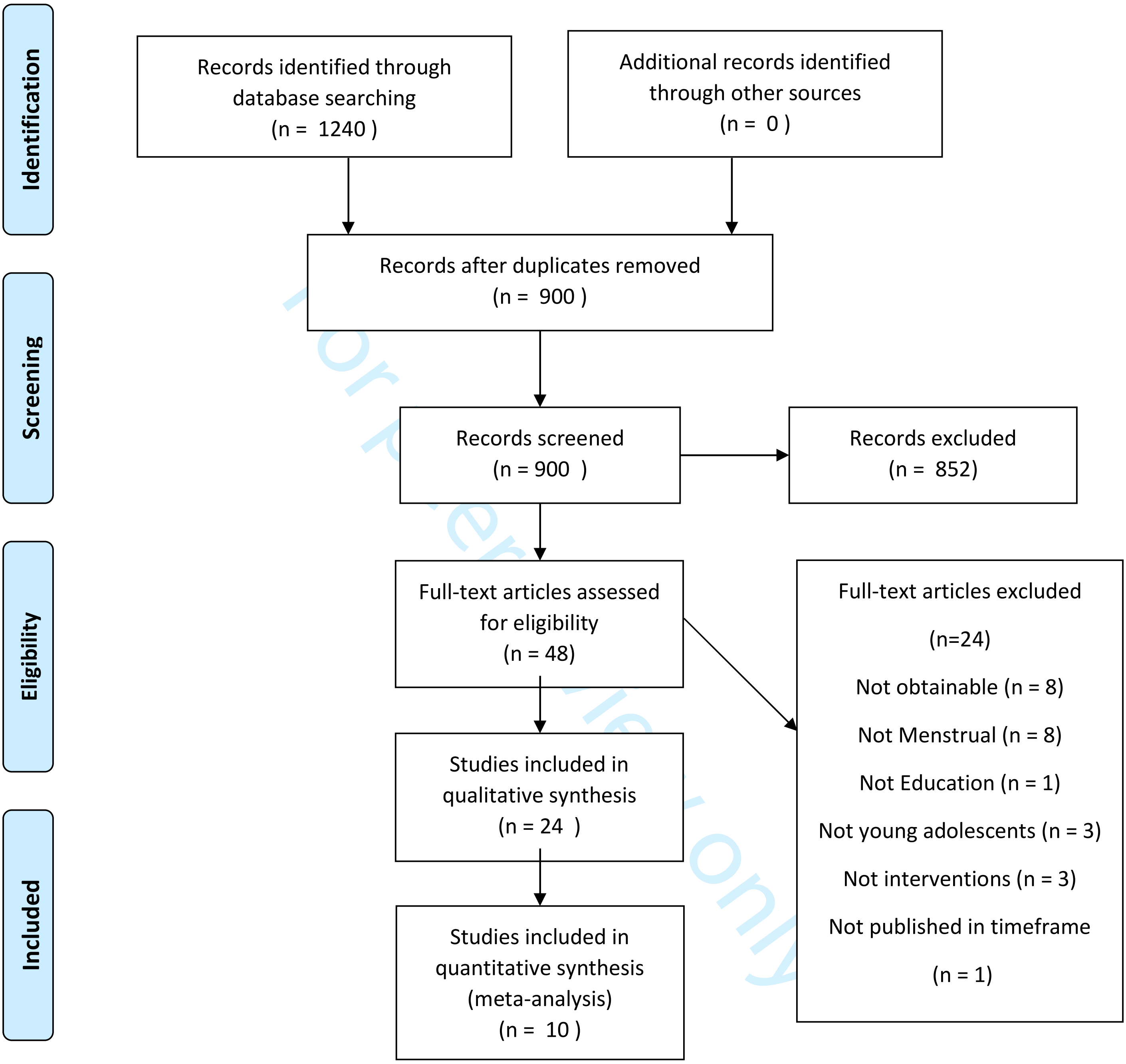

Figure 2. PRISMA flow chart

Supplementary File: Characteristics and quality of included studies

Citation	Design	Country	Study population	Sampling Method	No of participants	Type of intervention	MMAT Clear Research Questions /2	MMAT Appropriate Data Collection?/2	MMAT Supplementary questions relevant to methodology/5	MMAT Total/9	MMAT RAG rating
(Afsari *et al.*, 2015)	RCT quasi-experimental pre-test, post test	Iran	Mothers and adolescent school girls	Random Cluster-sampling	364	Half hour lecture and pamphlet distribution to improve attitude	2	1	5	8	Green
(Altundağ and Çalbayram, 2016)	Quantitative Descriptive Case Series Quasi-experimental pre-test, post-test	Turkey	Intellectually-disabled girls in special education training centre	convenience	77	Demonstration on doll to improve Pad-replacement skills	2	2	4	8	Green
(Arasteh *et al.*, 2019)	Quantitative descriptive one group pre-test, post-test	Iran	All adolescent girls in two welfare	Purposive sampling	30	Group counselling in boarding centres	2	2	3	7	Amber

For peer review only

			boarding centres								
(Ariyanti and Royanto, 2018)	Quantitative descriptive case report; pre-test, post-test	Indonesia	Mild-intellectually disabled girl	Purposive sampling	1	Stories and video-modelling	2	2	3	7	Amber
(Blake *et al.*, 2018)	Mixed methods, quantitative survey pre-test, post-test	Ethiopia	Girls enrolled in school in grades 6 and 7	Cluster randomised approach	636	Puberty Book 'Growth and Changes'	2	2	5	9	Green
(Rani Chadalawada, Devi S and Rani M, 2017)	RCT, pre-test, post-test	India	Adolescent high school girls aged 11 – 19 years of Zilla Parishad High School	Systematic random technique (every other girl on register)	250	Chalk and talk, posters, flipcharts,	1	2	2	5	Red
(Haque *et al.*, 2014a)	Quantitative descriptive one group, Pre-test, post-test	Bangladesh	adolescent female students aged 11–16 years, in grade 6–8, and living with their parents.	Random cluster sampling	416	Field manual training	2	2	3	7	Amber
(Hennegan and Montgomery, 2016b)	Qualitative understanding of quasi-RCT	Uganda	school girls in eight study schools in primary classses grades 4 - 7.	Quasi-randomised cluster sampling	27: education (n=8) pads alone (n=8) education and pads (n=6) and	75 minute session of Straight Talk Education programme	2	2	5	9	Green

For peer review only

					control (n=5)						
(Hurwitz *et al.*, 2018)	RCT pre-test post-test	USA	Children of both sexes whose parents had registered an interest	Random sampling	80 (43 girls)	'A New You, that's Who' video series	2	2	3	7	Amber
(Jarrahi, Golmakani and Mazlom, 2020)	3 arm RCT Control and 2 intervention: peer and small group teaching pre-test/post-test	Iran	Adolescent school girls	Random sampling of three high schools	90	Small group and peer-teaching	2	2	3	7	Amber
(Kansiime *et al.*, 2020)	Mixed methods Longitudinal study	Uganda	Girls and boys in secondary school	Purposive selection of schools	369	Multi-component approach to optimising government guidelines for puberty education	2	2	5	9	Green
(Kheirollahi *et al.*, 2017)	Quasi-experimental RCT, and pre-test, post- test	Iran	School girls	Random cluster sampling	152	A model-based educational program	1	2	2	5	Red
(Mokari, Khaleghparast and Samani, 2016)	Quasi-experimental RCT	Iran	School girls aged 14 – 16 years and their parents	Random cluster sampling	159	Teaching programme with discussion	2	2	3	7	Amber
(Montgomery *et al.*, 2016)	four-armed quasi-experimental RCT Pre and post test	Uganda	Girls in grades 3 - 5	Randomised	1124	Educational arm used Straight Talk	1	2	0	3	Amber

(Morrison *et al.*, 2018)	Mixed Methods Quantitative cross-sectional surveys and qualitative focus group	Nepal	Girls in grades seven to ten in 28 large schools.	Random cluster sampling	860	WASH in Schools (WinS) programme	1	2	4	7	Amber
(Nalugya *et al.*, 2020)	Mixed methods process evaluation Longitudinal study	Uganda	Girls and boys in secondary school	Purposive selection of schools	369	Multi-component approach to optimising government guidelines for puberty education	2	2	5	9	Green
(Penelope A. Phillips-Howard *et al.*, 2016)	3-arm single-site open cluster randomised controlled pilot study.	Kenya	Primary girls aged 14 -16	Random cluster sampling	644	Puberty education and menstrual cup instruction from nurse	1	2	5	8	Green
(Ramaiya *et al.*, 2019)	Quantitative non-random post-test only	India	Adolescent girls	Purposive (schools) and random (girls)	2206	Monthly discussion group	2	2	5	9	Green
(Setyowati, Rizkia and Ungsianik, 2019)	Quasi-experimental Control/intervention pre-test post-test	Indonesia	Girls aged 9-12 pre-menarch	Stratified random sampling technique	174	Booklet	2	2	2	6	Amber
(Sharma *et al.*, 2015)	Experimental; Intervention and control; pre-test, post-test	India	Adolescent girls at a government high school	Randomly sampled	50	One training session on menstrual hygiene	1	0	1	2	Red
(Sivakami *et al.*, 2019)	Quantitative descriptive Pre-test, post-test	India	Adolescent girls	Multi-level stratified	2564	'model' schools received	1	2	5	8	Green

				sampling of schools		additional WASH support and education embedded into curriculum					
(Su and Lindell, 2016)	A quasi-experimental RCT two group pretest-posttest design	China	Adolescent girls	Purposive sampling of schools	116	Researcher – led Menstrual Hygiene Class	2	2	4	8	Green
(Van Eijk *et al.*, 2016)	Mixed methods. Qualitative review and semi-quantitative measure of cup use nested in larger RCT	Kenya	Post-menarche adolescent girls	Random allocation of 10 schools to menstrual cup arm	192	Puberty education and menstrual cup instruction from nurse	1	2	5	8	Green
(Valizadeh *et al.*, 2017)	RCT three arm – educating girls, educating mothers or control. pre and post test	Iran	Post-menarche adolescent school girls	12 purposively sampled schools	327	30 minute lecture on puberty hygiene plus a booklet	0	1	5	6	Amber

Summary of characteristics of included studies

All studies were interrogated with two questions 'Are there clear research questions?' and 'Do the collected data allow the research questions to be addressed?' which were considered fundamental to the quality and were scored on a scale of 'Yes' = 2, 'not clear' = 1 and 'No' = 0. Five further supplementary questions were considered that addressed quality issues such as sample size. The sets of questions were different depending upon the study design, and are not directly comparable, so less weight was given to these; Yes = 1 and No = 0. The Best Fit principle was used. The maximum score when added together was 2 + 2 + 5 = 9. Studies scored 0-5 were categorised as low quality (as it was possible to get these scores without clear research questions or valid methods); those that scored 6 or 7 were scored as moderate quality and those that scored 8 or 9 were scored as high quality.

For peer review only

For peer review only

PRISMA 2020 Checklist

Section and Topic	Item #	Checklist item	Location where item is reported
**TITLE**			
Title	1	Identify the report as a systematic review.	1
**ABSTRACT**			
Abstract	2	See the PRISMA 2020 for Abstracts checklist.	
**INTRODUCTION**			
Rationale	3	Describe the rationale for the review in the context of existing knowledge.	3-4
Objectives	4	Provide an explicit statement of the objective(s) or question(s) the review addresses.	4
**METHODS**			
Eligibility criteria	5	Specify the inclusion and exclusion criteria for the review and how studies were grouped for the syntheses.	4
Information sources	6	Specify all databases, registers, websites, organisations, reference lists and other sources searched or consulted to identify studies. Specify the date when each source was last searched or consulted.	5
Search strategy	7	Present the full search strategies for all databases, registers and websites, including any filters and limits used.	5
Selection process	8	Specify the methods used to decide whether a study met the inclusion criteria of the review, including how many reviewers screened each record and each report retrieved, whether they worked independently, and if applicable, details of automation tools used in the process.	5
Data collection process	9	Specify the methods used to collect data from reports, including how many reviewers collected data from each report, whether they worked independently, any processes for obtaining or confirming data from study investigators, and if applicable, details of automation tools used in the process.	6
Data items	10a	List and define all outcomes for which data were sought. Specify whether all results that were compatible with each outcome domain in each study were sought (e.g. for all measures, time points, analyses), and if not, the methods used to decide which results to collect.	6
	10b	List and define all other variables for which data were sought (e.g. participant and intervention characteristics, funding sources). Describe any assumptions made about any missing or unclear information.	6
Study risk of bias assessment	11	Specify the methods used to assess risk of bias in the included studies, including details of the tool(s) used, how many reviewers assessed each study and whether they worked independently, and if applicable, details of automation tools used in the process.	6
Effect measures	12	Specify for each outcome the effect measure(s) (e.g. risk ratio, mean difference) used in the synthesis or presentation of results.	6-7
Synthesis methods	13a	Describe the processes used to decide which studies were eligible for each synthesis (e.g. tabulating the study intervention characteristics and comparing against the planned groups for each synthesis (item #5)).	7
	13b	Describe any methods required to prepare the data for presentation or synthesis, such as handling of missing summary statistics, or data conversions.	7
	13c	Describe any methods used to tabulate or visually display results of individual studies and syntheses.	7
	13d	Describe any methods used to synthesize results and provide a rationale for the choice(s). If meta-analysis was performed, describe the model(s), method(s) to identify the presence and extent of statistical heterogeneity, and software package(s) used.	7
	13e	Describe any methods used to explore possible causes of heterogeneity among study results (e.g. subgroup analysis, meta-regression).	
	13f	Describe any sensitivity analyses conducted to assess robustness of the synthesized results.	
Reporting bias assessment	14	Describe any methods used to assess risk of bias due to missing results in a synthesis (arising from reporting biases).	7
Certainty	15	Describe any methods used to assess certainty (or confidence) in the body of evidence for an outcome.	7

For peer review only - http://bmjopen.bmj.com/site/about/guidelines.xhtml

PRISMA 2020 Checklist

Section and Topic	Item #	Checklist item	Location where item is reported
assessment			
**RESULTS**			
Study selection	16a	Describe the results of the search and selection process, from the number of records identified in the search to the number of studies included in the review, ideally using a flow diagram.	8
	16b	Cite studies that might appear to meet the inclusion criteria, but which were excluded, and explain why they were excluded.	
Study characteristics	17	Cite each included study and present its characteristics.	9
Risk of bias in studies	18	Present assessments of risk of bias for each included study.	SF
Results of individual studies	19	For all outcomes, present, for each study: (a) summary statistics for each group (where appropriate) and (b) an effect estimate and its precision (e.g. confidence/credible interval), ideally using structured tables or plots.	12
Results of syntheses	20a	For each synthesis, briefly summarise the characteristics and risk of bias among contributing studies.	12
	20b	Present results of all statistical syntheses conducted. If meta-analysis was done, present for each the summary estimate and its precision (e.g. confidence/credible interval) and measures of statistical heterogeneity. If comparing groups, describe the direction of the effect.	12
	20c	Present results of all investigations of possible causes of heterogeneity among study results.	SF
	20d	Present results of all sensitivity analyses conducted to assess the robustness of the synthesized results.	SF
Reporting biases	21	Present assessments of risk of bias due to missing results (arising from reporting biases) for each synthesis assessed.	SF
Certainty of evidence	22	Present assessments of certainty (or confidence) in the body of evidence for each outcome assessed.	SF
**DISCUSSION**			
Discussion	23a	Provide a general interpretation of the results in the context of other evidence.	16
	23b	Discuss any limitations of the evidence included in the review.	17
	23c	Discuss any limitations of the review processes used.	17
	23d	Discuss implications of the results for practice, policy, and future research.	17
**OTHER INFORMATION**			
Registration and protocol	24a	Provide registration information for the review, including register name and registration number, or state that the review was not registered.	17
	24b	Indicate where the review protocol can be accessed, or state that a protocol was not prepared.	
	24c	Describe and explain any amendments to information provided at registration or in the protocol.	
Support	25	Describe sources of financial or non-financial support for the review, and the role of the funders or sponsors in the review.	26
Competing interests	26	Declare any competing interests of review authors.	26
Availability of data, code and other materials	27	Report which of the following are publicly available and where they can be found: template data collection forms; data extracted from included studies; data used for all analyses; analytic code; any other materials used in the review.	

For peer review only

PRISMA 2020 Checklist

From: Page MJ, McKenzie JE, Bossuyt PM, Boutron I, Hoffmann TC, Mulrow CD, et al. The PRISMA 2020 statement: an updated guideline for reporting systematic reviews. BMJ 2021;372:n71. doi: 10.1136/bmj.n71

For more information, visit: http://www.prisma-statement.org/

For peer review only

BMJ Open

A Systematic Review of Educational Interventions to improve the Menstrual Health of young adolescent girls.

Journal:	*BMJ Open*
Manuscript ID	bmjopen-2021-057204.R1
Article Type:	Original research
Date Submitted by the Author:	23-Feb-2022

Complete List of Authors:	Evans, Rebecca; University of Warwick<br>Harris, Bronwyn; Warwick Medical School, University of Warwick, Coventry, Division of Health Sciences, ; University of the Witwatersrand, Centre for Health Policy, School of Public Health<br>Onuegbu, Chinwe ; University of Warwick, Health Sciences<br>Griffiths, Frances; University of Warwick, Warwick Medical School

**Primary Subject Heading**:	Public health
Secondary Subject Heading:	Sexual health
Keywords:	PUBLIC HEALTH, Health policy < HEALTH SERVICES ADMINISTRATION & MANAGEMENT, REPRODUCTIVE MEDICINE, PREVENTIVE MEDICINE

SCHOLARONE™
Manuscripts

I, the Submitting Author has the right to grant and does grant on behalf of all authors of the Work (as defined in the below author licence), an exclusive licence and/or a non-exclusive licence for contributions from authors who are: i) UK Crown employees; ii) where BMJ has agreed a CC-BY licence shall apply, and/or iii) in accordance with the terms applicable for US Federal Government officers or employees acting as part of their official duties; on a worldwide, perpetual, irrevocable, royalty-free basis to BMJ Publishing Group Ltd ("BMJ") its licensees and where the relevant Journal is co-owned by BMJ to the co-owners of the Journal, to publish the Work in this journal and any other BMJ products and to exploit all rights, as set out in our licence.

The Submitting Author accepts and understands that any supply made under these terms is made by BMJ to the Submitting Author unless you are acting as an employee on behalf of your employer or a postgraduate student of an affiliated institution which is paying any applicable article publishing charge ("APC") for Open Access articles. Where the Submitting Author wishes to make the Work available on an Open Access basis (and intends to pay the relevant APC), the terms of reuse of such Open Access shall be governed by a Creative Commons licence – details of these licences and which Creative Commons licence will apply to this Work are set out in our licence referred to above.

Other than as permitted in any relevant BMJ Author's Self Archiving Policies, I confirm this Work has not been accepted for publication elsewhere, is not being considered for publication elsewhere and does not duplicate material already published. I confirm all authors consent to publication of this Work and authorise the granting of this licence.

For peer review only

[Type here]

TITLE PAGE

Title:

A systematic Review of Educational Interventions to improve the Menstrual Health of young adolescent girls.

Authors:
Ms Rebecca Lane-Evans, Warwick Medical School, Warwick Medical School, University of Warwick, Coventry, CV4 7AL United Kingdom. Rebecca.R.Evans@warwick.ac.uk

Ms Chinwe Onuegbu, Warwick Medical School, Warwick Medical School, University of Warwick, Coventry, CV4 7AL United Kingdom. Chinwe.Onuegbu@warwick.ac.uk

Dr Bronwyn Harris, Warwick Medical School, Warwick Medical School, University of Warwick, Coventry, CV4 7AL United Kingdom. B.Harris.2@warwick.ac.uk

Professor Frances Griffiths, Warwick Medical School, Warwick Medical School, University of Warwick, Coventry, CV4 7AL United Kingdom. F.E.Griffiths@warwick.ac.uk

Word Count: 4186

For peer review only

[Type here]

[Type here]

ABSTRACT

Objectives

To systematically review interventions that include an element of menstrual education delivered to young adolescent girls.

Design

This was a systematic review and meta-analysis. Selected articles were quality assessed using the Mixed Methods Assessment Tool (MMAT) quality appraisal checklist. A meta-analysis was conducted on a subset of articles that provided quantitative results, and the effect size of the intervention was calculated using Cohen's d. A logic model was constructed to frame the effect of Menstrual Education Interventions on Menstrual Health.

Setting

Papers reporting on interventions in High and Low-and Middle-Income countries were sought.

Information Sources

Seven electronic databases were searched for English-language entries that were published between January 2014 and May 2020.

Participants

The interventions were aimed at younger adolescent girls aged 10 -14 years old.

Interventions

The interventions were designed to improve the Menstrual Health of the recipients, by addressing one or more elements of Menstrual Knowledge, Attitude or Practices (KAP).

Eligibility criteria

Interventions in which there was a component of menstrual information transfer were included. Interventions which had not been evaluated were excluded.

Primary and Secondary Outcomes

The most common type of output was a difference in knowledge or skill score ascertained from a pre- and post-test. Some studies measured additional outcomes, such as attitude or confidence.

Results

Twenty-four eligible studies were identified. Nineteen addressed Menstrual Knowledge, and five addressed Menstrual Skills such as pad replacement or cup insertion. The number of participants varied from 1 to 2564. The total number of participants was 10362. All studies reported improvements in menstrual Knowledge, Attitudes and Practices. The meta-analysis indicates that larger effect sizes were attained by those that encouraged discussion than those that distributed pamphlets.

Conclusions

Education interventions are effective in increasing the menstrual knowledge of young adolescent girls and skills training improves competency to manage menstruation more hygienically and comfortably. Interactive interventions, with discussions, are more motivating than didactic or

[Type here]

For peer review only

written. Sharing concerns gives girls confidence and helps them to gain agency on the path to menstrual health.

STRENGTHS AND LIMITATIONS

- This review is of the international literature including high and low-and-middle-income countries
- It utilises mixed methods approaches and synthesises both quantitative and qualitative studies
- A logic model has been constructed to frame the effect of Menstrual Education Interventions on Menstrual Health
- There may be publication bias in the reporting of positive outcomes only
- A full meta-analysis was not possible due to heterogeneity in methods

INTRODUCTION

Globally, young adolescent girls are ill-prepared for menarche and menstruation (1–4). In many cultures, menstruation is a taboo subject (5) and many girls are ignorant of it until they start bleeding (6). Negative experiences of menarche and early menstruation can cause poor Menstrual Health (7,8).

'Menstrual Health' is an emerging area of Health Research. It is a broad term which encompasses the hygienic management of menstruation and the psychological components of well-being such as confidence, dignity and self-esteem (9,10). It is an expansion of the concept of Menstrual Hygiene Management (MHM), mostly used in Low- and Middle- Income Country (LMIC) contexts to describe the challenges of hygienically managing menstruation with a lack of resources, especially pads, water and soap (11–13). The use of dirty rags to absorb menstrual blood has been proposed as a cause of Reproductive Tract Infections (RTIs) and cervical cancer (14–16), as infectious agents may be introduced into the reproductive tract from such materials. Even clean materials that are not changed regularly may smell (3) and some materials can cause chafing and irritation. Without sufficient water to wash the blood from their genitals or their hands (17), girls can become uncomfortable and anxious (18). Several studies in LMIC have shown that menstruation is associated with a reduction in participation in activities and an increase in school absenteeism (15,19,20). In High Income Countries (HIC), 'Period Poverty' has only recently been recognised as an issue for certain groups, such as homeless women (21), but a whitepaper by PHS Group UK (a Hygiene Services provider in the UK) suggests period poverty is more wide-reaching, particularly amongst school girls, and is a factor contributing to menstrual anxiety and school absenteeism (22). Significantly less research has been conducted in HICs around menstruation and participation, although it is widely recognised that girls drop out of sports activities around the age of puberty (23,24).

For peer review only - http://bmjopen.bmj.com/site/about/guidelines.xhtml

[Type here]

Discussing menstruation is almost universally a taboo, making it difficult for girls to learn about it and know what is normal or when to seek help. A study by Gultie *et al* 2014 into Menstrual Knowledge of adolescents in Ethiopia found that 33% of participants never talked about it with anyone (25). In Jordan, a phenomenological analysis of experiences of menarche reported that girls believed talking about menstruation was 'rude' (26). A study into puberty communication in the Czech Republic and China found that both men and women were complicit in perpetuating menstrual stigma, with mothers instructing their daughters to keep their menstrual status 'secret' (27). In India, Rani *et al* 2016 found that although 61.3% of adolescent girls suffered from debilitating dysmenorrhea, they felt they were expected to 'tolerate' it as a natural process and only 1.6% had ever consulted a physician (14).

Menstrual knowledge usually comes from mothers (8) but a number of studies have shown that the knowledge of mothers themselves may be incomplete (28,29) and they may actually perpetuate cultural myths and misinformation (30,31). In HICs such as Australia and the UK, menstruation may be taught at school as part of sex education, if it is mentioned at all, but many girls do not get the practical information that they need (32,33). In both HIC and LMIC non-government agencies have stepped in to try to plug the gap by providing Menstrual Education Interventions.

In this review our objective was to describe and evaluate the impact of menstrual education interventions intended to equip young adolescent girls with the knowledge and skills to promote Menstrual Health.

Two previous reviews of papers published prior to January 2015 focussed on the more narrow 'Menstrual Hygiene Management' in LMIC (34,35). The term Menstrual Health is now preferred to hygiene, partly to avoid the suggestion that menstruation *per se* is unclean, but mostly to emphasize the holistic nature of the menstrual experience. This literature review only includes publications since 2014 in order to capture that change.

METHODS

This is a systematic review of published literature of interventions that include an element of menstrual education delivered to young adolescent girls. We report according to the Preferred Reporting Items for Systematic Reviews and Meta-Analyses (PRISMA) guidelines.

Patient and Public Involvement

No patient involvement.

Publication Date and Language

This review draws upon papers published from January 2014 until May 2020 to bring the field up to date. Only reviews published in the English language have been included.

Participants

For this review we were interested in interventions targeted at young adolescent girls aged 10 -14 years old.

[Type here]

For peer review only - http://bmjopen.bmj.com/site/about/guidelines.xhtml

[Type here]

Inclusion and Exclusion Criteria

Studies of interventions which had been evaluated were sought.

All interventions which had a component of menstrual information transfer were included if the intervention sought to:

- Increase knowledge of menstruation to reduce anxiety and shame, and normalise the experience
- Increase skills and competencies to manage menstruation comfortably and hygienically
- Increase awareness of strategies for self-care of menstrual symptoms

Interventions that were straightforward 'Menstrual Education' in which lessons about puberty, anatomy, and hygiene were delivered by teachers or other educators, to both boys and girls, were eligible. Interventions focussed on skills training, such as correct menstrual cup insertions, and delivered by nurses or other key workers were also eligible. Programmes that facilitated peer or self-guided learning through the provision of resources or spaces (both physical and remote) for learning to occur were also eligible for inclusion.

Studies were excluded if the improvements were in hardware such as toilets or pads without any accompanying education or training, or if they provided hygiene education without reference to menstruation specifically. Studies were excluded if they were about abnormal menstruation, menstrual problems as a co-morbidity, or if the research was investigating endocrinology or non-human models. Studies that described existing Knowledge, Attitudes and Practice without any intervention were also excluded.

Studies that included adolescents up to the age of 19 were not excluded if the aim were to instruct menstruators with limited experience of menstruation. Some studies included older girls because they were intellectually disabled and were part of the intervention based on intellectual age rather than chronological age. Some studies included older girls because they were members of classes assigned by grade rather than age. Studies about interventions aimed at adult women were excluded.

Study design and quality were not part of the criteria to capture as broad an interpretation of menstrual education as possible.

Protocol for Identification of Academic Literature

Screening

As the field of Menstrual Health Education is highly interdisciplinary, we searched key medical and social science databases: ASSIA Applied Social Science Index and Abstracts; CINAHL Cumulative Index to Nursing and Allied Health Literature; EMBASE Excerpta Medica database; MEDLINE Medical Literature Analysis and Retrieval System Online; Sociological Abstracts; Web of Science; IBBS International Bibliography of the Social Sciences; TRoPHI Trials Register of Promoting Health Interventions.

The search parameters combined the target population, menstruation, education and programme (table 1).

[Type here]

For peer review only - http://bmjopen.bmj.com/site/about/guidelines.xhtml

[Type here]

Table 1. Search Terms Used

**Search Terms Used**		
Search term 1<br>'adolescen* OR girl* OR teenage* OR youth* OR young OR pre-adolescen* OR school-girl OR Out-of-School-Youth OR OOSY OR female OR woman<br>AND Search term 2<br>Menstrua* OR menarche* OR mense* OR catamenia OR menarche* OR menstrual health OR menstrual hygiene OR menstrual management OR sanitation OR menstrual etiquette<br>AND Search term 3<br>know* OR understand*OR manage* OR learn* OR apprehen* OR comprehensi* OR educat* OR aware* OR familiar* OR proficien*<br>AND Search term 4<br>AND arrangement* OR evaluat* OR initiative* OR intervention*OR model* OR package* OR pilot* OR program* OR project* OR provision* OR regime* OR scheme* OR strateg* OR trial* OR approach* OR polic*		
**Database Returns**		
**Database**	**Search Terms and filters**	**Returns**
ASSIA (proquest)	1 (ab), 2 (ti), 3 (ab), 4 (ab)<br>01/01/14 to 01/09/19	15
CINAHL	1 (ab), 2 (ti), 3 (ab), 4 (ab)<br>01/01/14 to 01/09/19	126
EMBASE<br>Including MEDLINE	1 (ab), 2 (ti), 3 (ab), 4 (ab)<br>2014 -2019<br>English, Female	732
Sociological abstracts (proquest)	1 (ab), 2 (ti), 3 (ab), 4 (ab)<br>01/01/14 to 01/09/19	14
Web of Science	TS=(1), (3), (4) AND Ti=(2)<br>Last 5 years	323
IBBS (proquest)	1 (ab), 2 (ti), 3 (ab), 4 (ab)<br>01/01/14 to 01/09/19	26
TRoPHI (Eppi centre, google)	No wild cards<br>Title<br>Menstruation and Knowledge<br>2014-2019	4
**TOTAL SAVED to EXCEL**		**1240**

For peer review only

Two reviewers (RE and CO) screened abstracts and full texts of all citations obtained for eligibility independently. Data extraction of eligible material and the quality assessment was conducted by RE using a data extraction framework agreed upon by FG, BH and RE. The quality assessment tool was agreed upon by RE, FG and BH. RE used the MMAT to assess the quality and this was verified by FG and BH.

Outputs and Data Extraction

The outputs of Menstrual Education are changes to Menstrual Knowledge, Attitudes and Practices (KAP). In quantitative studies, Pre- and Post-Intervention KAP scores were collected, as well as data to support the validity of the scores, such as sample frame and number of participants. Other descriptive measures used in qualitative studies and contextual factors that might have a bearing on the outputs were also recorded where given.

[Type here]

[Type here]

Quality Assessment

We used the Mixed Methods Appraisal Tool (MMAT) for quality assessment because it is suitable for a variety of study designs. The MMAT components focus on the clarity of the research questions and the appropriateness of the data collection methods. Our intention was to consider the MMAT assessment in the interpretation of study findings.

Analysis and Synthesis

Quantitative analysis

The results of quantitative studies were tabulated for comparison. Not all data could be converted but for studies that reported quantitative results with a pre- and post- test score of Menstrual Knowledge, we found the effect size by calculating the Standard Mean Difference using Cohen's d. Effect sizes can lie between 0 and infinity. Cohen suggested that a small effect size is a value of 0.2, a medium one is 0.5 and a large one is 0.8. Some social science disciplines report much larger sizes and the statistical guidance was revised to suggest that a medium effect is the average of those in the relevant literature (36,37). However, Cohen's d has not previously been calculated for this discipline. We found the average of those in this review and ranked the results accordingly(38).

Qualitative analysis

A Qualitative Comparative Analysis (QCA) (39) was made of all studies by scrutinising the data extracted for common themes and reporting them in a narrative summary.

Synthesis: Building A Logic Model

Logic models enable the key findings of the analysis to be synthesized into a theory of change. We constructed a logic model to frame the effect of Menstrual Education Interventions on Menstrual Health.

RESULTS

A total of 1240 papers were recovered using the search terms. 900 remained after the removal of duplicates and 48 were saved to Excel for full text screening. 852 did not meet the inclusion criteria. This was largely because they were not interventions but studies of menstruation (Figure 1).

PRIFLO

Figure 1. PRISMA Flow-chart

Proximal, intermediate, distal and macro-distal outcomes and indicators of a menstrual health intervention. Menstrual Education and Training Inputs act on Girls' Menstrual Knowledge, Attitude and Practices, and Outputs are measured by increasing KAP score. Positive feedback operates as girls gain knowledge and skills they become more confident to seek further knowledge and skills.

Study Characteristics

Twenty-four papers met the inclusion criteria: They were grouped into the following categories of study design according to the MMAT Quality Appraisal tool: 12 Randomised Controlled Trials (RCTs); 5 quantitative descriptive studies; 1 quantitative non-random study; 5 mixed-methods studies and 1 qualitative study. *See Supplementary Files for further detail on study design.*

[Type here]

For peer review only

[Type here]

The study dates ranged from 2012 to 2017, with dates of publication ranging from 2014 to 2019. Studies were undertaken in Iran (six), Turkey, Indonesia (two), Ethiopia, India (four), Bangladesh, Uganda (four), USA, Nepal, Kenya (two) and China.

Of the studies, two included boys (10,20,40); one included mothers (29,31) and two focussed on intellectually-disabled adolescent girls (41,42). The number of study participants varied from 1 to 2564. The total number of participants in the 20 different intervention studies was 10362.

The amount of time after the completion of the intervention to the assessment varied from the same day to up to 5 years, with 13 of the studies in the range of 1 – 9 months, and the mode being 1 month. A common theme was allowing one menstrual cycle to pass before re-testing.

Delivery

Eight of the interventions used health professionals as instructors (6,9,10,31,41,43–46). Five were researcher-led (31,42,47–49); five were led by teachers (20,50–53); two used peer-educators (9,44) and the others did not make it clear. A wide range of intensity and duration models were used, ranging from three 5-minute videos in the space of one afternoon (40) to 1 hour per day for four months (43). Only two embedded menstrual education into the school curriculum (52,53).

Quality Assessment

The methodological quality of study designs was mixed: Eleven were rated as high quality and thirteen as moderate to low. Those considered to be of the highest quality were randomized controlled trials which included comparison groups. Some of the studies (nine) did this at the whole-school level which is recommended in educational interventions to prevent contamination of the intervention group with the control (54). The research questions were clear and the data collection methods appropriate. Of the other studies, several methodological limitations were noted; commonly, neither the delivery team nor the participants were blinded (nine); adequate randomization of the participants was lacking (ten) and /or relevant confounds were not identified or controlled (four). The quality of data analysis also varied considerably, with the weakest having small sample sizes and no measure of statistical significance (two).

Interventions

There was a range of intervention types. Eight of the interventions employed traditional education in the form of didactic teaching, sometimes supplemented with posters, flip-charts, and question-and-answer sessions (19,43,45,48,49,52,53,55). Two interventions employed a more formal lecture presentation and gave out some supporting literature(31,47). Two interventions used stories and video presentations (40,42) and two interventions distributed puberty books (4,50) without further teacher input. Three interventions facilitated learning through peer-education (9,44,56) and seven different interventions focussed on Menstrual Hygiene Management training: some demonstrated pad usage with a menstrual kit (10,20,42,51) or using a doll (41) and two instructed participants on the use of menstrual cups (46,57).

Aims of the Studies

The common aim of the studies was to evaluate the impact of a Menstrual Education Intervention on Menstrual Health for adolescent girls. A number of studies were more broadly about puberty

[Type here]

For peer review only

education (31,40,47,48,50,52) Several studies measured Menstrual Knowledge, Attitudes and Practices (KAP) (4,6,56,31,40,43,44,49–51,55). Nearly all of the studies used a pre-test, post-test model but one study used a post-test only model (40). A small number specifically focussed on Menstrual Hygiene Management and evaluated training on pad replacement or cup insertion (10,20,35,41,42,44,45,57,58)

Analysis

The quantitative and qualitative results are reported under four main themes: Menstrual Knowledge, Menstrual Attitudes, Menstrual Practices and Multi-component interventions.

Quantitative results

Menstrual Knowledge.

A meta-analysis was conducted on 11 studies which measured a change in menstrual knowledge following an intervention. A visual inspection of forest plots showed that all studies found a significant improvement in menstrual knowledge. Where studies reported the mean and standard deviation of a menstrual knowledge questionaire, we calculated the effect size using Cohen's d (table 2).

The average effect size of studies in this review was 3.44. Taking this as a medium effect size, we ranked them lowest – highest and suggest that <2 is low and >5 is high. Where we could not calculate an effect size, we have calculated % change in score. Due to the limited and heterogeneous nature of the data, we interpret the results only relative to the other studies in this review.

The effect size of those that distributed pamphlets and books was lowest at 0.33 (31), followed by those that showed videos 1.40 (40) and then lectures with question and answer sessions 2.13 (49) and 4.81 (55). Small group or peer-teaching was high at 5.337 and 10.044 respectively. Large effect sizes may occur due to small sample sizes.

For peer review only - http://bmjopen.bmj.com/site/about/guidelines.xhtml

[Type here]

Table 2 Effect size of interventions designed to improve menstrual knowledge

Study type and measure	First author and date of publication	Intervention	Number of questions / N	Reliability/ Cronbach's alpha (except where stated otherwise)	Sample size / n	Treatment	Mean / arbitrary units (except where stated otherwise)	Standard Deviation / Arbitrary units (except where stated otherwise)	Number of individuals scoring > 75% (good)	% change in number of individuals scoring >75%	Statistical test / p value	Effect size / Cohen's d /Rank and Impact
**Control and intervention pre-test, post test**	Blake et al 2018	Distribution of puberty education books in the local language	9	0.77	318	control	(Mean Difference = 0.18)	(Pooled SD = 1.4)	No data		Wald Chi-squared , <0.001	**0.79 Second Low**
					318	intervention	(MD = 1.06)	(Pooled SD = 1.52)				
	Jarrahi et al 2020	Small group and peer teaching	34	0.78	30	control pre	45.1	8.4	No data		Kruskal-Wallis test, <0.001	**small group 5.34 Eighth High**
					30	control post	52.2	1				
					30	intervention 1 pre (small group)	48.3	6.1				
					30	intervention 1 post	84.5	8.5				**Peer group 10.04 Nineth High**
					30	intervention 2 pre (peer)	44.1	1.7				
					30	intervention 2 post	93.3	5.7				

For peer review only

[Type here]

[Type here]

	Setyowati et al 2019	Booklet distriution	14	0.886	87	control pre	No data	No data	No data	No data	Chi-squared <0.001	(medium)
					87	control post			17.2			
					87	intervention pre			58.6	54.95		
					87	intervention post			90.8			
	Sharma et al 2015	Interactive Teaching programme led by School Nurses	15	test, retest r = 0.93	25	control pre	8.02	No data	No data		t-test <0.001	**4.48 Sixth Medium**
					25	control post	8.06					
					25	intervention pre	8.04					
					25	intervention post	12.6					
	Su et al 2016	Lecture, question and answer session	13	KR20= 0.64	56	control pre	5.5	2.54	No data		t-test <0.001	**2.13 Fifth Medium**
					56	control post	5.71	2.3				
					60	intervention pre	5.73	2.56				
					60	intervention post	10.22	1.92				
	Valizadeh et al 2017	Lecture, booklet and pamphlets	15	0.72	120	control pre	8.5	2.5	No data		general linear model <0.002	**0.33 First Low**
					120	control post	9.1	2.4				
					124	intervention pre	8.2	2				

[Type here]

					124	intervention post	9.8	1.8				
	Kheirollahi et al 2017	Lecture, question and answer session	23 (100 point scale)	0.8	76	control pre	52.58	6.58	No data		t-test <0.001	**4.81 Seventh Medium**
					76	control post	52.77	6.87				
					76	intervention pre	55.83	6.77				
					76	intervention post	86.36	7.11				
**Control and intervention post-test only**	Hurwitz et al 2017	Health education videos, using animation	27	0.72	40	control	11.27	3.73	No data		t-test <0.001	**1.40 Third Low**
					40	intervention	15.67	2.4				
**one group pre-test, post test**	Arasteh et al 2019	Group counselling and pamphlets	15	0.8	30	pre	6.8	3.32	No data		Chi-squared <0.001	**1.64 Fourth Low**
					30	post	11.3	2				
	Haque et al 2014	Training with Field manual by medical professionals	10	0.73	416	pre	No data	No data	51	61.57	chi-squared <0.05	(high)
					416	post			82.4			
	Chadawada et al 2017	Didactic teaching with posters, and videos	4	no data	250	pre			72.7	19.12	chi-squared <0.05	(low)
					250	post			86.6			

For peer review only

[Type here]

Menstrual Attitudes. Five studies measured Menstrual Attitudes (4,40,47,49,51).

Four interventions reported significantly different (p<0.05) attitude scores, and three of those provided pamphlets that addressed cultural restrictions(4,47,51). The other was an intervention on dysmenorrhea and self-care and included pamphlets with video and peer-sharing. Girls who had taken part had a significant increase in confidence and decrease (p<0.001) in 'bothersome' menstrual attitude (49). The only intervention which did not find a significant difference in attitude pre- and post-test involved puberty education videos shown to early adolescent boys and girls (40).

Menstrual Practice. An intervention that trained intellectually-disabled adolescents in an 18-item pad replacement skill set found that pre-training and post-training differences were statistically significant (p<0.001) (41). A feasibility trial into the use of the menstrual cup by school girls in Kenya (46) found that usage increased as time went on and culminated in 96% usage after nine months. There was also an increase in hygiene, with the menstrual cup reported as reducing the prevalence of STIs from 19.2% to 12.9% (p=0.018) (57).

Multi-component interventions

An education intervention in India was part of a bigger project that involved pad provision and improved sanitation in schools. After four years, compared to unimproved schools, school attrition had fallen from 11% to 6% (P<0.003) (53). The effect of menstrual education alone cannot be separated out.

Qualitative results

Menstrual Knowledge. All studies reported an increase in Menstrual Knowledge. Interventions that used peer education and group counselling (9,44) were as effective as those delivered by medical professionals (51).

Those interventions that had a degree of interactivity were more effective than those that only gave out information. Those that encouraged discussions found that they led to an increased willingness to talk about menstruation and a greater awareness of what is normal (9,48).

Only two interventions embedded menstrual education into the school curriculum (52,53). In Nepal, some schools had received the WASH in Schools programme (WinS) (59). However, the girls were highly critical of their teachers, especially male teachers. They complained that 'Teachers often got embarrassed, referred students to their textbook, and did not answer questions' (52).

Menstrual Attitudes. Most studies commented on an improved menstrual attitude and one noted a reduction in anxiety (48). More than one study noted an improvement in confidence in performing menstrual health care behaviour, such as requesting pain relief for dysmenorrhea (49). Some studies observed an increased confidence of girls to push back against cultural restrictions, or harmful practices (9,19,48,51,60).

Menstrual Practices. Skills are required to use pads and cups so that they are positioned correctly, are comfortable and don't leak. The cup feasibility trial in Kenya found that on-going training and support may be required to master the technique over a period of six months to one year (46,57). Education was also found to be an important component of skill acquisition in Uganda, where pad-provision accompanied by education was shown to be more effective than pad-provision alone (19).

Multi-component interventions

[Type here]

For peer review only - http://bmjopen.bmj.com/site/about/guidelines.xhtml

[Type here]

The MENISCUS intervention in Uganda attributed its success to the synergy of five combined elements; teacher training on puberty education, a drama skit, pad provision, pain relief provision, and Water, Sanitation and Hygiene (WASH) facility improvements (10).

Synthesis: The Logic Model

Guided by logic models developed for school-based interventions (61), we worked backwards from the higher order aim of good Menstrual Health to propose a chain of causal events.

The aim of good Menstrual Health is the distal outcome to the intervention. It is characterized by girls feeling empowered and having agency to make choices about their own bodies and lives. They can choose a suitable menstrual product to meet their individual needs. They track their menstrual cycle to be well-prepared so that they are not caught out at school and have to go home, and they engage as necessary with reproductive health services, without shame or stigma. Girls that have agency are able to control their menstruation and not the other way around. They can focus on their school work and reach their potential.

Preceding the distal outcome is the intermediate outcome; unrestricted mobility and participation. Girls should be able to carry out normal activities such as eating/drinking with the family, attending school and playing sport when they are menstruating. This requires confidence in their own ability to manage outside of the confines of the house, and determination to enter spaces from which they are traditionally excluded.

Below that is the proximal outcome; hygienic and comfortable menstruation management. Girls should be able to use suitable menstrual products. They should be able to use water and soap to clean away menstrual blood, and they should be able to practise self-care to relieve the symptoms of dysmenorrhea, such as yoga. If they need painkillers or a rest, they should be able to request them of parents and teachers without embarrassment.

Menstrual KAP underpins these outcomes. In a theory of change, girls require knowledge of the menstrual cycle to prepare products. They may need skills to use the products correctly to avoid the risk of discomfort, leaks or of contracting reproductive tract infections. They may need confidence to ask for products and services. They may need awareness of self-care practices. They should know what is normal and when to seek help.

Inputs and Outputs to the Logic Model

Menstrual Education and training are the inputs. The output is improved menstrual knowledge, attitude and practices (KAP). The outputs are linked to the outcomes.

LOGMOD

Figure 2 The Logic Model

The results of the review provide evidence that menstrual education improves the menstrual knowledge, attitude and practices (KAP) of young adolescent girls. It is suggested that increasing the menstrual KAP of girls increases their confidence to seek further knowledge and skills in a positive feedback loop (see fig 2). Menstrual Education is viewed as underpinning the logic model and is the first step to achieving Menstrual Health.

[Type here]

For peer review only - http://bmjopen.bmj.com/site/about/guidelines.xhtml

[Type here]

DISCUSSION

All twenty-four included studies that evaluated some form of menstrual education intervention reported that there was a measurable improvement in the Menstrual Knowledge, Attitude or Practices of young adolescent girls. Menstrual experience and need may be the motivators, as there was little improvement in the knowledge or attitude of very early adolescent girls or boys.

Most of the interventions were delivered in the school setting where it is relatively easy to reach the target group, although only two embedded the education into the school curriculum. In theory, schools should have good coverage and objectivity for delivery of this sensitive information at an appropriate time. However, teachers themselves may be ill-equipped to teach about menstruation without proper training (62).

A larger effect was gained with the more interactive interventions that included question and answer sessions. We suggest that this relates to the higher degree of participation, and concurs with current educational philosophy about the importance of active learning (63), based on constructivist theory (64,65). Gardner added to Dewey's early work on active learning when he described 'transformative' teaching. This involves using a range of methods that encourage the learner to find their own entry point and engage with the subject, often utilising space and creativity, and linking with their own experience(66). Discussing menstruation gives the girls agency to determine what it is that they need to know for themselves.

Those interventions that demonstrated skills and allowed for physical touch were also very effective. Other hygiene interventions that have been evaluated have pointed to the positive impact of a physical interaction with the tools of behaviour change (67).

A logic model has been constructed to frame the effect of menstrual education interventions on Menstrual Health. Menstrual Education is seen as underpinning all desirable outcomes and programmes can be optimised by including an education aspect. There was evidence that interventions were successful in normalising menstruation and improving menstruation management, which is the proximal outcome of the logic model. There was more limited evidence for an improvement in school attendance and mobility or a refusal to accept menstrual restrictions. These are intermediate outcomes and it might be expected to take some time to move from the proximal to the intermediate outcomes in the theory of change.

Multi-component interventions may be more successful than single components in achieving the distal outcome of Menstrual Health and Well-being. Girls need an enabling environment as well as knowledge. From a constructivist perspective which places learning within a social context(65,68) interventions that seek to improve the menstrual literacy of the whole community and reduce menstrual stigma may be more effective in achieving the macro-distal outcomes of girl empowerment and gender equality.

Implications for Policy and Practice

This review provides evidence that menstrual education has a positive effect on the menstrual knowledge, attitudes and practices of adolescent girls and needs to be delivered by trained personnel who are confident to lead discussion. Especially but not exclusively in LMIC, where resources are limited, it would be prudent to ensure that menstrual education is embedded into the school curriculum and that teachers receive specialist training.

[Type here]

For peer review only - http://bmjopen.bmj.com/site/about/guidelines.xhtml

[Type here]

Progress towards Menstrual Health is limited without an enabling environment. In order to achieve the more distal outcomes of the logic model, programme and policy makers need to address the menstrual literacy of the wider population. Multi-component interventions that speak to different actors and include hardware and software provision alongside menstrual education may make Menstrual Health more attainable.

Covid-19

This review was carried out on studies conducted before the Global Pandemic began in March 2020. The subsequent lockdown has had a profound effect on education, and many programmes have had to go on-line. We would encourage menstrual educators to be mindful of the benefits of interaction and make use of on-line teaching platforms that facilitate discussion in break-out rooms.

Limitations of this Review

The review was carried out in the English language, which may have missed some publications. Because menstrual health is an emerging topic with evolving terminology, search terms may not have adequately captured all currently used descriptors.

As a mixed methods review, there are a number of systemic limitations derived from comparing heterogeneous data sets. In particular, the studies did not measure the same outputs and the methodological quality of the studies was mixed. It is possible that the level of knowledge in some LMIC was so low at base-line that any educational intervention is an improvement.

Although all interventions reported positive outcomes, this may be due to publication bias, where only significant results are shared. Cohen's d has not previously been calculated for this discipline and therefore the magnitude of the effect size can only be considered relative to others in this review.

The number of studies was small, and only one study was from a High Income Country, so it is difficult to say how applicable the conclusions are to a HIC. More research needs to be done in this area, particularly as period poverty has been increasingly reported since the start of the pandemic in HIC.

REGISTRATION

For this review, a protocol was not prepared or registered.

REFERENCES

[Type here]

For peer review only

1. Chandra-Mouli V, Patel S V. Mapping the knowledge and understanding of menarche, menstrual hygiene and menstrual health among adolescent girls in low- and middle-income countries. Reprod Health. 2017;14.

2. Marván ML, Alcalá-Herrera V. Age at Menarche, Reactions to Menarche and Attitudes towards Menstruation among Mexican Adolescent Girls. J Pediatr Adolesc Gynecol. 2014;27(2):61–6.

3. Mason L, Nyothach E, Alexander K, Odhiambo FO, Eleveld A, Vulule J, et al. "We keep it secret so no one should know" - A qualitative study to explore young schoolgirls attitudes and experiences with menstruation in rural Western Kenya. PLoS One. 2013;8(11).

4. Setyowati S, Rizkia M, Ungsianik T. Improving Female Adolescents' Knowledge, Emotional Response, and Attitude toward Menarche following Implementation of Menarcheal Preparation Reproductive Health Education. Asian/Pacific Isl Nurs J. 2019;4(2):84–91.

5. Shah V, Nabwera HM, Sosseh F, Jallow Y, Comma E, Keita O. A rite of passage : a mixed methodology study about knowledge , perceptions and practices of menstrual hygiene management in rural Gambia. BMC Public Health. 2019;19(277):1–15.

6. Sharma R, Sandhya N, Deepika K, Varsha S, Vardha. Menstrual hygiene among adolescent girls. Indian J Community Heal. 2015;27(3):376–80.

7. Marván ML, Alcalá-Herrera V. Age at menarche, reactions to menarche and attitudes towards menstruation among Mexican adolescent girls. J Pediatr Adolesc Gynecol. 2014;27(2):61–6.

8. Coast E, Lattof SR, Strong J. Puberty and menstruation knowledge among young adolescents in low- and middle-income countries: a scoping review. Vol. 64, International Journal of Public Health. Springer International Publishing; 2019. p. 293–304.

9. Ramaiya A, Malhotra A, Cronin C, Stevens S, Kostizak K, Sharma A, et al. How does a Social and Behavioral Change Communication Intervention Predict Menstrual Health and Hygiene Management: A Cross-Sectional Study. BMC Public Health. 2019;19(1):1–12.

10. Nalugya R, Tanton C, Hytti L, Kansiime C, Nakuya K, Namirembe P, et al. Assessing the effectiveness of a comprehensive menstrual health intervention program in Ugandan schools (MENISCUS): process evaluation of a pilot intervention study. Pilot Feasibility Stud. 2020;6(1):1–15.

11. House S, Mahon T, Cavill S. Menstrual Hygiene Matters: a resource for improving menstrual hygiene around the world. Reprod Health Matters. 2013 Jan;21(41):257–9.

12. Hennegan JM. Menstrual Hygiene Management and Human Rights: The Case for an Evidence-Based Approach. Women's Reprod Heal. 2017;

13. Ellis A, Haver J, Villasenor J, Parawan A, Venkatesh M, Freeman MC, et al. WASH challenges to girls' menstrual hygiene management in Metro Manila, Masbate, and South Central Mindanao, Philippines. Waterlines. 2016;

14. Rani A, Sharma MK, Singh A. Practices and perceptions of adolescent girls regarding the impact of dysmenorrhea on their routine life: A comparative study in the urban, rural, and slum areas of Chandigarh. Int J Adolesc Med Health. 2016;28(1):3–9.

15. Sumpter C, Torondel B. A Systematic Review of the Health and Social Effects of Menstrual Hygiene Management. PLoS One. 2013;8(4).

16. Tiwari A, Ekka IJ, Thakur R. Assessment of knowledge and practices regarding menstrual hygiene among adolescent girls of Government higher secondary school, station Murhipar,

For peer review only - http://bmjopen.bmj.com/site/about/guidelines.xhtml

[Type here]

Rajnandgaon (C.G.). Int J Community Med Public Heal. 2018;5(4):1335–8.

17. Sommer M, Kjellén M, Pensulo C. Girls' and women's unmet needs for menstrual hygiene management (MHM): The interactions between MHM and sanitation systems in low-income countries. Vol. 3, Journal of Water Sanitation and Hygiene for Development. 2013. p. 283–97.

18. Hennegan J. Inserting informed choice into global menstrual product use and provision. Lancet Public Heal. 2019;4(8):e361–2.

19. Hennegan J, Dolan C, Steinfield L, Montgomery P. A qualitative understanding of the effects of reusable sanitary pads and puberty education: implications for future research and practice. Reprod Health. 2017;14.

20. Kansiime C, Hytti L, Nalugya R, Nakuya K, Namirembe P, Nakalema S, et al. Menstrual health intervention and school attendance in Uganda (MENISCUS-2): A pilot intervention study. BMJ Open. 2020;10(2):1–11.

21. Sebert Kuhlmann A, Peters Bergquist E, Danjoint D, Wall LL. Unmet Menstrual Hygiene Needs Among Low-Income Women. Obstet Gynecol. 2019;133(2):238–44.

22. PHS. Lifting the lid on period poverty. 2019.

23. Whitehead S, Biddle S. Adolescent girls' perceptions of physical activity: A focus group study. Eur Phys Educ Rev. 2008;14(2):243–62.

24. Biddle SJH, Whitehead SH, O'Donovan TM, Nevill ME. Correlates of participation in physical activity for adolescent girls: A systematic review of recent literature. J Phys Act Heal. 2005;2(4):423–34.

25. Gultie T, Hailu D, Workineh Y. Age of Menarche and Knowledge about Menstrual Hygiene Management among Adolescent School Girls in Amhara Province, Ethiopia: Implication to Health Care Workers & School Teachers. PLoS One. 2014;9(9).

26. Al Omari O, Razeq NMA, Fooladi MM. Experience of Menarche Among Jordanian Adolescent Girls: An Interpretive Phenomenological Analysis. J Pediatr Adolesc Gynecol. 2016;29(3):246–51.

27. Raskova M, Stolinska DP. COMMUNICATION ABOUT PUBERTY AMONG MIDDLE-SCHOOL-AGED CHILDREN. In: Bekirogullari Z, Minas MY, Thambusamy RX, editors. International Conference on Education and Educational Psychology. 2017. p. 194–202.

28. Bennett C, Harden J. An exploration of mothers' attitudes towards their daughters' menarche. Sex Educ Soc Learn. 2014;14(4):457–70.

29. Afsari A. Predictors of knowledge of puberty health among girl students. Biosci Biotechnol Res Commun. 2017;10(2):264–9.

30. Sooki Z, Shariati M, Chaman R, Khosravi A, Effatpanah M, Keramat A. The Role of Mother in Informing Girls About Puberty: A Meta-Analysis Study. Nurs Midwifery Stud. 2016;Inpress(Inpress):1–10.

31. Valizadeh S, Assdollahi M, Mirghafourvand M, Afsari A. Educating Mothers and Girls about Knowledge and Practices toward Puberty Hygiene in Tabriz, Iran: A Randomized Controlled Clinical Trial. Iran Red Crescent Med J. 2017;19(2).

32. Tingle, C & Vora S. Break the Barriers : Girls ' Experiences of. Plan Int UK. 2018;1–68.

33. Collier-Harris CA, Goldman JDG. Is puberty education evident in Australia's first national

[Type here]

curriculum? Sex Educ Soc Learn. 2017;17(1):57–72.

34. Sumpter C, Torondel B. A Systematic Review of the Health and Social Effects of Menstrual Hygiene Management. PLoS One. 2013;8(4).

35. Hennegan J, Montgomery P. Do menstrual hygiene management interventions improve education and psychosocial outcomes for women and girls in low and middle income countries? A systematic review. PLoS One. 2016;11(2):1–21.

36. Gignac GE, Szodorai ET. Effect size guidelines for individual differences researchers. Pers Individ Dif. 2016;102(December):74–8.

37. Hemphill JF. Interpreting the Magnitudes of Correlation Coefficients. Am Psychol. 2003;58(1):78–9.

38. Bakker A, Cai J, English L, Kaiser G, Mesa V, Van Dooren W. Beyond small, medium, or large: points of consideration when interpreting effect sizes. Educ Stud Math. 2019;102(1):1–8.

39. Thomas J, Harden A. Methods for the thematic synthesis of qualitative research in systematic reviews. BMC Med Res Methodol. 2008;8(45):1–10.

40. Hurwitz LB, Lovato SB, Lauricella AR, Woodruff TK, Patrick E, Wartella E. "A New You, That's Who": an evaluation of short videos on puberty and human reproduction. Palgrave Commun. 2018;4(1).

41. Altundağ S, Çalbayram NÇ. Teaching menstrual care skills to intellectually disabled female students. Mol Ecol. 2016;25(13–14):1962–8.

42. Ariyanti TD, Royanto LRM. The Effectiveness of Social Stories and Video Modeling in Improving Self-Care Skills in Female Adolescents with Mild Intellectual Disabilities during Menstrual Periods. 2018;135(Iciap 2017):189–99.

43. Rani Chadalawada U, Devi S A, Rani M S. Effect of Health Education on Adolescent Girls Regarding Knowledge About Menstruation. J Evol Med Dent Sci. 2017;6(13):1040–3.

44. Jarrahi R, Golmakani N, Mazlom SR. Effect of menstrual hygiene education based on peer and small group teaching methods on hygiene behaviors in female adolescents: A comparative study. Evid Based Care J. 2020;10(1):70–4.

45. Montgomery P, Hennegan J, Dolan C, Wu M, Steinfield L, Scott L. Menstruation and the Cycle of Poverty: A Cluster Quasi-Randomised Control Trial of Sanitary Pad and Puberty Education Provision in Uganda. PLoS One. 2016;11(12).

46. Van Eijk AM, Laserson KF, Nyothach E, Oruko K, Omoto J, Mason L, et al. Use of menstrual cups among school girls: Longitudinal observations nested in a randomised controlled feasibility study in rural western Kenya. Reprod Health. 2018;15(1):1–11.

47. Afsari A, Mirghafourvand M, Valizadeh S, Abbasnezhadeh M, Galshi M, Fatahi S. The effects of educating mothers and girls on the girls' attitudes toward puberty health: A randomized controlled trial. Int J Adolesc Med Health. 2015;2015.

48. Mokari H, Khaleghparast S, Samani LN. Impact of Puberty Health Education on Anxiety of Adolescents. Int J Med Res Heal Sci. 2016;5(5, S, SI):284–91.

49. Su JJ, Lindell D. Promoting the menstrual health of adolescent girls in China. Nurs Heal Sci. 2016;18(4):481–7.

50. Blake S, Boone M, Yenew Kassa A, Sommer M. Teaching Girls About Puberty and Menstrual

For peer review only - http://bmjopen.bmj.com/site/about/guidelines.xhtml

Hygiene Management in Rural Ethiopia: Findings From a Pilot Evaluation. J Adolesc Res. 2018;33(5):623–46.

51. Haque SE, Rahman M, Itsuko K, Mutahara M, Sakisaka K. The effect of a school-based educational intervention on menstrual health: An intervention study among adolescent girls in Bangladesh. BMJ Open. 2014;4(7):1–9.

52. Morrison J, Basnet M, Bhatt A, Khimbanjar S, Chaulagain S, Sah N, et al. Girls' menstrual management in five districts of Nepal: Implications for policy and practice. Stud Soc Justice. 2018;12(2):251–72.

53. Sivakami M, van Eijk AM, Thakur H, Kakade N, Patil C, Shinde S, et al. Effect of menstruation on girls and their schooling, and facilitators of menstrual hygiene management in schools: Surveys in government schools in three states in India, 2015. J Glob Health. 2019;9(1).

54. Hutchison D, Styles B. A guide to running randomised controlled trials for educational researchers. Natl Found Educ Res. 2010;1–65.

55. Kheirollahi F, Rahimi Z, Arsang-Jang S, Sharifirad G, Sarraf P, Gharlipour Z. Puberty health status among adolescent girls: A model- based educational program. Int J Pediatr. 2017;5(7):5369–78.

56. Arasteh FE, Shobeiri F, Parsa P, Mohamadi Y. Effect of group counseling on adolescent girls in improving knowledge and practice of menstrual hygiene in welfare boarding centers. J Postgrad Med Inst. 2019;33(1):46–51.

57. Phillips-Howard PA, Nyothach E, Ter Kuile FO, Omoto J, Wang D, Zeh C, et al. Menstrual cups and sanitary pads to reduce school attrition, and sexually transmitted and reproductive tract infections: A cluster randomised controlled feasibility study in rural Western Kenya. BMJ Open. 2016;

58. Van Eijk AM, Sivakami M, Thakkar MB, Bauman A, Laserson KF, Coates S, et al. Menstrual hygiene management among adolescent girls in India: A Systematic review and meta-analysis. BMJ Open. 2016;6(3).

59. United Nations Children's Fund (UNICEF) and World Health Organization 2018. Drinking Water, Sanitation and Hygiene in Schools : Global baseline report 2018. Unicef. 2018. 82 p.

60. Bhagwat A, Jijina P. A Psychosocial Lens on an Indigenous Initiative to Address Menstrual Health and Hygiene in Indian Villages. Soc Work Public Health. 2020;35(3):73–89.

61. Harris K, Kneale D, Tj L, Vm M, Grigg J, Thomas J, et al. School-based self-management interventions for asthma in children and adolescents : a mixed methods systematic review (Review). 2019;

62. DfE. Relationships Education, Relationships and Sex Education (RSE) and Health Education. 2019;(November):1–6.

63. Freeman S, Eddy SL, McDonough M, Smith MK, Okoroafor N, Jordt H, et al. Active learning increases student performance in science, engineering, and mathematics. Proc Natl Acad Sci U S A. 2014;111(23):8410–5.

64. Dewey J. Experience and Education [1938]. 1938;

65. Vygotsky L. Interaction between learning and development. Readings Dev Child. 1978;23(3):34–41.

66. Achkovska Leshkovska E, Miovska Spaseva S. John Dewey'S educational theory and

For peer review only - http://bmjopen.bmj.com/site/about/guidelines.xhtml

[Type here]

educational implications of Howard Gardner's multiple intelligences theory. Int J Cogn Res Sci Eng Educ. 2016;4(2):57–66.

67. Yilmaz SK, Bohara AK, Chowdhury SR. Touch for Health: Use of Pavlovian Processes with Physical Touch as a Means to Improve Menstrual Hygiene Management Initiatives, Measured by Willingness to Pay. PharmacoEconomics - Open. 2020;4(2):263–76.

68. Lock A, Strong T, editors. Introduction. In: Social Constructionism: Sources and Stirrings in Theory and Practice. Cambridge: Cambridge University Press; 2010. p. 1–11.

ADDITIONAL INFORMATION

Contributors

RE, FG and BH designed the review. RE and OC reviewed titles, abstracts and full-texts for eligibility. Disagreement was resolved by discussion and where necessary FG and BH offered their view. RE, FG and BH agreed on a data extraction framework, which was then carried out by RE. The quality assessment tool was agreed upon by RE, FG and BH. RE used the MMAT to assess the quality and this was verified by FG and BH. RE prepared the manuscript and it was reviewed and edited by FG and BH.

Corresponding author

Rebecca.R.Evans@warwick.ac.uk

Funding Statement

This work was supported by the National Institute for Health Research Unit on Improving Health in Slums. Funder reference: 16/136/87.

Ethical Approval

BSREC 43/19-20

Competing Interests

None declared

Patient Consent

None required

Data Sharing Statement

No original data were generated in this study

Word Count 4186

For peer review only

For peer review only - http://bmjopen.bmj.com/site/about/guidelines.xhtml

[Type here]

[Type here]

For peer review only

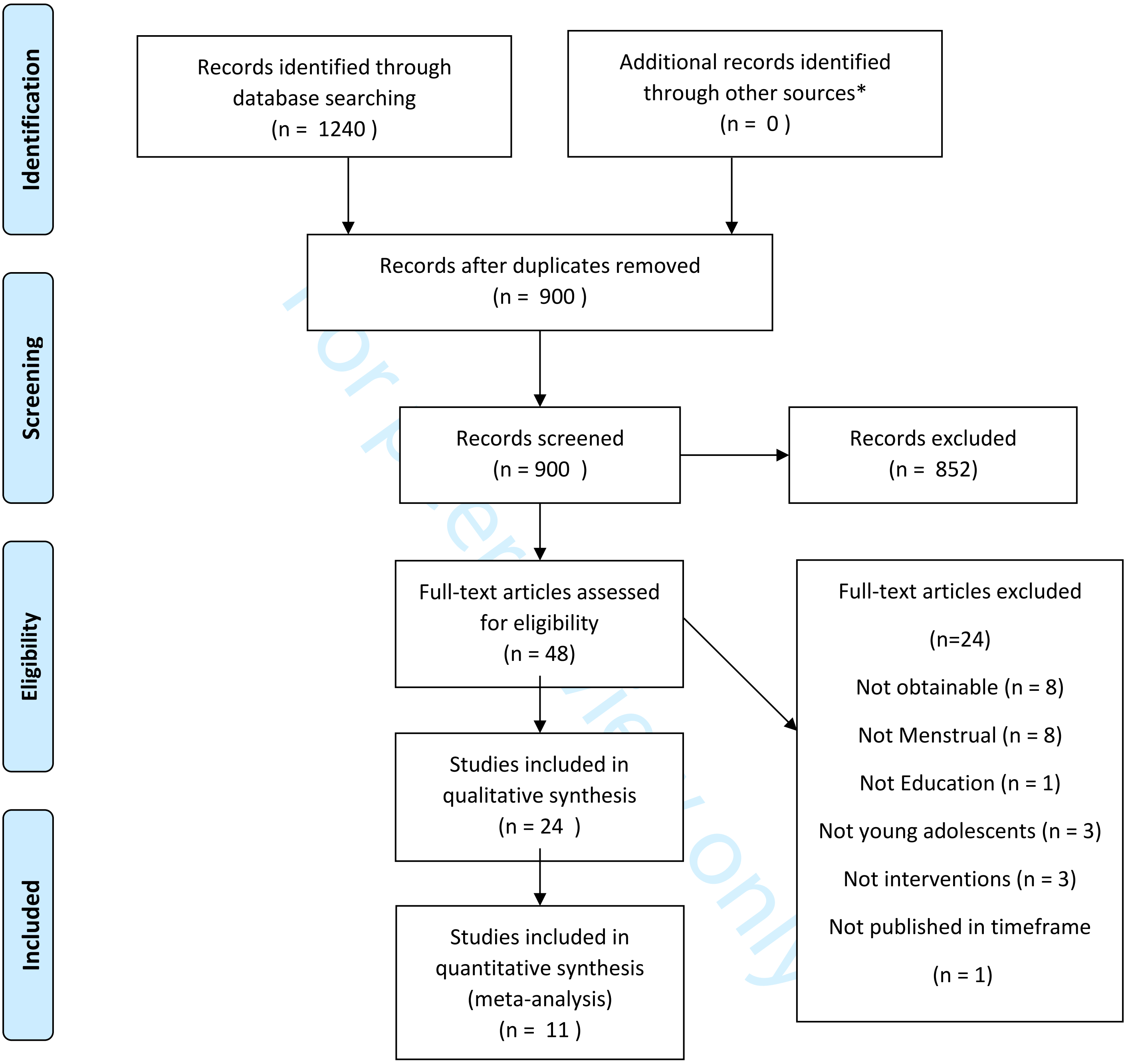

Figure 2. PRISMA flow chart

*grey literature

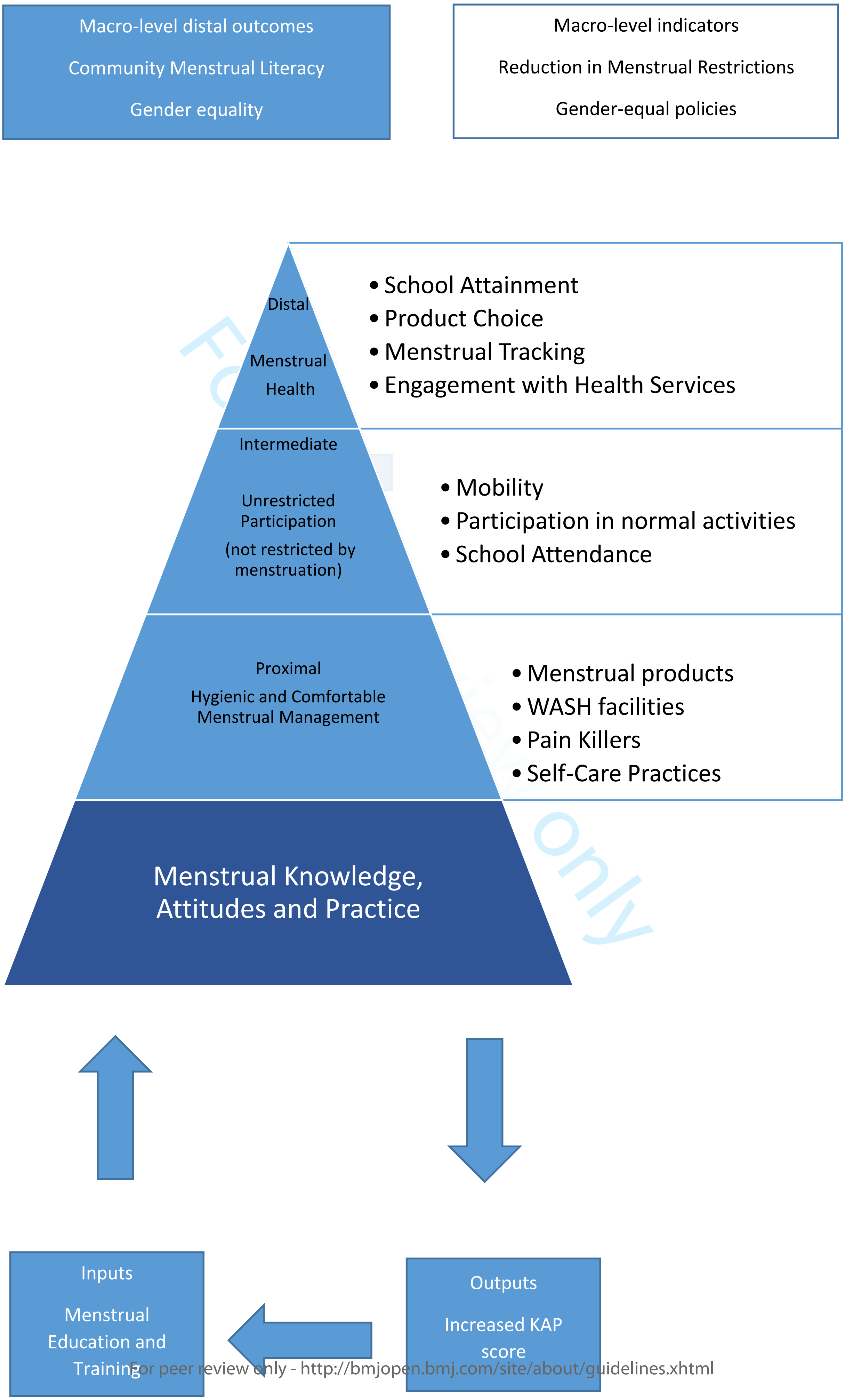
Macro-level distal outcomes
Community Menstrual Literacy
Gender equality
Macro-level indicators
Reduction in Menstrual Restrictions
Gender-equal policies
Distal
Menstrual Health
• School Attainment
• Product Choice
• Menstrual Tracking
• Engagement with Health Services
Intermediate
Unrestricted Participation
(not restricted by menstruation)
• Mobility
• Participation in normal activities
• School Attendance
Proximal
Hygienic and Comfortable Menstrual Management
• Menstrual products
• WASH facilities
• Pain Killers
• Self-Care Practices
Menstrual Knowledge, Attitudes and Practice
Inputs
Menstrual Education and Training
Outputs
Increased KAP score

For peer review only

Supplementary File: Characteristics and quality of included studies

Citation	Design	Country	Study population	Sampling Method	No of participants	Type of intervention	MMAT Clear Research Questions /2	MMAT Appropriate Data Collection?/2	MMAT Supplementary questions relevant to methodology/5	MMAT Total/9	MMAT RAG rating
(1)	RCT quasi-experimental pre-test, post test	Iran	Mothers and adolescent school girls	Random Cluster-sampling	364	Half hour lecture and pamphlet distribution to improve attitude	2	1	5	8	Green
(2)	Quantitative Descriptive Case Series Quasi-experimental pre-test, post-test	Turkey	Intellectually-disabled girls in special education training centre	convenience	77	Demonstration on doll to improve Pad-replacement skills	2	2	4	8	Green
(3)	Quantitative descriptive one group pre-test, post-test	Iran	All adolescent girls in two welfare	Purposive sampling	30	Group counselling in boarding centres	2	2	3	7	Amber

			boarding centres								
(4)	Quantitative descriptive case report; pre-test, post-test	Indonesia	Mild-intellectually disabled girl	Purposive sampling	1	Stories and video-modelling	2	2	3	7	Amber
(5)	Mixed methods, quantitative survey pre-test, post-test	Ethiopia	Girls enrolled in school in grades 6 and 7	Cluster randomised approach	636	Puberty Book 'Growth and Changes'	2	2	5	9	Green
(6)	RCT, pre-test, post-test	India	Adolescent high school girls aged 11 – 19 years of Zilla Parishad High School	Systematic random technique (every other girl on register)	250	Chalk and talk, posters, flipcharts,	1	2	2	5	Red
(7)	Quantitative descriptive one group, Pre-test, post-test	Bangladesh	adolescent female students aged 11–16 years, in grade 6–8, and living with their parents.	Random cluster sampling	416	Field manual training	2	2	3	7	Amber
(8)	Qualitative understanding of quasi-RCT	Uganda	school girls in eight study schools in primary classses grades 4 - 7.	Quasi-randomised cluster sampling	27: education (n=8) pads alone (n=8) education and pads (n=6) and	75 minute session of Straight Talk Education programme	2	2	5	9	Green

					control (n=5)						
(9)	RCT pre-test post-test	USA	Children of both sexes whose parents had registered an interest	Random sampling	80 (43 girls)	'A New You, that's Who' video series	2	2	3	7	Amber
(10)	3 arm RCT Control and 2 intervention: peer and small group teaching pre-test/post-test	Iran	Adolescent school girls	Random sampling of three high schools	90	Small group and peer-teaching	2	2	3	7	Amber
(11)	Mixed methods Longitudinal study	Uganda	Girls and boys in secondary school	Purposive selection of schools	369	Multi-component approach to optimising government guidelines for puberty education	2	2	5	9	Green
(12)	Quasi-experimental RCT, and pre-test, post- test	Iran	School girls	Random cluster sampling	152	A model-based educational program	1	2	2	5	Red
(13)	Quasi-experimental RCT	Iran	School girls aged 14 – 16 years and their parents	Random cluster sampling	159	Teaching programme with discussion	2	2	3	7	Amber
(14)	four-armed quasi-experimental RCT Pre and post test	Uganda	Girls in grades 3 - 5	Randomised	1124	Educational arm used Straight Talk	1	2	0	3	Amber

For peer review only

(15)	Mixed Methods Quantitative cross-sectional surveys and qualitative focus group	Nepal	Girls in grades seven to ten in 28 large schools.	Random cluster sampling	860	WASH in Schools (WinS) programme	1	2	4	7	Amber
(16)	Mixed methods process evaluation Longitudinal study	Uganda	Girls and boys in secondary school	Purposive selection of schools	369	Multi-component approach to optimising government guidelines for puberty education	2	2	5	9	Green
(17)	3-arm single-site open cluster randomised controlled pilot study.	Kenya	Primary girls aged 14 -16	Random cluster sampling	644	Puberty education and menstrual cup instruction from nurse	1	2	5	8	Green
(18)	Quantitative non-random post-test only	India	Adolescent girls	Purposive (schools) and random (girls)	2206	Monthly discussion group	2	2	5	9	Green
(19)	Quasi-experimental Control/intervention pre-test post-test	Indonesia	Girls aged 9-12 pre-menarch	Stratified random sampling technique	174	Booklet	2	2	2	6	Amber
(20)	Experimental; Intervention and control; pre-test, post-test	India	Adolescent girls at a government high school	Randomly sampled	50	One training session on menstrual hygiene	1	0	1	2	Red
(21)	Quantitative descriptive Pre-test, post-test	India	Adolescent girls	Multi-level stratified	2564	'model' schools received	1	2	5	8	Green

				sampling of schools		additional WASH support and education embedded into curriculum					
(22)	A quasi-experimental RCT two group pretest-posttest design	China	Adolescent girls	Purposive sampling of schools	116	Researcher – led Menstrual Hygiene Class	2	2	4	8	Green
(23)	Mixed methods. Qualitative review and semi-quantitative measure of cup use nested in larger RCT	Kenya	Post-menarche adolescent girls	Random allocation of 10 schools to menstrual cup arm	192	Puberty education and menstrual cup instruction from nurse	1	2	5	8	Green
(24)	RCT three arm – educating girls, educating mothers or control. pre and post test	Iran	Post-menarche adolescent school girls	12 purposively sampled schools	327	30 minute lecture on puberty hygiene plus a booklet	0	1	5	6	Amber

Summary of characteristics of included studies

Framework analysis and the 'best fit' principle were used to score the studies (25–27). All studies were interrogated with two questions 'Are there clear research questions?' and 'Do the collected data allow the research questions to be addressed?' which were considered fundamental to the quality and were scored on a scale of 'Yes' = 2, 'not clear' = 1 and 'No' = 0. Five further supplementary questions were considered that addressed quality issues such as sample size. The sets of questions were different depending upon the study design, and are not directly comparable, so less weight was given to these; Yes = 1 and No = 0. The maximum score when added together was 2 + 2 + 5 = 9. Studies scored 0-5 were categorised as low quality (as it was possible to get these scores without clear research questions or valid methods); those that scored 6 or 7 were scored as moderate quality and those that scored 8 or 9 were scored as high quality (a subjective scale based on personal expertise (27) and community of practice validation (28).

REFERENCES

For peer review only

1. Afsari A, Mirghafourvand M, Valizadeh S, Abbasnezhadeh M, Galshi M, Fatahi S. The effects of educating mothers and girls on the girls' attitudes toward puberty health: A randomized controlled trial. Int J Adolesc Med Health. 2015;2015.

2. Altundağ S, Çalbayram NÇ. Teaching menstrual care skills to intellectually disabled female students. Mol Ecol. 2016;25(13–14):1962–8.

3. Arasteh FE, Shobeiri F, Parsa P, Mohamadi Y. Effect of group counseling on adolescent girls in improving knowledge and practice of menstrual hygiene in welfare boarding centers. J Postgrad Med Inst. 2019;33(1):46–51.

4. Ariyanti TD, Royanto LRM. The Effectiveness of Social Stories and Video Modeling in Improving Self-Care Skills in Female Adolescents with Mild Intellectual Disabilities during Menstrual Periods. 2018;135(Iciap 2017):189–99.

5. Blake S, Boone M, Yenew Kassa A, Sommer M. Teaching Girls About Puberty and Menstrual Hygiene Management in Rural Ethiopia: Findings From a Pilot Evaluation. J Adolesc Res. 2018;33(5):623–46.

6. Rani Chadalawada U, Devi S A, Rani M S. Effect of Health Education on Adolescent Girls Regarding Knowledge About Menstruation. J Evol Med Dent Sci. 2017;6(13):1040–3.

7. Haque SE, Rahman M, Itsuko K, Mutahara M, Sakisaka K. The effect of a school-based educational intervention on menstrual health: An intervention study among adolescent girls in Bangladesh. BMJ Open. 2014;4(7):1–9.

8. Hennegan J, Montgomery P. Do menstrual hygiene management interventions improve education and psychosocial outcomes for women and girls in low and middle income countries? A systematic review. PLoS One. 2016;11(2):1–22.

9. Hurwitz LB, Lovato SB, Lauricella AR, Woodruff TK, Patrick E, Wartella E. "A New You, That's Who": an evaluation of short videos on puberty and human reproduction. Palgrave Commun. 2018;4(1).

10. Jarrahi R, Golmakani N, Mazlom SR. Effect of menstrual hygiene education based on peer and small group teaching methods on hygiene behaviors in female adolescents: A comparative study. Evid Based Care J. 2020;10(1):70–4.

11. Kansiime C, Hytti L, Nalugya R, Nakuya K, Namirembe P, Nakalema S, et al. Menstrual health intervention and school attendance in Uganda (MENISCUS-2): A pilot intervention study. BMJ Open. 2020;10(2):1–11.

12. Kheirollahi F, Rahimi Z, Arsang-Jang S, Sharifirad G, Sarraf P, Gharlipour Z. Puberty health status among adolescent girls: A model- based educational program. Int J Pediatr. 2017;5(7):5369–78.

13. Mokari H, Khaleghparast S, Samani LN. Impact of Puberty Health Education on Anxiety of Adolescents. Int J Med Res Heal Sci. 2016;5(5, S, SI):284–91.

14. Montgomery P, Hennegan J, Dolan C, Wu M, Steinfield L, Scott L. Menstruation and the Cycle of Poverty: A Cluster Quasi-Randomised Control Trial of Sanitary Pad and Puberty Education Provision in Uganda. PLoS One. 2016;11(12).

15. Morrison J, Basnet M, Bhatt A, Khimbanjar S, Chaulagain S, Sah N, et al. Girls' menstrual management in five districts of Nepal: Implications for policy and practice. Stud Soc Justice. 2018;12(2):251–72.

16. Nalugya R, Tanton C, Hytti L, Kansiime C, Nakuya K, Namirembe P, et al. Assessing the effectiveness of a comprehensive menstrual health intervention program in Ugandan schools (MENISCUS): process evaluation of a pilot intervention study. Pilot Feasibility Stud. 2020;6(1):1–15.

17. Phillips-Howard PA, Nyothach E, Ter Kuile FO, Omoto J, Wang D, Zeh C, et al. Menstrual cups and sanitary pads to reduce school attrition, and sexually transmitted and reproductive tract infections: A cluster randomised controlled feasibility study in rural Western Kenya. BMJ Open. 2016;

18. Ramaiya A, Malhotra A, Cronin C, Stevens S, Kostizak K, Sharma A, et al. How does a Social and Behavioral Change Communication Intervention Predict Menstrual Health and Hygiene Management: A Cross-Sectional Study. BMC Public Health. 2019;19(1):1–12.

19. Setyowati S, Rizkia M, Ungsianik T. Improving Female Adolescents' Knowledge, Emotional Response, and Attitude toward Menarche following Implementation of Menarcheal Preparation Reproductive Health Education. Asian/Pacific Isl Nurs J. 2019;4(2):84–91.

20. Sharma R, Sandhya N, Deepika K, Varsha S, Vardha. Menstrual hygiene among adolescent girls. Indian J Community Heal. 2015;27(3):376–80.

21. Sivakami M, van Eijk AM, Thakur H, Kakade N, Patil C, Shinde S, et al. Effect of menstruation on girls and their schooling, and facilitators of menstrual hygiene management in schools: Surveys in government schools in three states in India, 2015. J Glob Health. 2019;9(1).

22. Su JJ, Lindell D. Promoting the menstrual health of adolescent girls in China. Nurs Heal Sci. 2016;18(4):481–7.

23. Van Eijk AM, Sivakami M, Thakkar MB, Bauman A, Laserson KF, Coates S, et al. Menstrual hygiene management among adolescent girls in India: A Systematic review and meta-analysis. BMJ Open. 2016;6(3).

24. Valizadeh S, Assdollahi M, Mirghafourvand M, Afsari A. Educating Mothers and Girls about Knowledge and Practices toward Puberty Hygiene in Tabriz, Iran: A Randomized Controlled Clinical Trial. Iran Red Crescent Med J. 2017;19(2).

25. Carroll C, Booth A, Cooper K. A worked example of "best fit" framework synthesis: A systematic review of views concerning the taking of some potential chemopreventive agents. BMC Med Res Methodol. 2011;11.

26. Carroll C, Booth A, Leaviss J, Rick J. "best fit" framework synthesis: Refining the method. BMC Med Res Methodol. 2013;13(1).

27. Suto I. A critical review of some qualitative research methods used to explore rater cognition. Educ Meas Issues Pract. 2012;31(3):21–30.

28. Bejar II. A validity-based approach to quality control and assurance of automated scoring. Assess Educ Princ Policy Pract. 2011;18(3):319–41.

For peer review only

For peer review only

PRISMA 2020 Checklist

Section and Topic	Item #	Checklist item	Location where item is reported
**TITLE**			
Title	1	Identify the report as a systematic review.	1
**ABSTRACT**			
Abstract	2	See the PRISMA 2020 for Abstracts checklist.	
**INTRODUCTION**			
Rationale	3	Describe the rationale for the review in the context of existing knowledge.	3-4
Objectives	4	Provide an explicit statement of the objective(s) or question(s) the review addresses.	4
**METHODS**			
Eligibility criteria	5	Specify the inclusion and exclusion criteria for the review and how studies were grouped for the syntheses.	4
Information sources	6	Specify all databases, registers, websites, organisations, reference lists and other sources searched or consulted to identify studies. Specify the date when each source was last searched or consulted.	5
Search strategy	7	Present the full search strategies for all databases, registers and websites, including any filters and limits used.	5
Selection process	8	Specify the methods used to decide whether a study met the inclusion criteria of the review, including how many reviewers screened each record and each report retrieved, whether they worked independently, and if applicable, details of automation tools used in the process.	5
Data collection process	9	Specify the methods used to collect data from reports, including how many reviewers collected data from each report, whether they worked independently, any processes for obtaining or confirming data from study investigators, and if applicable, details of automation tools used in the process.	6
Data items	10a	List and define all outcomes for which data were sought. Specify whether all results that were compatible with each outcome domain in each study were sought (e.g. for all measures, time points, analyses), and if not, the methods used to decide which results to collect.	6
	10b	List and define all other variables for which data were sought (e.g. participant and intervention characteristics, funding sources). Describe any assumptions made about any missing or unclear information.	6
Study risk of bias assessment	11	Specify the methods used to assess risk of bias in the included studies, including details of the tool(s) used, how many reviewers assessed each study and whether they worked independently, and if applicable, details of automation tools used in the process.	6
Effect measures	12	Specify for each outcome the effect measure(s) (e.g. risk ratio, mean difference) used in the synthesis or presentation of results.	6-7
Synthesis methods	13a	Describe the processes used to decide which studies were eligible for each synthesis (e.g. tabulating the study intervention characteristics and comparing against the planned groups for each synthesis (item #5)).	7
	13b	Describe any methods required to prepare the data for presentation or synthesis, such as handling of missing summary statistics, or data conversions.	7
	13c	Describe any methods used to tabulate or visually display results of individual studies and syntheses.	7
	13d	Describe any methods used to synthesize results and provide a rationale for the choice(s). If meta-analysis was performed, describe the model(s), method(s) to identify the presence and extent of statistical heterogeneity, and software package(s) used.	7
	13e	Describe any methods used to explore possible causes of heterogeneity among study results (e.g. subgroup analysis, meta-regression).	
	13f	Describe any sensitivity analyses conducted to assess robustness of the synthesized results.	
Reporting bias assessment	14	Describe any methods used to assess risk of bias due to missing results in a synthesis (arising from reporting biases).	7
Certainty	15	Describe any methods used to assess certainty (or confidence) in the body of evidence for an outcome.	7

For peer review only

For peer review only - http://bmjopen.bmj.com/site/about/guidelines.xhtml

PRISMA 2020 Checklist

Section and Topic	Item #	Checklist item	Location where item is reported
assessment			
**RESULTS**			
Study selection	16a	Describe the results of the search and selection process, from the number of records identified in the search to the number of studies included in the review, ideally using a flow diagram.	8
	16b	Cite studies that might appear to meet the inclusion criteria, but which were excluded, and explain why they were excluded.	
Study characteristics	17	Cite each included study and present its characteristics.	9
Risk of bias in studies	18	Present assessments of risk of bias for each included study.	SF
Results of individual studies	19	For all outcomes, present, for each study: (a) summary statistics for each group (where appropriate) and (b) an effect estimate and its precision (e.g. confidence/credible interval), ideally using structured tables or plots.	12
Results of syntheses	20a	For each synthesis, briefly summarise the characteristics and risk of bias among contributing studies.	12
	20b	Present results of all statistical syntheses conducted. If meta-analysis was done, present for each the summary estimate and its precision (e.g. confidence/credible interval) and measures of statistical heterogeneity. If comparing groups, describe the direction of the effect.	12
	20c	Present results of all investigations of possible causes of heterogeneity among study results.	SF
	20d	Present results of all sensitivity analyses conducted to assess the robustness of the synthesized results.	SF
Reporting biases	21	Present assessments of risk of bias due to missing results (arising from reporting biases) for each synthesis assessed.	SF
Certainty of evidence	22	Present assessments of certainty (or confidence) in the body of evidence for each outcome assessed.	SF
**DISCUSSION**			
Discussion	23a	Provide a general interpretation of the results in the context of other evidence.	16
	23b	Discuss any limitations of the evidence included in the review.	17
	23c	Discuss any limitations of the review processes used.	17
	23d	Discuss implications of the results for practice, policy, and future research.	17
**OTHER INFORMATION**			
Registration and protocol	24a	Provide registration information for the review, including register name and registration number, or state that the review was not registered.	17
	24b	Indicate where the review protocol can be accessed, or state that a protocol was not prepared.	
	24c	Describe and explain any amendments to information provided at registration or in the protocol.	
Support	25	Describe sources of financial or non-financial support for the review, and the role of the funders or sponsors in the review.	26
Competing interests	26	Declare any competing interests of review authors.	26
Availability of data, code and other materials	27	Report which of the following are publicly available and where they can be found: template data collection forms; data extracted from included studies; data used for all analyses; analytic code; any other materials used in the review.	

For peer review only

PRISMA 2020 Checklist

From: Page MJ, McKenzie JE, Bossuyt PM, Boutron I, Hoffmann TC, Mulrow CD, et al. The PRISMA 2020 statement: an updated guideline for reporting systematic reviews. BMJ 2021;372:n71. doi: 10.1136/bmj.n71

For more information, visit: http://www.prisma-statement.org/

For peer review only

BMJ Open

A Systematic Review of Educational Interventions to improve the Menstrual Health of young adolescent girls.

Journal:	*BMJ Open*
Manuscript ID	bmjopen-2021-057204.R2
Article Type:	Original research
Date Submitted by the Author:	03-May-2022

Complete List of Authors:	Evans, Rebecca; University of Warwick<br>Harris, Bronwyn; Warwick Medical School, University of Warwick, Coventry, Division of Health Sciences, ; University of the Witwatersrand, Centre for Health Policy, School of Public Health<br>Onuegbu, Chinwe ; University of Warwick, Health Sciences<br>Griffiths, Frances; University of Warwick, Warwick Medical School

**Primary Subject Heading**:	Public health
Secondary Subject Heading:	Sexual health
Keywords:	PUBLIC HEALTH, Health policy < HEALTH SERVICES ADMINISTRATION & MANAGEMENT, REPRODUCTIVE MEDICINE, PREVENTIVE MEDICINE

SCHOLARONE™
Manuscripts

I, the Submitting Author has the right to grant and does grant on behalf of all authors of the Work (as defined in the below author licence), an exclusive licence and/or a non-exclusive licence for contributions from authors who are: i) UK Crown employees; ii) where BMJ has agreed a CC-BY licence shall apply, and/or iii) in accordance with the terms applicable for US Federal Government officers or employees acting as part of their official duties; on a worldwide, perpetual, irrevocable, royalty-free basis to BMJ Publishing Group Ltd ("BMJ") its licensees and where the relevant Journal is co-owned by BMJ to the co-owners of the Journal, to publish the Work in this journal and any other BMJ products and to exploit all rights, as set out in our licence.

The Submitting Author accepts and understands that any supply made under these terms is made by BMJ to the Submitting Author unless you are acting as an employee on behalf of your employer or a postgraduate student of an affiliated institution which is paying any applicable article publishing charge ("APC") for Open Access articles. Where the Submitting Author wishes to make the Work available on an Open Access basis (and intends to pay the relevant APC), the terms of reuse of such Open Access shall be governed by a Creative Commons licence – details of these licences and which Creative Commons licence will apply to this Work are set out in our licence referred to above.

Other than as permitted in any relevant BMJ Author's Self Archiving Policies, I confirm this Work has not been accepted for publication elsewhere, is not being considered for publication elsewhere and does not duplicate material already published. I confirm all authors consent to publication of this Work and authorise the granting of this licence.

For peer review only

[Type here]

TITLE PAGE

Title:

A systematic Review of Educational Interventions to improve the Menstrual Health of young adolescent girls.

Authors:
Ms Rebecca Lane-Evans, Warwick Medical School, Warwick Medical School, University of Warwick, Coventry, CV4 7AL United Kingdom. Rebecca.R.Evans@warwick.ac.uk

Ms Chinwe Onuegbu, Warwick Medical School, Warwick Medical School, University of Warwick, Coventry, CV4 7AL United Kingdom. Chinwe.Onuegbu@warwick.ac.uk

Dr Bronwyn Harris, Warwick Medical School, Warwick Medical School, University of Warwick, Coventry, CV4 7AL United Kingdom. B.Harris.2@warwick.ac.uk

Professor Frances Griffiths, Warwick Medical School, Warwick Medical School, University of Warwick, Coventry, CV4 7AL United Kingdom. F.E.Griffiths@warwick.ac.uk

Corresponding author

Rebecca.R.Evans@warwick.ac.uk

Word Count: 4186

For peer review only - http://bmjopen.bmj.com/site/about/guidelines.xhtml

[Type here]

ABSTRACT

Objectives

To systematically review interventions that include an element of menstrual education delivered to young adolescent girls.

Design

This was a systematic review and meta-analysis. Selected articles were quality assessed using the Mixed Methods Assessment Tool (MMAT) quality appraisal checklist. A meta-analysis was conducted on a subset of articles, and the effect size of the intervention was calculated using Cohen's d. A logic model was constructed to frame the effect of Menstrual Education Interventions on Menstrual Health.

Setting

Papers reporting on interventions in High and Low-and Middle-Income countries were sought.

Information Sources

Seven electronic databases were searched for English-language entries that were published between January 2014 and May 2020.

Participants

The interventions were aimed at younger adolescent girls aged 10 -14 years old.

Interventions

The interventions were designed to improve the Menstrual Health of the recipients, by addressing one or more elements of Menstrual Knowledge, Attitude or Practices (KAP).

Eligibility criteria

Interventions which had not been evaluated were excluded.

Primary and Secondary Outcomes

The most common type of output was a difference in knowledge or skill score ascertained from a pre- and post-test. Some studies measured additional outcomes, such as attitude or confidence.

Results

Twenty-four eligible studies were identified. The number of participants varied from 1 to 2564. All studies reported improvements in menstrual Knowledge, Attitudes and Practices. The meta-analysis indicates that larger effect sizes were attained by those that encouraged discussion than those that distributed pamphlets.

Conclusions

[Type here]

For peer review only

[Type here]

Education interventions are effective in increasing the menstrual knowledge of young adolescent girls and skills training improves competency to manage menstruation more hygienically and comfortably. Interactive interventions are more motivating than didactic or written. Sharing concerns gives girls confidence and helps them to gain agency on the path to menstrual health.

STRENGTHS AND LIMITATIONS

- This review is of the international literature including high and low-and-middle-income countries
- It utilises mixed methods approaches and synthesises both quantitative and qualitative studies
- A logic model has been constructed to frame the effect of Menstrual Education Interventions on Menstrual Health
- There may be publication bias in the reporting of positive outcomes only
- A full meta-analysis was not possible due to heterogeneity in methods

For peer review only

INTRODUCTION

Globally, young adolescent girls are ill-prepared for menarche and menstruation (1–4). In many cultures, menstruation is a taboo subject (5) and many girls are ignorant of it until they start bleeding (6). Negative experiences of menarche and early menstruation can cause poor Menstrual Health (7,8).

'Menstrual Health' is an emerging area of Health Research. It is a broad term which encompasses the hygienic management of menstruation and the psychological components of well-being such as confidence, dignity and self-esteem (9,10). It is an expansion of the concept of Menstrual Hygiene Management (MHM), mostly used in Low- and Middle- Income Country (LMIC) contexts to describe the challenges of hygienically managing menstruation with a lack of resources, especially pads, water and soap (11–13). The use of dirty rags to absorb menstrual blood has been proposed as a cause of Reproductive Tract Infections (RTIs) and cervical cancer (14–16), as infectious agents may be introduced into the reproductive tract from such materials. Even clean materials that are not changed regularly may smell (3) and some materials can cause chafing and irritation. Without sufficient water to wash the blood from their genitals or their hands (17), girls can become uncomfortable and anxious (18). Several studies in LMIC have shown that menstruation is associated with a reduction in participation in activities and an increase in school absenteeism (15,19,20). In High Income Countries (HIC), 'Period Poverty' has only recently been recognised as an issue for certain groups, such as homeless women (21), but a whitepaper by PHS Group UK (a Hygiene Services provider in the UK) suggests period poverty is more wide-reaching, particularly amongst school girls, and is a factor contributing to menstrual anxiety and school absenteeism (22). Significantly less research has been conducted in HICs around menstruation and participation, although it is widely recognised that girls drop out of sports activities around the age of puberty (23,24).

[Type here]

[Type here]

Discussing menstruation is almost universally a taboo, making it difficult for girls to learn about it and know what is normal or when to seek help. A study by Gultie *et al* 2014 into Menstrual Knowledge of adolescents in Ethiopia found that 33% of participants never talked about it with anyone (25). In Jordan, a phenomenological analysis of experiences of menarche reported that girls believed talking about menstruation was 'rude' (26). A study into puberty communication in the Czech Republic and China found that both men and women were complicit in perpetuating menstrual stigma, with mothers instructing their daughters to keep their menstrual status 'secret' (27). In India, Rani *et al* 2016 found that although 61.3% of adolescent girls suffered from debilitating dysmenorrhea, they felt they were expected to 'tolerate' it as a natural process and only 1.6% had ever consulted a physician (14).

Menstrual knowledge usually comes from mothers (8) but a number of studies have shown that the knowledge of mothers themselves may be incomplete (28,29) and they may actually perpetuate cultural myths and misinformation (30,31). In HICs such as Australia and the UK, menstruation may be taught at school as part of sex education, if it is mentioned at all, but many girls do not get the practical information that they need (32,33). In both HIC and LMIC non-government agencies have stepped in to try to plug the gap by providing Menstrual Education Interventions.

In this review our objective was to describe and evaluate the impact of menstrual education interventions intended to equip young adolescent girls with the knowledge and skills to promote Menstrual Health.

Two previous reviews of papers published prior to January 2015 focussed on the more narrow 'Menstrual Hygiene Management' in LMIC (34,35). The term Menstrual Health is now preferred to hygiene, partly to avoid the suggestion that menstruation *per se* is unclean, but mostly to emphasize the holistic nature of the menstrual experience. This literature review only includes publications since 2014 in order to capture that change.

For peer review only

METHODS

This is a systematic review of published literature of interventions that include an element of menstrual education delivered to young adolescent girls. We report according to the Preferred Reporting Items for Systematic Reviews and Meta-Analyses (PRISMA) guidelines.

Patient and Public Involvement

No patient involvement.

Publication Date and Language

This review draws upon papers published from January 2014 until May 2020 to bring the field up to date. Only reviews published in the English language have been included.

Participants

For this review we were interested in interventions targeted at young adolescent girls aged 10 -14 years old.

[Type here]

Inclusion and Exclusion Criteria

Studies of interventions which had been evaluated were sought.

All interventions which had a component of menstrual information transfer were included if the intervention sought to:

- Increase knowledge of menstruation to reduce anxiety and shame, and normalise the experience
- Increase skills and competencies to manage menstruation comfortably and hygienically
- Increase awareness of strategies for self-care of menstrual symptoms

Interventions that were straightforward 'Menstrual Education' in which lessons about puberty, anatomy, and hygiene were delivered by teachers or other educators, to both boys and girls, were eligible. Interventions focussed on skills training, such as correct menstrual cup insertions, and delivered by nurses or other key workers were also eligible. Programmes that facilitated peer or self-guided learning through the provision of resources or spaces (both physical and remote) for learning to occur were also eligible for inclusion.

Studies were excluded if the improvements were in hardware such as toilets or pads without any accompanying education or training, or if they provided hygiene education without reference to menstruation specifically. Studies were excluded if they were about abnormal menstruation, menstrual problems as a co-morbidity, or if the research was investigating endocrinology or non-human models. Studies that described existing Knowledge, Attitudes and Practice without any intervention were also excluded.

Studies that included adolescents up to the age of 19 were not excluded if the aim were to instruct menstruators with limited experience of menstruation. Some studies included older girls because they were intellectually disabled and were part of the intervention based on intellectual age rather than chronological age. Some studies included older girls because they were members of classes assigned by grade rather than age. Studies about interventions aimed at adult women were excluded.

Study design and quality were not part of the criteria to capture as broad an interpretation of menstrual education as possible.

Protocol for Identification of Academic Literature

Screening

As the field of Menstrual Health Education is highly interdisciplinary, we searched key medical and social science databases: ASSIA Applied Social Science Index and Abstracts; CINAHL Cumulative Index to Nursing and Allied Health Literature; EMBASE Excerpta Medica database; MEDLINE Medical Literature Analysis and Retrieval System Online; Sociological Abstracts; Web of Science; IBBS International Bibliography of the Social Sciences; TRoPHI Trials Register of Promoting Health Interventions.

The search parameters combined the target population, menstruation, education and programme (table 1).

For peer review only - http://bmjopen.bmj.com/site/about/guidelines.xhtml

[Type here]

Table 1. Search Terms Used

**Search Terms Used**
Search term 1<br>'adolescen* OR girl* OR teenage* OR youth* OR young OR pre-adolescen* OR school-girl OR Out-of-School-Youth OR OOSY OR female OR woman<br>AND Search term 2<br>Menstrua* OR menarche* OR mense* OR catamenia OR menarche* OR menstrual health OR menstrual hygiene OR menstrual management OR sanitation OR menstrual etiquette<br>AND Search term 3<br>know* OR understand*OR manage* OR learn* OR apprehen* OR comprehensi* OR educat* OR aware* OR familiar* OR proficien*<br>AND Search term 4<br>AND arrangement* OR evaluat* OR initiative* OR intervention*OR model* OR package* OR pilot* OR program* OR project* OR provision* OR regime* OR scheme* OR strateg* OR trial* OR approach* OR polic*

**Database Returns**		
**Database**	**Search Terms and filters**	**Returns**
ASSIA (proquest)	1 (ab), 2 (ti), 3 (ab), 4 (ab)<br>01/01/14 to 01/09/19	15
CINAHL	1 (ab), 2 (ti), 3 (ab), 4 (ab)<br>01/01/14 to 01/09/19	126
EMBASE<br>Including MEDLINE	1 (ab), 2 (ti), 3 (ab), 4 (ab)<br>2014 -2019<br>English, Female	732
Sociological abstracts (proquest)	1 (ab), 2 (ti), 3 (ab), 4 (ab)<br>01/01/14 to 01/09/19	14
Web of Science	TS=(1), (3), (4) AND Ti=(2)<br>Last 5 years	323
IBBS (proquest)	1 (ab), 2 (ti), 3 (ab), 4 (ab)<br>01/01/14 to 01/09/19	26
TRoPHI (Eppi centre, google)	No wild cards<br>Title<br>Menstruation and Knowledge<br>2014-2019	4
**TOTAL SAVED to EXCEL**		**1240**

Two reviewers (RE and CO) screened abstracts and full texts of all citations obtained for eligibility independently. Data extraction of eligible material and the quality assessment was conducted by RE using a data extraction framework agreed upon by FG, BH and RE. The quality assessment tool was agreed upon by RE, FG and BH. RE used the MMAT to assess the quality and this was verified by FG and BH.

Outputs and Data Extraction

The outputs of Menstrual Education are changes to Menstrual Knowledge, Attitudes and Practices (KAP). In quantitative studies, Pre- and Post-Intervention KAP scores were collected, as well as data to support the validity of the scores, such as sample frame and number of participants. Other descriptive measures used in qualitative studies and contextual factors that might have a bearing on the outputs were also recorded where given.

[Type here]

For peer review only - http://bmjopen.bmj.com/site/about/guidelines.xhtml

[Type here]

Quality Assessment

We used the Mixed Methods Appraisal Tool (MMAT) for quality assessment because it is suitable for a variety of study designs. The MMAT components focus on the clarity of the research questions and the appropriateness of the data collection methods. Our intention was to consider the MMAT assessment in the interpretation of study findings.

Analysis and Synthesis

Quantitative analysis

The results of quantitative studies were tabulated for comparison. Not all data could be converted but for studies that reported quantitative results with a pre- and post- test score of Menstrual Knowledge, we found the effect size by calculating the Standard Mean Difference using Cohen's d. Effect sizes can lie between 0 and infinity. Cohen suggested that a small effect size is a value of 0.2, a medium one is 0.5 and a large one is 0.8. Some social science disciplines report much larger sizes and the statistical guidance was revised to suggest that a medium effect is the average of those in the relevant literature (36,37). However, Cohen's d has not previously been calculated for this discipline. We found the average of those in this review and ranked the results accordingly(38).

Qualitative analysis

A Qualitative Comparative Analysis (QCA) (39) was made of all studies by scrutinising the data extracted for common themes and reporting them in a narrative summary.

Synthesis: Building A Logic Model

Logic models enable the key findings of the analysis to be synthesized into a theory of change. We constructed a logic model to frame the effect of Menstrual Education Interventions on Menstrual Health.

RESULTS

A total of 1240 papers were recovered using the search terms. 900 remained after the removal of duplicates and 48 were saved to Excel for full text screening. 852 did not meet the inclusion criteria. This was largely because they were not interventions but studies of menstruation (Figure 1).

PRIFLO

Figure 1. PRISMA Flow-chart

Proximal, intermediate, distal and macro-distal outcomes and indicators of a menstrual health intervention. Menstrual Education and Training Inputs act on Girls' Menstrual Knowledge, Attitude and Practices, and Outputs are measured by increasing KAP score. Positive feedback operates as girls gain knowledge and skills they become more confident to seek further knowledge and skills.

Study Characteristics

Twenty-four papers met the inclusion criteria: They were grouped into the following categories of study design according to the MMAT Quality Appraisal tool: 12 Randomised Controlled Trials (RCTs); 5 quantitative descriptive studies; 1 quantitative non-random study; 5 mixed-methods studies and 1 qualitative study. *See Supplementary Files for further detail on study design.*

[Type here]

For peer review only - http://bmjopen.bmj.com/site/about/guidelines.xhtml

[Type here]

The study dates ranged from 2012 to 2017, with dates of publication ranging from 2014 to 2019. Studies were undertaken in Iran (six), Turkey, Indonesia (two), Ethiopia, India (four), Bangladesh, Uganda (four), USA, Nepal, Kenya (two) and China.

Of the studies, two included boys (10,20,40); one included mothers (29,31) and two focussed on intellectually-disabled adolescent girls (41,42). The number of study participants varied from 1 to 2564. The total number of participants in the 20 different intervention studies was 10362.

The amount of time after the completion of the intervention to the assessment varied from the same day to up to 5 years, with 13 of the studies in the range of 1 – 9 months, and the mode being 1 month. A common theme was allowing one menstrual cycle to pass before re-testing.

Delivery

Eight of the interventions used health professionals as instructors (6,9,10,31,41,43–46). Five were researcher-led (31,42,47–49); five were led by teachers (20,50–53); two used peer-educators (9,44) and the others did not make it clear. A wide range of intensity and duration models were used, ranging from three 5-minute videos in the space of one afternoon (40) to 1 hour per day for four months (43). Only two embedded menstrual education into the school curriculum (52,53).

Quality Assessment

The methodological quality of study designs was mixed: Eleven were rated as high quality and thirteen as moderate to low. Those considered to be of the highest quality were randomized controlled trials which included comparison groups. Some of the studies (nine) did this at the whole-school level which is recommended in educational interventions to prevent contamination of the intervention group with the control (54). The research questions were clear and the data collection methods appropriate. Of the other studies, several methodological limitations were noted; commonly, neither the delivery team nor the participants were blinded (nine); adequate randomization of the participants was lacking (ten) and /or relevant confounds were not identified or controlled (four). The quality of data analysis also varied considerably, with the weakest having small sample sizes and no measure of statistical significance (two).

Interventions

There was a range of intervention types. Eight of the interventions employed traditional education in the form of didactic teaching, sometimes supplemented with posters, flip-charts, and question-and-answer sessions (19,43,45,48,49,52,53,55). Two interventions employed a more formal lecture presentation and gave out some supporting literature(31,47). Two interventions used stories and video presentations (40,42) and two interventions distributed puberty books (4,50) without further teacher input. Three interventions facilitated learning through peer-education (9,44,56) and seven different interventions focussed on Menstrual Hygiene Management training: some demonstrated pad usage with a menstrual kit (10,20,42,51) or using a doll (41) and two instructed participants on the use of menstrual cups (46,57).

Aims of the Studies

The common aim of the studies was to evaluate the impact of a Menstrual Education Intervention on Menstrual Health for adolescent girls. A number of studies were more broadly about puberty

[Type here]

For peer review only - http://bmjopen.bmj.com/site/about/guidelines.xhtml

education (31,40,47,48,50,52) Several studies measured Menstrual Knowledge, Attitudes and Practices (KAP) (4,6,56,31,40,43,44,49–51,55). Nearly all of the studies used a pre-test, post-test model but one study used a post-test only model (40). A small number specifically focussed on Menstrual Hygiene Management and evaluated training on pad replacement or cup insertion (10,20,35,41,42,44,45,57,58)

Analysis

The quantitative and qualitative results are reported under four main themes: Menstrual Knowledge, Menstrual Attitudes, Menstrual Practices and Multi-component interventions.

Quantitative results

Menstrual Knowledge.

A meta-analysis was conducted on 11 studies which measured a change in menstrual knowledge following an intervention. A visual inspection of forest plots showed that all studies found a significant improvement in menstrual knowledge. Where studies reported the mean and standard deviation of a menstrual knowledge questionaire, we calculated the effect size using Cohen's d (table 2).

The average effect size of studies in this review was 3.44. Taking this as a medium effect size, we ranked them lowest – highest and suggest that <2 is low and >5 is high. Where we could not calculate an effect size, we have calculated % change in score. Due to the limited and heterogeneous nature of the data, we interpret the results only relative to the other studies in this review.

The effect size of those that distributed pamphlets and books was lowest at 0.33 (31), followed by those that showed videos 1.40 (40) and then lectures with question and answer sessions 2.13 (49) and 4.81 (55). Small group or peer-teaching was high at 5.337 and 10.044 respectively. Large effect sizes may occur due to small sample sizes.

For peer review only

For peer review only - http://bmjopen.bmj.com/site/about/guidelines.xhtml

Table 2 Effect size of interventions designed to improve menstrual knowledge

Study type and measure	First author and date of publication	Intervention	Number of questions / N	Reliability/ Cronbach's alpha (except where stated otherwise)	Sample size / n	Treatment	Mean / arbitrary units (except where stated otherwise)	Standard Deviation / Arbitrary units (except where stated otherwise)	Number of individuals scoring > 75% (good)	% change in number of individuals scoring >75%	Statistical test / p value	Effect size / Cohen's d /Rank and Impact
**Control and intervention pre-test, post test**	Blake et al 2018	Distribution of puberty education books in the local language	9	0.77	318	control	(Mean Difference = 0.18)	(Pooled SD = 1.4)	No data		Wald Chi-squared , <0.001	**0.79 Second Low**
					318	intervention	(MD = 1.06)	(Pooled SD = 1.52)				
	Jarrahi et al 2020	Small group and peer teaching	34	0.78	30	control pre	45.1	8.4	No data		Kruskal-Wallis test, <0.001	**small group 5.34 Eighth High**
					30	control post	52.2	1				
					30	intervention 1 pre (small group)	48.3	6.1				
					30	intervention 1 post	84.5	8.5				**Peer group 10.04 Nineth High**
					30	intervention 2 pre (peer)	44.1	1.7				
					30	intervention 2 post	93.3	5.7				

For peer review only - http://bmjopen.bmj.com/site/about/guidelines.xhtml

	Setyowati et al 2019	Booklet distriution	14	0.886	87	control pre	No data	No data	No data	No data	Chi-squared <0.001	(medium)
					87	control post			17.2			
					87	intervention pre			58.6	54.95		
					87	intervention post			90.8			
	Sharma et al 2015	Interactive Teaching programme led by School Nurses	15	test, retest r = 0.93	25	control pre	8.02	No data	No data		t-test <0.001	**4.48 Sixth Medium**
					25	control post	8.06					
					25	intervention pre	8.04					
					25	intervention post	12.6					
	Su et al 2016	Lecture, question and answer session	13	KR20= 0.64	56	control pre	5.5	2.54	No data		t-test <0.001	**2.13 Fifth Medium**
					56	control post	5.71	2.3				
					60	intervention pre	5.73	2.56				
					60	intervention post	10.22	1.92				
	Valizadeh et al 2017	Lecture, booklet and pamphlets	15	0.72	120	control pre	8.5	2.5	No data		general linear model <0.002	**0.33 First Low**
					120	control post	9.1	2.4				
					124	intervention pre	8.2	2				

For peer review only - http://bmjopen.bmj.com/site/about/guidelines.xhtml

					124	intervention post	9.8	1.8				
	Kheirollahi et al 2017	Lecture, question and answer session	23 (100 point scale)	0.8	76	control pre	52.58	6.58	No data		t-test <0.001	**4.81 Seventh Medium**
					76	control post	52.77	6.87				
					76	intervention pre	55.83	6.77				
					76	intervention post	86.36	7.11				
**Control and intervention post-test only**	Hurwitz et 2017	Health education videos, using animation	27	0.72	40	control	11.27	3.73	No data		t-test <0.001	**1.40 Third Low**
					40	intervention	15.67	2.4				
**one group pre-test, post test**	Arasteh et al 2019	Group counselling and pamphlets	15	0.8	30	pre	6.8	3.32	No data		Chi-squared <0.001	**1.64 Fourth Low**
					30	post	11.3	2				
	Haque et al 2014	Training with Field manual by medical professionals	10	0.73	416	pre	No data	No data	51	61.57	chi-squared <0.05	(high)
					416	post			82.4			
	Chadawada et al 2017	Didactic teaching with posters, and videos	4	no data	250	pre			72.7	19.12	chi-squared <0.05	(low)
					250	post			86.6			

For peer review only

Menstrual Attitudes. Five studies measured Menstrual Attitudes (4,40,47,49,51).

Four interventions reported significantly different ($p<0.05$) attitude scores, and three of those provided pamphlets that addressed cultural restrictions(4,47,51). The other was an intervention on dysmenorrhea and self-care and included pamphlets with video and peer-sharing. Girls who had taken part had a significant increase in confidence and decrease ($p<0.001$) in 'bothersome' menstrual attitude (49). The only intervention which did not find a significant difference in attitude pre- and post-test involved puberty education videos shown to early adolescent boys and girls (40).

Menstrual Practice. An intervention that trained intellectually-disabled adolescents in an 18-item pad replacement skill set found that pre-training and post-training differences were statistically significant ($p<0.001$) (41). A feasibility trial into the use of the menstrual cup by school girls in Kenya (46) found that usage increased as time went on and culminated in 96% usage after nine months. There was also an increase in hygiene, with the menstrual cup reported as reducing the prevalence of STIs from 19.2% to 12.9% ($p=0.018$) (57).

Multi-component interventions

An education intervention in India was part of a bigger project that involved pad provision and improved sanitation in schools. After four years, compared to unimproved schools, school attrition had fallen from 11% to 6% ($P<0.003$) (53). The effect of menstrual education alone cannot be separated out.

Qualitative results

Menstrual Knowledge. All studies reported an increase in Menstrual Knowledge. Interventions that used peer education and group counselling (9,44) were as effective as those delivered by medical professionals (51).

Those interventions that had a degree of interactivity were more effective than those that only gave out information. Those that encouraged discussions found that they led to an increased willingness to talk about menstruation and a greater awareness of what is normal (9,48).

Only two interventions embedded menstrual education into the school curriculum (52,53). In Nepal, some schools had received the WASH in Schools programme (WinS) (59). However, the girls were highly critical of their teachers, especially male teachers. They complained that 'Teachers often got embarrassed, referred students to their textbook, and did not answer questions' (52).

Menstrual Attitudes. Most studies commented on an improved menstrual attitude and one noted a reduction in anxiety (48). More than one study noted an improvement in confidence in performing menstrual health care behaviour, such as requesting pain relief for dysmenorrhea (49). Some studies observed an increased confidence of girls to push back against cultural restrictions, or harmful practices (9,19,48,51,60).

Menstrual Practices. Skills are required to use pads and cups so that they are positioned correctly, are comfortable and don't leak. The cup feasibility trial in Kenya found that on-going training and support may be required to master the technique over a period of six months to one year (46,57). Education was also found to be an important component of skill acquisition in Uganda, where pad-provision accompanied by education was shown to be more effective than pad-provision alone (19).

Multi-component interventions

For peer review only - http://bmjopen.bmj.com/site/about/guidelines.xhtml

[Type here]

The MENISCUS intervention in Uganda attributed its success to the synergy of five combined elements; teacher training on puberty education, a drama skit, pad provision, pain relief provision, and Water, Sanitation and Hygiene (WASH) facility improvements (10).

Synthesis: The Logic Model

Guided by logic models developed for school-based interventions (61), we worked backwards from the higher order aim of good Menstrual Health to propose a chain of causal events.

The aim of good Menstrual Health is the distal outcome to the intervention. It is characterized by girls feeling empowered and having agency to make choices about their own bodies and lives. They can choose a suitable menstrual product to meet their individual needs. They track their menstrual cycle to be well-prepared so that they are not caught out at school and have to go home, and they engage as necessary with reproductive health services, without shame or stigma. Girls that have agency are able to control their menstruation and not the other way around. They can focus on their school work and reach their potential.

Preceding the distal outcome is the intermediate outcome; unrestricted mobility and participation. Girls should be able to carry out normal activities such as eating/drinking with the family, attending school and playing sport when they are menstruating. This requires confidence in their own ability to manage outside of the confines of the house, and determination to enter spaces from which they are traditionally excluded.

Below that is the proximal outcome; hygienic and comfortable menstruation management. Girls should be able to use suitable menstrual products. They should be able to use water and soap to clean away menstrual blood, and they should be able to practise self-care to relieve the symptoms of dysmenorrhea, such as yoga. If they need painkillers or a rest, they should be able to request them of parents and teachers without embarrassment.

Menstrual KAP underpins these outcomes. In a theory of change, girls require knowledge of the menstrual cycle to prepare products. They may need skills to use the products correctly to avoid the risk of discomfort, leaks or of contracting reproductive tract infections. They may need confidence to ask for products and services. They may need awareness of self-care practices. They should know what is normal and when to seek help.

Inputs and Outputs to the Logic Model

Menstrual Education and training are the inputs. The output is improved menstrual knowledge, attitude and practices (KAP). The outputs are linked to the outcomes.

LOGMOD

Figure 2 The Logic Model

The results of the review provide evidence that menstrual education improves the menstrual knowledge, attitude and practices (KAP) of young adolescent girls. It is suggested that increasing the menstrual KAP of girls increases their confidence to seek further knowledge and skills in a positive feedback loop (see fig 2). Menstrual Education is viewed as underpinning the logic model and is the first step to achieving Menstrual Health.

[Type here]

For peer review only - http://bmjopen.bmj.com/site/about/guidelines.xhtml

[Type here]

DISCUSSION

All twenty-four included studies that evaluated some form of menstrual education intervention reported that there was a measurable improvement in the Menstrual Knowledge, Attitude or Practices of young adolescent girls. Menstrual experience and need may be the motivators, as there was little improvement in the knowledge or attitude of very early adolescent girls or boys.

Most of the interventions were delivered in the school setting where it is relatively easy to reach the target group, although only two embedded the education into the school curriculum. In theory, schools should have good coverage and objectivity for delivery of this sensitive information at an appropriate time. However, teachers themselves may be ill-equipped to teach about menstruation without proper training (62).

A larger effect was gained with the more interactive interventions that included question and answer sessions. We suggest that this relates to the higher degree of participation, and concurs with current educational philosophy about the importance of active learning (63), based on constructivist theory (64,65). Gardner added to Dewey's early work on active learning when he described 'transformative' teaching. This involves using a range of methods that encourage the learner to find their own entry point and engage with the subject, often utilising space and creativity, and linking with their own experience(66). Discussing menstruation gives the girls agency to determine what it is that they need to know for themselves.

Those interventions that demonstrated skills and allowed for physical touch were also very effective. Other hygiene interventions that have been evaluated have pointed to the positive impact of a physical interaction with the tools of behaviour change (67).

A logic model has been constructed to frame the effect of menstrual education interventions on Menstrual Health. Menstrual Education is seen as underpinning all desirable outcomes and programmes can be optimised by including an education aspect. There was evidence that interventions were successful in normalising menstruation and improving menstruation management, which is the proximal outcome of the logic model. There was more limited evidence for an improvement in school attendance and mobility or a refusal to accept menstrual restrictions. These are intermediate outcomes and it might be expected to take some time to move from the proximal to the intermediate outcomes in the theory of change.

Multi-component interventions may be more successful than single components in achieving the distal outcome of Menstrual Health and Well-being. Girls need an enabling environment as well as knowledge. From a constructivist perspective which places learning within a social context(65,68) interventions that seek to improve the menstrual literacy of the whole community and reduce menstrual stigma may be more effective in achieving the macro-distal outcomes of girl empowerment and gender equality.

Implications for Policy and Practice

This review provides evidence that menstrual education has a positive effect on the menstrual knowledge, attitudes and practices of adolescent girls and needs to be delivered by trained personnel who are confident to lead discussion. Especially but not exclusively in LMIC, where resources are limited, it would be prudent to ensure that menstrual education is embedded into the school curriculum and that teachers receive specialist training.

[Type here]

For peer review only - http://bmjopen.bmj.com/site/about/guidelines.xhtml

[Type here]

Progress towards Menstrual Health is limited without an enabling environment. In order to achieve the more distal outcomes of the logic model, programme and policy makers need to address the menstrual literacy of the wider population. Multi-component interventions that speak to different actors and include hardware and software provision alongside menstrual education may make Menstrual Health more attainable.

Covid-19

This review was carried out on studies conducted before the Global Pandemic began in March 2020. The subsequent lockdown has had a profound effect on education, and many programmes have had to go on-line. We would encourage menstrual educators to be mindful of the benefits of interaction and make use of on-line teaching platforms that facilitate discussion in break-out rooms.

Limitations of this Review

The review was carried out in the English language, which may have missed some publications. Because menstrual health is an emerging topic with evolving terminology, search terms may not have adequately captured all currently used descriptors.

As a mixed methods review, there are a number of systemic limitations derived from comparing heterogeneous data sets. The studies did not measure the same outputs and the methodological quality of the studies was mixed. Two were aimed at the intellectually-disabled. It is possible that the level of menstrual knowledge was so low at base-line that any educational intervention would result in an improvement.

Although all interventions reported positive outcomes, this may be due to publication bias, where only significant results are shared. Cohen's d has not previously been calculated for this discipline and therefore the magnitude of the effect size can only be considered relative to others in this review.

The number of studies was small, and only one study was from a High Income Country, so it is difficult to say how applicable the conclusions are to a HIC. More research needs to be done in this area, particularly as period poverty has been increasingly reported since the start of the pandemic in HIC.

For peer review only

REGISTRATION

For this review, a protocol was not prepared or registered.

REFERENCES

[Type here]

[Type here]

1. Chandra-Mouli V, Patel S V. Mapping the knowledge and understanding of menarche, menstrual hygiene and menstrual health among adolescent girls in low- and middle-income countries. Reprod Health. 2017;14.

2. Marván ML, Alcalá-Herrera V. Age at Menarche, Reactions to Menarche and Attitudes towards Menstruation among Mexican Adolescent Girls. J Pediatr Adolesc Gynecol. 2014;27(2):61–6.

3. Mason L, Nyothach E, Alexander K, Odhiambo FO, Eleveld A, Vulule J, et al. "We keep it secret so no one should know" - A qualitative study to explore young schoolgirls attitudes and experiences with menstruation in rural Western Kenya. PLoS One. 2013;8(11).

4. Setyowati S, Rizkia M, Ungsianik T. Improving Female Adolescents' Knowledge, Emotional Response, and Attitude toward Menarche following Implementation of Menarcheal Preparation Reproductive Health Education. Asian/Pacific Isl Nurs J. 2019;4(2):84–91.

5. Shah V, Nabwera HM, Sosseh F, Jallow Y, Comma E, Keita O. A rite of passage : a mixed methodology study about knowledge , perceptions and practices of menstrual hygiene management in rural Gambia. BMC Public Health. 2019;19(277):1–15.

6. Sharma R, Sandhya N, Deepika K, Varsha S, Vardha. Menstrual hygiene among adolescent girls. Indian J Community Heal. 2015;27(3):376–80.

7. Marván ML, Alcalá-Herrera V. Age at menarche, reactions to menarche and attitudes towards menstruation among Mexican adolescent girls. J Pediatr Adolesc Gynecol. 2014;27(2):61–6.

8. Coast E, Lattof SR, Strong J. Puberty and menstruation knowledge among young adolescents in low- and middle-income countries: a scoping review. Vol. 64, International Journal of Public Health. Springer International Publishing; 2019. p. 293–304.

9. Ramaiya A, Malhotra A, Cronin C, Stevens S, Kostizak K, Sharma A, et al. How does a Social and Behavioral Change Communication Intervention Predict Menstrual Health and Hygiene Management: A Cross-Sectional Study. BMC Public Health. 2019;19(1):1–12.

10. Nalugya R, Tanton C, Hytti L, Kansiime C, Nakuya K, Namirembe P, et al. Assessing the effectiveness of a comprehensive menstrual health intervention program in Ugandan schools (MENISCUS): process evaluation of a pilot intervention study. Pilot Feasibility Stud. 2020;6(1):1–15.

11. House S, Mahon T, Cavill S. Menstrual Hygiene Matters: a resource for improving menstrual hygiene around the world. Reprod Health Matters. 2013 Jan;21(41):257–9.

12. Hennegan JM. Menstrual Hygiene Management and Human Rights: The Case for an Evidence-Based Approach. Women's Reprod Heal. 2017;

13. Ellis A, Haver J, Villasenor J, Parawan A, Venkatesh M, Freeman MC, et al. WASH challenges to girls' menstrual hygiene management in Metro Manila, Masbate, and South Central Mindanao, Philippines. Waterlines. 2016;

14. Rani A, Sharma MK, Singh A. Practices and perceptions of adolescent girls regarding the impact of dysmenorrhea on their routine life: A comparative study in the urban, rural, and slum areas of Chandigarh. Int J Adolesc Med Health. 2016;28(1):3–9.

15. Sumpter C, Torondel B. A Systematic Review of the Health and Social Effects of Menstrual Hygiene Management. PLoS One. 2013;8(4).

16. Tiwari A, Ekka IJ, Thakur R. Assessment of knowledge and practices regarding menstrual hygiene among adolescent girls of Government higher secondary school, station Murhipar,

[Type here]

For peer review only - http://bmjopen.bmj.com/site/about/guidelines.xhtml

[Type here]

Rajnandgaon (C.G.). Int J Community Med Public Heal. 2018;5(4):1335–8.

17. Sommer M, Kjellén M, Pensulo C. Girls' and women's unmet needs for menstrual hygiene management (MHM): The interactions between MHM and sanitation systems in low-income countries. Vol. 3, Journal of Water Sanitation and Hygiene for Development. 2013. p. 283–97.

18. Hennegan J. Inserting informed choice into global menstrual product use and provision. Lancet Public Heal. 2019;4(8):e361–2.

19. Hennegan J, Dolan C, Steinfield L, Montgomery P. A qualitative understanding of the effects of reusable sanitary pads and puberty education: implications for future research and practice. Reprod Health. 2017;14.

20. Kansiime C, Hytti L, Nalugya R, Nakuya K, Namirembe P, Nakalema S, et al. Menstrual health intervention and school attendance in Uganda (MENISCUS-2): A pilot intervention study. BMJ Open. 2020;10(2):1–11.

21. Sebert Kuhlmann A, Peters Bergquist E, Danjoint D, Wall LL. Unmet Menstrual Hygiene Needs Among Low-Income Women. Obstet Gynecol. 2019;133(2):238–44.

22. PHS. Lifting the lid on period poverty. 2019.

23. Whitehead S, Biddle S. Adolescent girls' perceptions of physical activity: A focus group study. Eur Phys Educ Rev. 2008;14(2):243–62.

24. Biddle SJH, Whitehead SH, O'Donovan TM, Nevill ME. Correlates of participation in physical activity for adolescent girls: A systematic review of recent literature. J Phys Act Heal. 2005;2(4):423–34.

25. Gultie T, Hailu D, Workineh Y. Age of Menarche and Knowledge about Menstrual Hygiene Management among Adolescent School Girls in Amhara Province, Ethiopia: Implication to Health Care Workers & School Teachers. PLoS One. 2014;9(9).

26. Al Omari O, Razeq NMA, Fooladi MM. Experience of Menarche Among Jordanian Adolescent Girls: An Interpretive Phenomenological Analysis. J Pediatr Adolesc Gynecol. 2016;29(3):246–51.

27. Raskova M, Stolinska DP. COMMUNICATION ABOUT PUBERTY AMONG MIDDLE-SCHOOL-AGED CHILDREN. In: Bekirogullari Z, Minas MY, Thambusamy RX, editors. International Conference on Education and Educational Psychology. 2017. p. 194–202.

28. Bennett C, Harden J. An exploration of mothers' attitudes towards their daughters' menarche. Sex Educ Soc Learn. 2014;14(4):457–70.

29. Afsari A. Predictors of knowledge of puberty health among girl students. Biosci Biotechnol Res Commun. 2017;10(2):264–9.

30. Sooki Z, Shariati M, Chaman R, Khosravi A, Effatpanah M, Keramat A. The Role of Mother in Informing Girls About Puberty: A Meta-Analysis Study. Nurs Midwifery Stud. 2016;Inpress(Inpress):1–10.

31. Valizadeh S, Assdollahi M, Mirghafourvand M, Afsari A. Educating Mothers and Girls about Knowledge and Practices toward Puberty Hygiene in Tabriz, Iran: A Randomized Controlled Clinical Trial. Iran Red Crescent Med J. 2017;19(2).

32. Tingle, C & Vora S. Break the Barriers : Girls ' Experiences of. Plan Int UK. 2018;1–68.

33. Collier-Harris CA, Goldman JDG. Is puberty education evident in Australia's first national

[Type here]

For peer review only - http://bmjopen.bmj.com/site/about/guidelines.xhtml

[Type here]

curriculum? Sex Educ Soc Learn. 2017;17(1):57–72.

34. Sumpter C, Torondel B. A Systematic Review of the Health and Social Effects of Menstrual Hygiene Management. PLoS One. 2013;8(4).

35. Hennegan J, Montgomery P. Do menstrual hygiene management interventions improve education and psychosocial outcomes for women and girls in low and middle income countries? A systematic review. PLoS One. 2016;11(2):1–21.

36. Gignac GE, Szodorai ET. Effect size guidelines for individual differences researchers. Pers Individ Dif. 2016;102(December):74–8.

37. Hemphill JF. Interpreting the Magnitudes of Correlation Coefficients. Am Psychol. 2003;58(1):78–9.

38. Bakker A, Cai J, English L, Kaiser G, Mesa V, Van Dooren W. Beyond small, medium, or large: points of consideration when interpreting effect sizes. Educ Stud Math. 2019;102(1):1–8.

39. Thomas J, Harden A. Methods for the thematic synthesis of qualitative research in systematic reviews. BMC Med Res Methodol. 2008;8(45):1–10.

40. Hurwitz LB, Lovato SB, Lauricella AR, Woodruff TK, Patrick E, Wartella E. "A New You, That's Who": an evaluation of short videos on puberty and human reproduction. Palgrave Commun. 2018;4(1).

41. Altundağ S, Çalbayram NÇ. Teaching menstrual care skills to intellectually disabled female students. Mol Ecol. 2016;25(13–14):1962–8.

42. Ariyanti TD, Royanto LRM. The Effectiveness of Social Stories and Video Modeling in Improving Self-Care Skills in Female Adolescents with Mild Intellectual Disabilities during Menstrual Periods. 2018;135(Iciap 2017):189–99.

43. Rani Chadalawada U, Devi S A, Rani M S. Effect of Health Education on Adolescent Girls Regarding Knowledge About Menstruation. J Evol Med Dent Sci. 2017;6(13):1040–3.

44. Jarrahi R, Golmakani N, Mazlom SR. Effect of menstrual hygiene education based on peer and small group teaching methods on hygiene behaviors in female adolescents: A comparative study. Evid Based Care J. 2020;10(1):70–4.

45. Montgomery P, Hennegan J, Dolan C, Wu M, Steinfield L, Scott L. Menstruation and the Cycle of Poverty: A Cluster Quasi-Randomised Control Trial of Sanitary Pad and Puberty Education Provision in Uganda. PLoS One. 2016;11(12).

46. Van Eijk AM, Laserson KF, Nyothach E, Oruko K, Omoto J, Mason L, et al. Use of menstrual cups among school girls: Longitudinal observations nested in a randomised controlled feasibility study in rural western Kenya. Reprod Health. 2018;15(1):1–11.

47. Afsari A, Mirghafourvand M, Valizadeh S, Abbasnezhadeh M, Galshi M, Fatahi S. The effects of educating mothers and girls on the girls' attitudes toward puberty health: A randomized controlled trial. Int J Adolesc Med Health. 2015;2015.

48. Mokari H, Khaleghparast S, Samani LN. Impact of Puberty Health Education on Anxiety of Adolescents. Int J Med Res Heal Sci. 2016;5(5, S, SI):284–91.

49. Su JJ, Lindell D. Promoting the menstrual health of adolescent girls in China. Nurs Heal Sci. 2016;18(4):481–7.

50. Blake S, Boone M, Yenew Kassa A, Sommer M. Teaching Girls About Puberty and Menstrual

[Type here]

For peer review only - http://bmjopen.bmj.com/site/about/guidelines.xhtml

[Type here]

Hygiene Management in Rural Ethiopia: Findings From a Pilot Evaluation. J Adolesc Res. 2018;33(5):623–46.

51. Haque SE, Rahman M, Itsuko K, Mutahara M, Sakisaka K. The effect of a school-based educational intervention on menstrual health: An intervention study among adolescent girls in Bangladesh. BMJ Open. 2014;4(7):1–9.

52. Morrison J, Basnet M, Bhatt A, Khimbanjar S, Chaulagain S, Sah N, et al. Girls' menstrual management in five districts of Nepal: Implications for policy and practice. Stud Soc Justice. 2018;12(2):251–72.

53. Sivakami M, van Eijk AM, Thakur H, Kakade N, Patil C, Shinde S, et al. Effect of menstruation on girls and their schooling, and facilitators of menstrual hygiene management in schools: Surveys in government schools in three states in India, 2015. J Glob Health. 2019;9(1).

54. Hutchison D, Styles B. A guide to running randomised controlled trials for educational researchers. Natl Found Educ Res. 2010;1–65.

55. Kheirollahi F, Rahimi Z, Arsang-Jang S, Sharifirad G, Sarraf P, Gharlipour Z. Puberty health status among adolescent girls: A model- based educational program. Int J Pediatr. 2017;5(7):5369–78.

56. Arasteh FE, Shobeiri F, Parsa P, Mohamadi Y. Effect of group counseling on adolescent girls in improving knowledge and practice of menstrual hygiene in welfare boarding centers. J Postgrad Med Inst. 2019;33(1):46–51.

57. Phillips-Howard PA, Nyothach E, Ter Kuile FO, Omoto J, Wang D, Zeh C, et al. Menstrual cups and sanitary pads to reduce school attrition, and sexually transmitted and reproductive tract infections: A cluster randomised controlled feasibility study in rural Western Kenya. BMJ Open. 2016;

58. Van Eijk AM, Sivakami M, Thakkar MB, Bauman A, Laserson KF, Coates S, et al. Menstrual hygiene management among adolescent girls in India: A Systematic review and meta-analysis. BMJ Open. 2016;6(3).

59. United Nations Children's Fund (UNICEF) and World Health Organization 2018. Drinking Water, Sanitation and Hygiene in Schools : Global baseline report 2018. Unicef. 2018. 82 p.

60. Bhagwat A, Jijina P. A Psychosocial Lens on an Indigenous Initiative to Address Menstrual Health and Hygiene in Indian Villages. Soc Work Public Health. 2020;35(3):73–89.

61. Harris K, Kneale D, Tj L, Vm M, Grigg J, Thomas J, et al. School-based self-management interventions for asthma in children and adolescents : a mixed methods systematic review (Review). 2019;

62. DfE. Relationships Education, Relationships and Sex Education (RSE) and Health Education. 2019;(November):1–6.

63. Freeman S, Eddy SL, McDonough M, Smith MK, Okoroafor N, Jordt H, et al. Active learning increases student performance in science, engineering, and mathematics. Proc Natl Acad Sci U S A. 2014;111(23):8410–5.

64. Dewey J. Experience and Education [1938]. 1938;

65. Vygotsky L. Interaction between learning and development. Readings Dev Child. 1978;23(3):34–41.

66. Achkovska Leshkovska E, Miovska Spaseva S. John Dewey'S educational theory and

[Type here]

For peer review only

educational implications of Howard Gardner's multiple intelligences theory. Int J Cogn Res Sci Eng Educ. 2016;4(2):57–66.

67. Yilmaz SK, Bohara AK, Chowdhury SR. Touch for Health: Use of Pavlovian Processes with Physical Touch as a Means to Improve Menstrual Hygiene Management Initiatives, Measured by Willingness to Pay. PharmacoEconomics - Open. 2020;4(2):263–76.

68. Lock A, Strong T, editors. Introduction. In: Social Constructionism: Sources and Stirrings in Theory and Practice. Cambridge: Cambridge University Press; 2010. p. 1–11.

ADDITIONAL INFORMATION

Contributors

RE, FG and BH designed the review. RE and OC reviewed titles, abstracts and full-texts for eligibility. Disagreement was resolved by discussion and where necessary FG and BH offered their view. RE, FG and BH agreed on a data extraction framework, which was then carried out by RE. The quality assessment tool was agreed upon by RE, FG and BH. RE used the MMAT to assess the quality and this was verified by FG and BH. RE prepared the manuscript and it was reviewed and edited by FG and BH.

Funding Statement

This work was supported by the National Institute for Health Research Unit on Improving Health in Slums. Funder reference: 16/136/87.

Ethical Approval

BSREC 43/19-20

Competing Interests

None declared

Patient Consent

None required

Data Sharing Statement

No original data were generated in this study

Word Count 4186

For peer review only

For peer review only - http://bmjopen.bmj.com/site/about/guidelines.xhtml

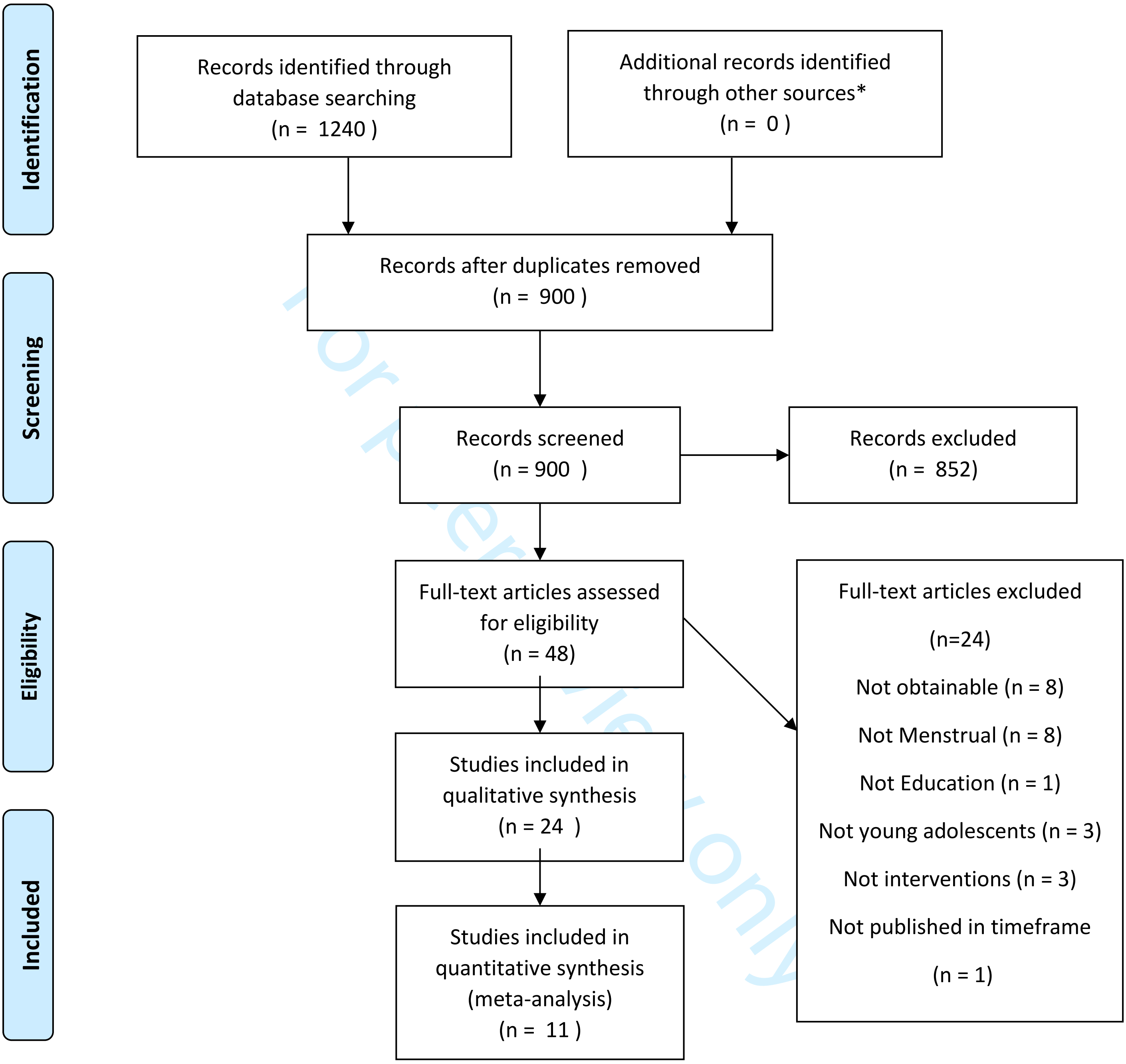

Figure 2. PRISMA flow chart

*grey literature

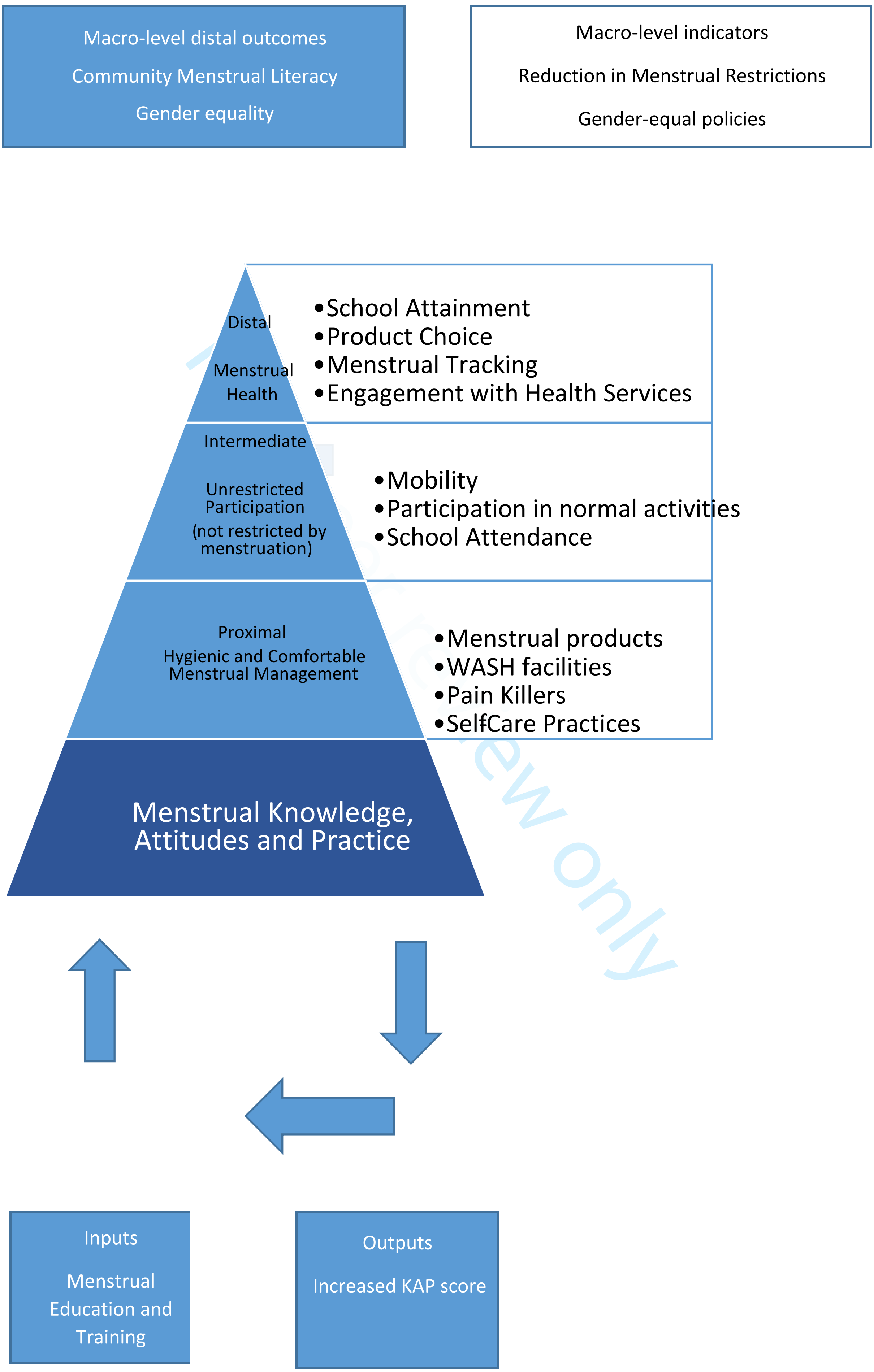
Macro-level distal outcomes
Community Menstrual Literacy
Gender equality
Macro-level indicators
Reduction in Menstrual Restrictions
Gender-equal policies
Distal
Menstrual Health
•School Attainment
•Product Choice
•Menstrual Tracking
•Engagement with Health Services
Intermediate
Unrestricted Participation
(not restricted by menstruation)
•Mobility
•Participation in normal activities
•School Attendance
Proximal
Hygienic and Comfortable Menstrual Management
•Menstrual products
•WASH facilities
•Pain Killers
•SelfCare Practices
Menstrual Knowledge, Attitudes and Practice
Inputs
Menstrual Education and Training
Outputs
Increased KAP score

Supplementary File: Characteristics and quality of included studies

Citation	Design	Country	Study population	Sampling Method	No of participants	Type of intervention	MMAT Clear Research Questions /2	MMAT Appropriate Data Collection?/2	MMAT Supplementary questions relevant to methodology/5	MMAT Total/9	MMAT RAG rating
(1)	RCT quasi-experimental pre-test, post test	Iran	Mothers and adolescent school girls	Random Cluster-sampling	364	Half hour lecture and pamphlet distribution to improve attitude	2	1	5	8	Green
(2)	Quantitative Descriptive Case Series Quasi-experimental pre-test, post-test	Turkey	Intellectually-disabled girls in special education training centre	convenience	77	Demonstration on doll to improve Pad-replacement skills	2	2	4	8	Green
(3)	Quantitative descriptive one group pre-test, post-test	Iran	All adolescent girls in two welfare	Purposive sampling	30	Group counselling in boarding centres	2	2	3	7	Amber

For peer review only

			boarding centres								
(4)	Quantitative descriptive case report; pre-test, post-test	Indonesia	Mild-intellectually disabled girl	Purposive sampling	1	Stories and video-modelling	2	2	3	7	Amber
(5)	Mixed methods, quantitative survey pre-test, post-test	Ethiopia	Girls enrolled in school in grades 6 and 7	Cluster randomised approach	636	Puberty Book 'Growth and Changes'	2	2	5	9	Green
(6)	RCT, pre-test, post-test	India	Adolescent high school girls aged 11 – 19 years of Zilla Parishad High School	Systematic random technique (every other girl on register)	250	Chalk and talk, posters, flipcharts,	1	2	2	5	Red
(7)	Quantitative descriptive one group, Pre-test, post-test	Bangladesh	adolescent female students aged 11–16 years, in grade 6–8, and living with their parents.	Random cluster sampling	416	Field manual training	2	2	3	7	Amber
(8)	Qualitative understanding of quasi-RCT	Uganda	school girls in eight study schools in primary classses grades 4 - 7.	Quasi-randomised cluster sampling	27: education (n=8) pads alone (n=8) education and pads (n=6) and	75 minute session of Straight Talk Education programme	2	2	5	9	Green

For peer review only

					control (n=5)						
(9)	RCT pre-test post-test	USA	Children of both sexes whose parents had registered an interest	Random sampling	80 (43 girls)	'A New You, that's Who' video series	2	2	3	7	Amber
(10)	3 arm RCT Control and 2 intervention: peer and small group teaching pre-test/post-test	Iran	Adolescent school girls	Random sampling of three high schools	90	Small group and peer-teaching	2	2	3	7	Amber
(11)	Mixed methods Longitudinal study	Uganda	Girls and boys in secondary school	Purposive selection of schools	369	Multi-component approach to optimising government guidelines for puberty education	2	2	5	9	Green
(12)	Quasi-experimental RCT, and pre-test, post- test	Iran	School girls	Random cluster sampling	152	A model-based educational program	1	2	2	5	Red
(13)	Quasi-experimental RCT	Iran	School girls aged 14 – 16 years and their parents	Random cluster sampling	159	Teaching programme with discussion	2	2	3	7	Amber
(14)	four-armed quasi-experimental RCT Pre and post test	Uganda	Girls in grades 3 - 5	Randomised	1124	Educational arm used Straight Talk	1	2	0	3	Amber

For peer review only

(15)	Mixed Methods Quantitative cross-sectional surveys and qualitative focus group	Nepal	Girls in grades seven to ten in 28 large schools.	Random cluster sampling	860	WASH in Schools (WinS) programme	1	2	4	7	Amber
(16)	Mixed methods process evaluation Longitudinal study	Uganda	Girls and boys in secondary school	Purposive selection of schools	369	Multi-component approach to optimising government guidelines for puberty education	2	2	5	9	Green
(17)	3-arm single-site open cluster randomised controlled pilot study.	Kenya	Primary girls aged 14 -16	Random cluster sampling	644	Puberty education and menstrual cup instruction from nurse	1	2	5	8	Green
(18)	Quantitative non-random post-test only	India	Adolescent girls	Purposive (schools) and random (girls)	2206	Monthly discussion group	2	2	5	9	Green
(19)	Quasi-experimental Control/intervention pre-test post-test	Indonesia	Girls aged 9-12 pre-menarch	Stratified random sampling technique	174	Booklet	2	2	2	6	Amber
(20)	Experimental; Intervention and control; pre-test, post-test	India	Adolescent girls at a government high school	Randomly sampled	50	One training session on menstrual hygiene	1	0	1	2	Red
(21)	Quantitative descriptive Pre-test, post-test	India	Adolescent girls	Multi-level stratified	2564	‘model’ schools received	1	2	5	8	Green

For peer review only

				sampling of schools		additional WASH support and education embedded into curriculum					
(22)	A quasi-experimental RCT two group pretest-posttest design	China	Adolescent girls	Purposive sampling of schools	116	Researcher – led Menstrual Hygiene Class	2	2	4	8	Green
(23)	Mixed methods. Qualitative review and semi-quantitative measure of cup use nested in larger RCT	Kenya	Post-menarche adolescent girls	Random allocation of 10 schools to menstrual cup arm	192	Puberty education and menstrual cup instruction from nurse	1	2	5	8	Green
(24)	RCT three arm – educating girls, educating mothers or control. pre and post test	Iran	Post-menarche adolescent school girls	12 purposively sampled schools	327	30 minute lecture on puberty hygiene plus a booklet	0	1	5	6	Amber

Summary of characteristics of included studies

Framework analysis and the ‘best fit’ principle were used to score the studies (25–27). All studies were interrogated with two questions ‘Are there clear research questions?’ and ‘Do the collected data allow the research questions to be addressed?’ which were considered fundamental to the quality and were scored on a scale of ‘Yes’ = 2, ‘not clear’ = 1 and ‘No’ = 0. Five further supplementary questions were considered that addressed quality issues such as sample size. The sets of questions were different depending upon the study design, and are not directly comparable, so less weight was given to these; Yes = 1 and No = 0. The maximum score when added together was 2 + 2 + 5 = 9. Studies scored 0-5 were categorised as low quality (as it was possible to get these scores without clear research questions or valid methods); those that scored 6 or 7 were scored as moderate quality and those that scored 8 or 9 were scored as high quality (a subjective scale based on personal expertise (27) and community of practice validation (28).

REFERENCES

For peer review only

1. Afsari A, Mirghafourvand M, Valizadeh S, Abbasnezhadeh M, Galshi M, Fatahi S. The effects of educating mothers and girls on the girls' attitudes toward puberty health: A randomized controlled trial. Int J Adolesc Med Health. 2015;2015.

2. Altundağ S, Çalbayram NÇ. Teaching menstrual care skills to intellectually disabled female students. Mol Ecol. 2016;25(13–14):1962–8.

3. Arasteh FE, Shobeiri F, Parsa P, Mohamadi Y. Effect of group counseling on adolescent girls in improving knowledge and practice of menstrual hygiene in welfare boarding centers. J Postgrad Med Inst. 2019;33(1):46–51.

4. Ariyanti TD, Royanto LRM. The Effectiveness of Social Stories and Video Modeling in Improving Self-Care Skills in Female Adolescents with Mild Intellectual Disabilities during Menstrual Periods. 2018;135(Iciap 2017):189–99.

5. Blake S, Boone M, Yenew Kassa A, Sommer M. Teaching Girls About Puberty and Menstrual Hygiene Management in Rural Ethiopia: Findings From a Pilot Evaluation. J Adolesc Res. 2018;33(5):623–46.

6. Rani Chadalawada U, Devi S A, Rani M S. Effect of Health Education on Adolescent Girls Regarding Knowledge About Menstruation. J Evol Med Dent Sci. 2017;6(13):1040–3.

7. Haque SE, Rahman M, Itsuko K, Mutahara M, Sakisaka K. The effect of a school-based educational intervention on menstrual health: An intervention study among adolescent girls in Bangladesh. BMJ Open. 2014;4(7):1–9.

8. Hennegan J, Montgomery P. Do menstrual hygiene management interventions improve education and psychosocial outcomes for women and girls in low and middle income countries? A systematic review. PLoS One. 2016;11(2):1–22.

9. Hurwitz LB, Lovato SB, Lauricella AR, Woodruff TK, Patrick E, Wartella E. "A New You, That's Who": an evaluation of short videos on puberty and human reproduction. Palgrave Commun. 2018;4(1).

10. Jarrahi R, Golmakani N, Mazlom SR. Effect of menstrual hygiene education based on peer and small group teaching methods on hygiene behaviors in female adolescents: A comparative study. Evid Based Care J. 2020;10(1):70–4.

11. Kansiime C, Hytti L, Nalugya R, Nakuya K, Namirembe P, Nakalema S, et al. Menstrual health intervention and school attendance in Uganda (MENISCUS-2): A pilot intervention study. BMJ Open. 2020;10(2):1–11.

12. Kheirollahi F, Rahimi Z, Arsang-Jang S, Sharifirad G, Sarraf P, Gharlipour Z. Puberty health status among adolescent girls: A model- based educational program. Int J Pediatr. 2017;5(7):5369–78.

13. Mokari H, Khaleghparast S, Samani LN. Impact of Puberty Health Education on Anxiety of Adolescents. Int J Med Res Heal Sci. 2016;5(5, S, SI):284–91.

For peer review only

14. Montgomery P, Hennegan J, Dolan C, Wu M, Steinfield L, Scott L. Menstruation and the Cycle of Poverty: A Cluster Quasi-Randomised Control Trial of Sanitary Pad and Puberty Education Provision in Uganda. PLoS One. 2016;11(12).

15. Morrison J, Basnet M, Bhatt A, Khimbanjar S, Chaulagain S, Sah N, et al. Girls' menstrual management in five districts of Nepal: Implications for policy and practice. Stud Soc Justice. 2018;12(2):251–72.

16. Nalugya R, Tanton C, Hytti L, Kansiime C, Nakuya K, Namirembe P, et al. Assessing the effectiveness of a comprehensive menstrual health intervention program in Ugandan schools (MENISCUS): process evaluation of a pilot intervention study. Pilot Feasibility Stud. 2020;6(1):1–15.

17. Phillips-Howard PA, Nyothach E, Ter Kuile FO, Omoto J, Wang D, Zeh C, et al. Menstrual cups and sanitary pads to reduce school attrition, and sexually transmitted and reproductive tract infections: A cluster randomised controlled feasibility study in rural Western Kenya. BMJ Open. 2016;

18. Ramaiya A, Malhotra A, Cronin C, Stevens S, Kostizak K, Sharma A, et al. How does a Social and Behavioral Change Communication Intervention Predict Menstrual Health and Hygiene Management: A Cross-Sectional Study. BMC Public Health. 2019;19(1):1–12.

19. Setyowati S, Rizkia M, Ungsianik T. Improving Female Adolescents' Knowledge, Emotional Response, and Attitude toward Menarche following Implementation of Menarcheal Preparation Reproductive Health Education. Asian/Pacific Isl Nurs J. 2019;4(2):84–91.

20. Sharma R, Sandhya N, Deepika K, Varsha S, Vardha. Menstrual hygiene among adolescent girls. Indian J Community Heal. 2015;27(3):376–80.

21. Sivakami M, van Eijk AM, Thakur H, Kakade N, Patil C, Shinde S, et al. Effect of menstruation on girls and their schooling, and facilitators of menstrual hygiene management in schools: Surveys in government schools in three states in India, 2015. J Glob Health. 2019;9(1).

22. Su JJ, Lindell D. Promoting the menstrual health of adolescent girls in China. Nurs Heal Sci. 2016;18(4):481–7.

23. Van Eijk AM, Sivakami M, Thakkar MB, Bauman A, Laserson KF, Coates S, et al. Menstrual hygiene management among adolescent girls in India: A Systematic review and meta-analysis. BMJ Open. 2016;6(3).

24. Valizadeh S, Assdollahi M, Mirghafourvand M, Afsari A. Educating Mothers and Girls about Knowledge and Practices toward Puberty Hygiene in Tabriz, Iran: A Randomized Controlled Clinical Trial. Iran Red Crescent Med J. 2017;19(2).

25. Carroll C, Booth A, Cooper K. A worked example of "best fit" framework synthesis: A systematic review of views concerning the taking of some potential chemopreventive agents. BMC Med Res Methodol. 2011;11.

26. Carroll C, Booth A, Leaviss J, Rick J. "best fit" framework synthesis: Refining the method. BMC Med Res Methodol. 2013;13(1).

27. Suto I. A critical review of some qualitative research methods used to explore rater cognition. Educ Meas Issues Pract. 2012;31(3):21–30.

28. Bejar II. A validity-based approach to quality control and assurance of automated scoring. Assess Educ Princ Policy Pract. 2011;18(3):319–41.

For peer review only

For peer review only

PRISMA 2020 Checklist

Section and Topic	Item #	Checklist item	Location where item is reported
**TITLE**			
Title	1	Identify the report as a systematic review.	1
**ABSTRACT**			
Abstract	2	See the PRISMA 2020 for Abstracts checklist.	
**INTRODUCTION**			
Rationale	3	Describe the rationale for the review in the context of existing knowledge.	3-4
Objectives	4	Provide an explicit statement of the objective(s) or question(s) the review addresses.	4
**METHODS**			
Eligibility criteria	5	Specify the inclusion and exclusion criteria for the review and how studies were grouped for the syntheses.	4
Information sources	6	Specify all databases, registers, websites, organisations, reference lists and other sources searched or consulted to identify studies. Specify the date when each source was last searched or consulted.	5
Search strategy	7	Present the full search strategies for all databases, registers and websites, including any filters and limits used.	5
Selection process	8	Specify the methods used to decide whether a study met the inclusion criteria of the review, including how many reviewers screened each record and each report retrieved, whether they worked independently, and if applicable, details of automation tools used in the process.	5
Data collection process	9	Specify the methods used to collect data from reports, including how many reviewers collected data from each report, whether they worked independently, any processes for obtaining or confirming data from study investigators, and if applicable, details of automation tools used in the process.	6
Data items	10a	List and define all outcomes for which data were sought. Specify whether all results that were compatible with each outcome domain in each study were sought (e.g. for all measures, time points, analyses), and if not, the methods used to decide which results to collect.	6
	10b	List and define all other variables for which data were sought (e.g. participant and intervention characteristics, funding sources). Describe any assumptions made about any missing or unclear information.	6
Study risk of bias assessment	11	Specify the methods used to assess risk of bias in the included studies, including details of the tool(s) used, how many reviewers assessed each study and whether they worked independently, and if applicable, details of automation tools used in the process.	6
Effect measures	12	Specify for each outcome the effect measure(s) (e.g. risk ratio, mean difference) used in the synthesis or presentation of results.	6-7
Synthesis methods	13a	Describe the processes used to decide which studies were eligible for each synthesis (e.g. tabulating the study intervention characteristics and comparing against the planned groups for each synthesis (item #5)).	7
	13b	Describe any methods required to prepare the data for presentation or synthesis, such as handling of missing summary statistics, or data conversions.	7
	13c	Describe any methods used to tabulate or visually display results of individual studies and syntheses.	7
	13d	Describe any methods used to synthesize results and provide a rationale for the choice(s). If meta-analysis was performed, describe the model(s), method(s) to identify the presence and extent of statistical heterogeneity, and software package(s) used.	7
	13e	Describe any methods used to explore possible causes of heterogeneity among study results (e.g. subgroup analysis, meta-regression).	
	13f	Describe any sensitivity analyses conducted to assess robustness of the synthesized results.	
Reporting bias assessment	14	Describe any methods used to assess risk of bias due to missing results in a synthesis (arising from reporting biases).	7
Certainty	15	Describe any methods used to assess certainty (or confidence) in the body of evidence for an outcome.	7

For peer review only

PRISMA 2020 Checklist

Section and Topic	Item #	Checklist item	Location where item is reported
assessment			
**RESULTS**			
Study selection	16a	Describe the results of the search and selection process, from the number of records identified in the search to the number of studies included in the review, ideally using a flow diagram.	8
	16b	Cite studies that might appear to meet the inclusion criteria, but which were excluded, and explain why they were excluded.	
Study characteristics	17	Cite each included study and present its characteristics.	9
Risk of bias in studies	18	Present assessments of risk of bias for each included study.	SF
Results of individual studies	19	For all outcomes, present, for each study: (a) summary statistics for each group (where appropriate) and (b) an effect estimate and its precision (e.g. confidence/credible interval), ideally using structured tables or plots.	12
Results of syntheses	20a	For each synthesis, briefly summarise the characteristics and risk of bias among contributing studies.	12
	20b	Present results of all statistical syntheses conducted. If meta-analysis was done, present for each the summary estimate and its precision (e.g. confidence/credible interval) and measures of statistical heterogeneity. If comparing groups, describe the direction of the effect.	12
	20c	Present results of all investigations of possible causes of heterogeneity among study results.	SF
	20d	Present results of all sensitivity analyses conducted to assess the robustness of the synthesized results.	SF
Reporting biases	21	Present assessments of risk of bias due to missing results (arising from reporting biases) for each synthesis assessed.	SF
Certainty of evidence	22	Present assessments of certainty (or confidence) in the body of evidence for each outcome assessed.	SF
**DISCUSSION**			
Discussion	23a	Provide a general interpretation of the results in the context of other evidence.	16
	23b	Discuss any limitations of the evidence included in the review.	17
	23c	Discuss any limitations of the review processes used.	17
	23d	Discuss implications of the results for practice, policy, and future research.	17
**OTHER INFORMATION**			
Registration and protocol	24a	Provide registration information for the review, including register name and registration number, or state that the review was not registered.	17
	24b	Indicate where the review protocol can be accessed, or state that a protocol was not prepared.	
	24c	Describe and explain any amendments to information provided at registration or in the protocol.	
Support	25	Describe sources of financial or non-financial support for the review, and the role of the funders or sponsors in the review.	26
Competing interests	26	Declare any competing interests of review authors.	26
Availability of data, code and other materials	27	Report which of the following are publicly available and where they can be found: template data collection forms; data extracted from included studies; data used for all analyses; analytic code; any other materials used in the review.	

For peer review only

PRISMA 2020 Checklist

From: Page MJ, McKenzie JE, Bossuyt PM, Boutron I, Hoffmann TC, Mulrow CD, et al. The PRISMA 2020 statement: an updated guideline for reporting systematic reviews. BMJ 2021;372:n71. doi: 10.1136/bmj.n71
For more information, visit: http://www.prisma-statement.org/

For peer review only
